# Origin of the 3-methylglutaryl moiety in caprazamycin biosynthesis

**DOI:** 10.1186/s12934-022-01955-6

**Published:** 2022-11-05

**Authors:** Daniel Bär, Benjamin Konetschny, Andreas Kulik, Houchao Xu, Davide Paccagnella, Patrick Beller, Nadine Ziemert, Jeroen S. Dickschat, Bertolt Gust

**Affiliations:** 1 Department of Pharmaceutical Biology, Eberhard-Karls University Tübingen, Auf der Morgenstelle 8, 72076 Tübingen, Germany; 2 Department of Microbial Bioactive Compounds, Interfaculty Institute of Microbiology and Infection Medicine, Eberhard-Karls University Tübingen, Auf der Morgenstelle 28, 72076 Tübingen, Germany; 3 Kekulé-Institute for Organic Chemistry and Biochemistry, University of Bonn, Gerhard-Domagk-Straße 1, 53121 Bonn, Germany; 4 Interfaculty Institute of Microbiology and Infection Medicine, Institute for Bioinformatics and Medical Informatics, Eberhard-Karls University Tübingen, Auf der Morgenstelle 28, 72076 Tübingen, Germany; 5 German Center for Infection Research (DZIF), Partner Site Tübingen, Tübingen, Germany

**Keywords:** *Streptomyces coelicolor*, Caprazamycin, Leucine/isovalerate utilization pathway, 3-methylglutaryl-CoA, Primary metabolism

## Abstract

**Background:**

Caprazamycins are liponucleoside antibiotics showing bioactivity against Gram-positive bacteria including clinically relevant *Mycobacterium tuberculosis* by targeting the bacterial MraY-translocase. Their chemical structure contains a unique 3-methylglutaryl moiety which they only share with the closely related liposidomycins. Although the biosynthesis of caprazamycin is understood to some extent, the origin of 3-methylglutaryl-CoA for caprazamycin biosynthesis remains elusive.

**Results:**

In this work, we demonstrate two pathways of the heterologous producer *Streptomyces coelicolor* M1154 capable of supplying 3-methylglutaryl-CoA: One is encoded by the caprazamycin gene cluster itself including the 3-hydroxy-3-methylglutaryl-CoA synthase Cpz5. The second pathway is part of primary metabolism of the host cell and encodes for the leucine/isovalerate utilization pathway (Liu-pathway). We could identify the *liu* cluster in *S. coelicolor* M1154 and gene deletions showed that the intermediate 3-methylglutaconyl-CoA is used for 3-methylglutaryl-CoA biosynthesis. This is the first report of this intermediate being hijacked for secondary metabolite biosynthesis. Furthermore, Cpz20 and Cpz25 from the caprazamycin gene cluster were found to be part of a common route after both individual pathways are merged together.

**Conclusions:**

The unique 3-methylglutaryl moiety in caprazamycin originates both from the caprazamycin gene cluster and the leucine/isovalerate utilization pathway of the heterologous host. Our study enhanced the knowledge on the caprazamycin biosynthesis and points out the importance of primary metabolism of the host cell for biosynthesis of natural products.

**Supplementary Information:**

The online version contains supplementary material available at 10.1186/s12934-022-01955-6.

## Background

**Fig. 1:**
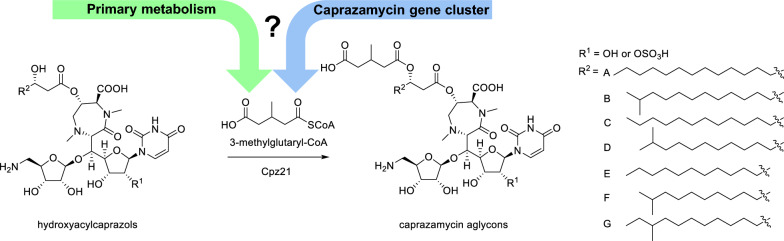
Chemical structure of hydroxyacylcaprazols and caprazamycins aglycons. Attachment of 3-methylglutaryl-CoA is catalyzed by Cpz21

Caprazamycins (CPZs) belong to the family of liponucleoside antibiotics and were first isolated from *Streptomyces sp.* MK730-62F2 [1]. Their chemical core structure is built up by ( +)-caprazol which consists of 5′-glycyluridine, 5-amino-d-ribose and a permethylated diazepanone ring. β-hydroxylated fatty acids are attached to the diazepanone ring resulting in formation of the biosynthetic intermediates hydroxyacylcaprazols. Attached to the free hydroxyl group of the fatty acid is a 3-methylglutarate bound to a permethylated l-rhamnose, affording the final caprazamycins. Caprazamycins are classified by chain length and constitution of the fatty acid [2, 3] (Fig. 1).

Their bioactivity is based on inhibition of phospho-MurNAc-pentapeptide transferase (MraY), which transfers phospho-MurNAc-pentapeptide from UDP-MurNAc-pentapeptide onto undecaprenyl phosphate (C55-P) during bacterial cell wall biosynthesis [4–6]. Caprazamycin B shows promising activity against Gram-positive bacteria including strains of the genus *Mycobacterium*, such as the clinically relevant pathogen *M. tuberculosis* [1, 7]. Although it was reported for liposidomycins, that the 3-methylglutaryl moiety increases inhibition of peptidoglycan synthesis, newly generated caprazamycin derivates were focused on truncating the chemical structure thus sacrificing this moiety [8]. In palmitoylcaprazol, the fatty acid residue was replaced by a simpler palmitoyl side chain resulting in similar potency against *M. smegmatis* and *M. tuberculosis* compared to caprazamycin B [9, 10]. Further studies showed that the key scaffold for antimicrobial activity consists of uridine, aminoribose, the diazepanone ring and the fatty acid chain, whereas the 3-methylglutaryl moiety and the rhamnose seemed to be dispensable [10, 11]. For CPZEN-45, replacing the fatty acid moiety by 4-butylanilide showed improved efficacy and lower toxicity compared to caprazamycins, but this compound shifted its target from MraY to WecA in *M. tuberculosis* [7, 12, 13].

The caprazamycin gene cluster was the first cluster of a MraY inhibitor to be identified and verified by heterologous expression in *S. coelicolor* M512. Later, the biosynthetic gene cluster of the closely related liposidomycins was reported [14, 15]. Genes required for biosynthesis of rhamnose are not located on the CPZ gene cluster but were identified elsewhere on the genome of *Streptomyces sp.* MK730-62F2. Since the heterologous host *S. coelicolor* is missing those genes, heterologous expression resulted in the formation of caprazamycin aglycons instead of caprazamycins [16]. Deletion of the carboxylesterase Cpz21 resulted in the formation of hydroxyacylcaprazols indicating Cpz21 to be responsible for the transfer of 3-methylglutaryl-CoA onto the β-hydroxyl group of hydroxyacylcaprazols [14].

Although the steps of caprazamycin biosynthesis are understood to some extent, no biosynthetic route leading to 3-methylglutaryl-CoA has been described so far [17]. Investigating the caprazamycin gene cluster, a starting point for 3-methylglutaryl-CoA biosynthesis could be catalyzed by the putative 3-hydroxy-3-methylglutaryl-CoA synthase Cpz5. This class of enzymes usually converts acetyl-CoA and acetoacetyl-CoA to 3-hydroxy-3-methylglutaryl-CoA [18–20]. A removal of the hydroxyl group would then lead to the desired 3-methylglutaryl-CoA.

Considering that precursors for natural products could also be provided by the host cell itself, we analyzed the primary metabolism of *S. coelicolor* M1154 for plausible intermediates leading to 3-methylglutaryl-CoA as well. A rich source of short-chain acyl-CoAs is the degradation of branched-chain amino acids (BCAA) leucine, valine and isoleucine [21]. The first step in degradation of all three branched-chain amino acids is transamination followed by oxidative decarboxylation to the corresponding acyl-CoA-thioesters carried out by a branched-chain α-keto acid dehydrogenase (BCDH) complex [22–24]. Next, this pathway diverges into three branches, one for each amino acid [21]. The leucine/isovalerate utilization pathway (Liu-pathway) of *P. aeruginosa* PAO1 is well described and it was also investigated in *P. putida* PpG2, *M. xanthus* DK1622 and *M. luteus* [25–32] (Additional file 1: Fig. S1). A comparative genomics study of the Liu-pathway regulation showed that this cluster is also widely distributed among protobacteria [33]. The catabolic pathway of leucine continues with isovaleryl-CoA being converted to 3-methylcrotonyl-CoA in a dehydrogenation step catalyzed by isovaleryl-CoA dehydrogenase LiuA. Enzymes facilitating this step have also been reported for *S. coelicolor* J802 encoded by *acdH* (*sco2779*) and for *S. avermitilis* ATCC 31272 encoded by *fadE4* (*SAVERM_5275*) [34]. Next, a 3-methylcrotonyl-CoA carboxylase complex consisting of subunits α and β (LiuD and LiuB) generates 3-methylglutaconyl-CoA by transfer of acetyl-CoA onto 3-methylcrotonyl-CoA. The identification of a similar α-subunit was also reported for *S. toxytricini* [35]. 3-methylglutaconyl-CoA is further converted to 3-hydroxy-3-methylglutaryl-CoA by 3-methylglutaconyl-CoA hydratase LiuC and in a final step, the 3-hydroxy-3-methylglutaryl-CoA lyase LiuE generates acetyl-CoA and acetoacetate. Intriguingly, all intermediates of the Liu-pathway resembling 3-methylglutaryl-CoA in structure making them promising starting points for a biosynthetic route towards 3-methyglutaryl-CoA originating from the host cells primary metabolism.

In this work, we could show that two routes are leading to 3-methylglutaryl-CoA for caprazamycin biosynthesis. The first is encoded by the caprazamycin gene cluster and starts by the action of the 3-hydroxy-3-methylglutaryl-CoA synthase Cpz5. The second pathway derives from the catabolism of leucine as part of the primary metabolism of the host cell. Our study enhances the knowledge on the biosynthesis of liponucleoside antibiotics and lays the foundation for further studies on the alteration of the 3-methylglutaryl moiety towards new caprazamycin analogues.

## Results

### Deletion of the 3-hydroxy-3-methylglutaryl-CoA synthase Cpz5 does not abolish caprazamycin aglycon formation

The biosynthetic gene cluster of caprazamycins encodes for Cpz5, a putative 3-hydroxy-3-methylglutaryl-CoA synthase (Additional file 1: Fig. S2). This family of enzymes catalyze the Claisen-like condensation of acetyl-CoA and acetoacetyl-CoA to 3-hydroxy-3-methylglutaryl-CoA. So far, the role of Cpz5 during caprazamycin biosynthesis has not been investigated. Due to the structural similarity to 3-methylglutaryl-CoA, we predicted that 3-hydroxy-3-methylglutaryl-CoA generated by Cpz5 could be a promising starting point of a biosynthetic route towards 3-methylglutaryl-CoA entirely encoded by the caprazamycin gene cluster. To test this hypothesis, a deletion of *cpz5* was generated on cosmid cpzLK09, which contains the entire caprazamycin gene cluster, yielding cosmid cpzDB04. Heterologous expression of cpzLK09 in *S. coelicolor* leads to the accumulation of caprazamycin aglycons because the heterologous host is lacking the genes required to produce l-rhamnose, whereas a mutant not capable of providing 3-methylglutaryl-CoA should stop production at the stage of hydroxyacylcaprazols [16]. We generated three individual mutants either containing cpzLK09 resulting in *S. coelicolor* M1154/cpzLK09 (1)–(3) or harboring cpzDB04 resulting in *S. coelicolor* M1154/cpzDB04 (1)–(3). As expected, all three *S. coelicolor* M1154/cpzLK09 mutants were able to produce caprazamycin aglycons, showing signals with high intensities for caprazamycin aglycons A and B with *m/z* 958.5 at Rt of 14.9 min, caprazamycin aglycons C, D and G with *m/z* 944.5 at Rt of 14.3 min and caprazamycin aglycons E and F with *m/z* 930.5 at Rt of 13.8 min. Masses of hydroxyacylcaprazols A and B with *m/z* 830.5 expected at Rt of 14.0 min, hydroxyacylcaprazols C, D and G with *m/z* 816.5 expected at Rt of 13.4 min and hydroxyacylcaprazols E and F with *m/z* 802.5 expected at Rt of 12.9 min were not detected. In contrast to our hypothesis, a deletion of *cpz5* could not impair the formation of caprazamycin aglycons as they were still produced by all three gene deletion mutants (Additional file 1: Figs. S3–S5). Those findings indicated that *cpz5* is not exclusively responsible for 3-methylglutaryl-CoA formation.

### Identification of the leucine/isovalerate utilization pathway as precursor supply for 3-methylglutaryl-CoA biosynthesis

**Fig. 2:**
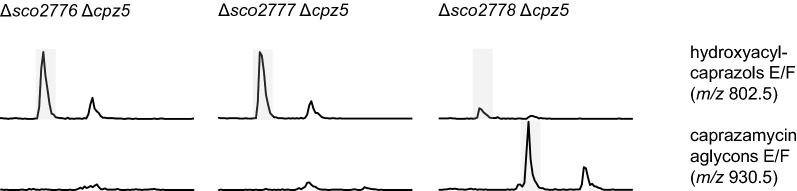
Extracted ion chromatograms from extracts of *S. coelicolor* M1154 mutants with either *sco2776*, *sco2777* or *sco2778* deleted and harboring a caprazamycin gene cluster containing a *cpz5* deletion. Retention time from 12 to 16 min is shown. Captions indicate mutant genotypes. Highlighted are signals for hydroxyacylcaprazols E/F with *m/z* of 802.5 and caprazamycin aglycons E/F with *m/z* of 930.5 acquired in positive mode. Non-highlighted signals represent sulfated hydroxyacylcaprazols E/F or sulfated caprazamycin aglycons E/F respectively

Gene deletion could not confirm *cpz5* as the sole source of 3-methylglutaryl-CoA for caprazamycin biosynthesis. To discover other pathways for the generation of this intermediate, we investigated the primary metabolism of *S. coelicolor* M1154 in more detail. Since 3-methylglutaryl-CoA is not described to be part of a primary metabolism pathway so far, we decided to look for primary metabolism pathways processing plausible precursors of this compound, including 3-hydroxy-3-methylglutaryl-CoA, that can be readily transformed into 3-methylglutaryl-CoA. One promising lead were degradation pathways of amino acids, as they are ubiquitously distributed among bacteria and intermediates of their degradation processes are short-chain acyl-CoAs similar to 3-methylglutaryl-CoA. Utilization of leucine and isovalerate was described for *Pseudomonas aeruginosa* PAO1 in more detail [26–29]. In this strain, a gene cluster encoding for a reaction cascade called leucine/isovalerate utilization pathway was identified to process leucine and isovalerate to acetoacetate and acetyl-CoA. This gene cluster consists of six genes encoding for an isovaleryl-CoA dehydrogenase (*liuA*), two subunits of a 3-methylcrotonyl-CoA carboxylase (*liuB* and *liuD*), a 3-methylglutaconyl-CoA dehydratase (*liuC*), a 3-hydroxy-3-methylglutaryl-CoA lyase (*liuE*) and a transcriptional regulator (*liuR*). BLAST analysis of *S. coelicolor* M1154 revealed genes homologue to *liuA* (*sco2779*), *liuB* (*sco2776*), *liuD* (*sco2777*) and *liuE* (*sco2778*) (Additional file 1: Fig. S6). No homologue was found for *liuC* in this cluster though, raising the question if this gene is located elsewhere on the genome. Interestingly, besides the missing *liuC*, the *liu* homologues in *S. coelicolor* are ordered in a different genetic organization compared to *P. aeruginosa* PAO1. We found the same genetic organization in caprazamycin wildtype producer *S. sp.* MK730-62F2 and it was also reported for *S. avermitilis* ATCC 31267 [36]. A cblaster analysis using the *liu* cluster from *S. coelicolor* M1154 including the putative regulator *sco2775* as query revealed, that only 4.5% of all *Actinobacteria* strains but more than 52% of *Streptomyces* strains listed in the Genome Taxonomy Database (GTDB) possess homologues of all five query genes. (Additional file 1: Fig. S7A). To investigate if the Liu-pathway is connected to caprazamycin biosynthesis, we deleted *sco2776* to *sco2779* in the heterologous host *S. coelicolor* M1154 resulting in *S. coelicolor* M1154Δ*sco2776*-*sco2779*. Successful deletion and absence of wildtype genes was verified by PCR and sequencing. Introduction of either cpzLK09 or cpzDB04 into this strain resulted in mutants *S. coelicolor* M1154Δ*sco2776-sco2779*/cpzLK09 (1)–(3) and *S. coelicolor* M1154Δ*sco2776-sco2779/*cpzDB04 (1)–(3). Successful cosmid integration was verified by PCR and sequencing. HPLC–MS analysis revealed that production of caprazamycin aglycons was abolished when both *cpz5* and *sco2776-sco2779* were deleted, whereas production of caprazamycin aglycons was still possible if only *sco2776-sco2779* were missing but *cpz5* was still intact (Additional file 1: Figs. S8, S9). This indicates that both pathways are generating 3-methylglutaryl-CoA independently. Formation of the correct product was confirmed by HPLC–MSMS (Additional file 1: Fig. S10). To find out which exact intermediate of the Liu-pathway is hijacked for caprazamycin biosynthesis, we next generated mutants with single deletions of *sco2776*, *sco2777*, *sco2778* or *sco2779* resulting in *S. coelicolor* M1154Δ*sco2776, S. coelicolor* M1154Δ*sco2777, S. coelicolor* M1154Δ*sco2778* and *S. coelicolor* M1154Δ*sco2779*. Successful deletion and absence of wildtype genes was again verified by PCR and sequencing. Introduction of cpzLK09 resulted in mutants *S. coelicolor* M1154Δ*sco2776/*cpzLK09 (1)–(3)*,* *S. coelicolor* M1154Δ*sco2777/*cpzLK09 (1)–(3)*,* *S. coelicolor* M1154Δ*sco2778/*cpzLK09 (1)–(3) and *S. coelicolor *M1154Δ*sco2779/*cpzLK09 (1)–(3) whereas introduction of cpzDB04 led to mutants *S. coelicolor *M1154Δ*sco2776/*cpzDB04 (1)-(3), *S. coelicolor *M1154Δ*sco2777/*cpzDB04 (1)–(3), *S. coelicolor* M1154Δ*sco2778/*cpzDB04 (1)–(3) and *S. coelicolor* M1154Δ*sco2779/*cpzDB04 (1)–(3). Successful cosmid integration was verified by PCR and sequencing. As expected, all mutants containing a complete caprazamycin gene cluster were still capable of producing caprazamycin aglycons. With *cpz5* deleted on the caprazamycin gene cluster, mutants with a missing *sco2776* or *sco2777* ceased caprazamycin aglycon production and accumulated hydroxyacylcaprazols instead, whereas a deletion of *sco2778* or *sco2779* had no impact on caprazamycin aglycon formation (Fig. 2 and Additional file 1: Figs. S11–S18).

Those results strongly suggest, that either 3-methylglutaconyl-CoA or 3-hydroxy-3-methylglutaryl-CoA is the common precursor for 3-methylglutaryl-CoA for both the gene cluster encoded pathway and the pathway originating from primary metabolism.

### Two dehydrogenases are converting isovaleryl-CoA to 3-methylcrotonyl-CoA

**Fig. 3:**
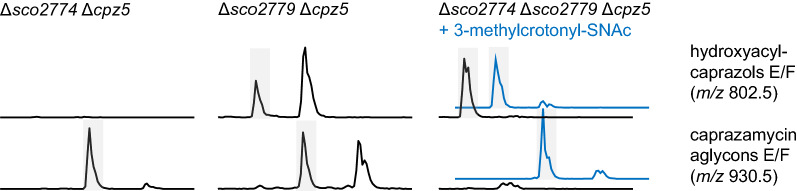
Extracted ion chromatograms from extracts of *S. coelicolor* M1154 mutants with either *sco2774*, *sco2779* or both deleted and harboring a caprazamycin gene cluster containing a *cpz5* deletion. Retention time from 12 to 16 min is shown. Captions indicate mutant genotypes. Highlighted are signals for hydroxyacylcaprazols E/F with *m/z* of 802.5 and caprazamycin aglycons E/F with *m/z* of 930.5 acquired in positive mode. Non-highlighted signals represent sulfated hydroxyacylcaprazols E/F or sulfated caprazamycin aglycons E/F respectively. Extract of a culture supplemented with 3-methylcrotonyl-SNAc is shown in blue

Surprisingly, a single deletion of *sco2779* together with a *cpz5*-deletion did not abolish caprazamycin aglycon production, although we predicted this gene to encode for the enzyme catalyzing the first step in the Liu-pathway. To verify that 3-methylcrotonyl-CoA is truly a precursor in the biosynthesis of 3-methylglutaryl-CoA, we synthesized (1-^13^C)-3-methylcrotonyl-SNAc. Feeding this compound to *S. coelicolor* M1154/cpzLK09 resulted in a change of isotope distribution towards heavier caprazamycin aglycons, indicating that (1-^13^C)-3-methylglutaryl-SNAc was incorporated and therefore, 3-methylcrotonyl-CoA is utilized for caprazamycin biosynthesis. (Additional file 1: Fig. S19). Due to its location on the *liu* operon, *sco2779* presumably encodes for an acyl-CoA dehydrogenase capable of converting isovaleryl-CoA to 3-methylcrotonyl-CoA, but our gene deletion experiment suggested that it is not the sole enzyme catalyzing this step. Thus, we searched for other genes annotated as acyl-CoA dehydrogenases on the genome of *S. coelicolor* A3(2) in the StrepDB database and found 38 candidates that could possibly complement *sco2779* [37]. A BLAST search revealed 23 homologues for this gene located on the *S. coelicolor* M1154 genome with identities ranging from about 20% to 40%. The most promising candidate turned out to be *sco2774*. This gene is located just next to the *liu* cluster and is orientated in the same direction as the cluster’s putative regulator *sco2775* (Additional file 1: Fig. S6). To clarify the roles of *sco2774* and *sco2779*, we generated mutants with a single deletion of *sco2774* resulting in *S. coelicolor* M1154Δ*sco2774* and a mutant with a deletion of both *sco2774* and *sco2779* resulting in *S. coelicolor* M1154Δ*sco2774*Δ*sco2779*. Successful deletion and absence of wildtype genes was again verified by PCR and sequencing. After introduction of either cpzLK09 or cpzDB04, caprazamycin production of mutants *S. coelicolor* M1154Δ*sco2774*/cpzLK09 (1)–(3), *S. coelicolor* M1154Δ*sco2774*/cpzDB04 (1)–(3), *S. coelicolor* M1154Δ*sco2774*Δ*sco2779*/cpzLK09 (1)–(3) and *S. coelicolor* M1154Δ*sco2774*Δ*sco2779*/cpzDB04 (1)–(3) was analyzed by HPLC–MS. As expected, mutants containing the complete caprazamycin gene cluster were still capable of producing caprazamycin aglycons. A single deletion of *sco2774* together with a *cpz5* deletion did not impair caprazamycin aglycon production. However, if both *sco2774* and *sco2779* were deleted together with *cpz5*, hydroxyacylcaprazols accumulated instead (Fig. 3 and Additional file 1: Figs. S20–S23).

These findings strongly suggest that both acyl-CoA dehydrogenases are converting isovaleryl-CoA to 3-methylcrotonyl-CoA. To support this thesis, we performed a chemical complementation by adding 3-methylcrotonyl-SNAc to a culture of M1154Δ*sco2774*Δ*sco2779*/cpzDB04. HPLC–MS analysis revealed that caprazamycin aglycon biosynthesis could be restored by addition of 3-methylcrotonyl-SNAc, indicating a successful restoration of the Liu-pathway (Fig. 3 and Additional file 1: Fig. S24). Since our data suggests that *sco2774* is an extension of the *liu* cluster, we performed a cblaster analysis with this extended cluster as query sequence. Interestingly, 48.2% of S*treptomyces* strains still contained all six query genes compared to 52.2% of the query without *sco2774*, indicating that a *liu* cluster with a second acyl-CoA dehydrogenase is commonly distributed in *Streptomyces* (Additional file 1: Fig. S7B).

### Involvement of the acyl-CoA synthase Cpz20 and the dehydrogenase Cpz25 in 3-methylglutaryl-CoA biosynthesis

**Fig. 4:**
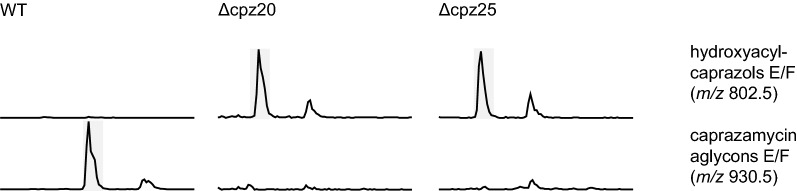
Extracted ion chromatograms from extracts of *S. coelicolor* M1154 mutants harboring a caprazamycin gene cluster containing a deletion of either *cpz20* or *cpz25*. Retention time from 12 to 16 min is shown. Highlighted are signals for hydroxyacylcaprazols E/F with *m/z* of 802.5 and caprazamycin aglycons E/F with *m/z* of 930.5 acquired in positive mode. Non-highlighted signals represent sulfated hydroxyacylcaprazols E/F or sulfated caprazamycin aglycons E/F respectively

So far, our results narrowed down precursor candidates for 3-methylglutaryl-CoA to either 3-methylglutaconyl-CoA synthesized by Sco2776 and Sco2778 or 3-hydroxy-3-methylglutaryl-CoA generated by Cpz5. A conversion of 3-methylglutaconyl-CoA to 3-methylglutaryl-CoA requires a reduction of the C–C double bond. The caprazamycin gene cluster encodes for Cpz25 which is a promising candidate for this reduction step. A BLAST analysis revealed that Cpz25 belongs to the medium chain reductase/dehydrogenase (MDR)/zinc-dependent alcohol dehydrogenase-like family of proteins (cd05188) and contains a conserved domain of enoyl-reductases from polyketide synthases (smart00829). Another enzyme that could be involved in 3-methylglutaryl-CoA biosynthesis is a putative acyl-CoA synthase encoded by *cpz20*. To investigate if *cpz20* and *cpz25* do in fact play a role in 3-methylglutaryl-CoA biosynthesis, both genes were deleted individually on the caprazamycin gene cluster, resulting in cosmids cpzDB05 and cpzDB06. Subsequent transfer into *S. coelicolor* M1154 resulted in mutants *S. coelicolor* M1154/cpzDB05 (1)–(3) and *S. coelicolor* M1154/cpzDB06 (1)-(3). Successful cosmid integration was verified by PCR and sequencing and caprazamycin production was analyzed by HPLC–MS. Neither *cpz20* nor *cpz25* deficient mutants were able to accumulate caprazamycin aglycons. However, hydroxyacylcaprazols were still produced, indicating that both *cpz20* and *cpz25* are indeed essential for supplying 3-methylglutaryl-CoA (Fig. 4 and Additional file 1: Figs. S25, S26).

Intriguingly, both *cpz20* and *cpz25* deletions were heterologously expressed in a *S. coelicolor* M1154 strain with *sco2774*-*sco2779* still intact. This strongly suggests that both enzymes are not specific for either one of the 3-methylglutaryl-CoA supply pathways. We predict that Cpz25 is the junction that merges both pathways into one common route by reduction of 3-methylglutaconyl-CoA and Cpz20 could be involved in an additional activation related step prior transfer onto hydroxyacylcaprazols by Cpz21.

### Two pathways supply 3-methylglutaryl-CoA for caprazamycin biosynthesis

**Scheme 1:**
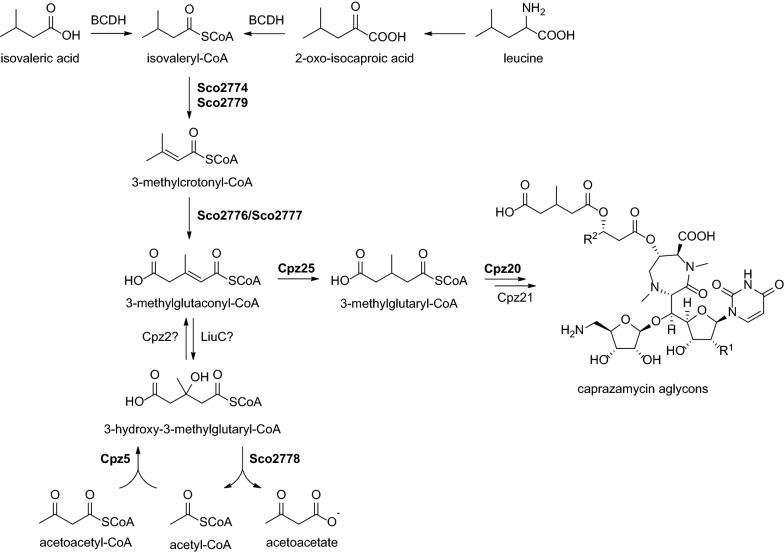
Proposed biosynthesis model for the 3-methylglutaryl moiety in caprazamycin biosynthesis. In bold: Steps investigated in this study. BCDH: Branched-chain α-keto acid dehydrogenase complex. Sco2774: acyl-CoA dehydrogenase. Sco2779: isovaleryl-CoA dehydrogenase (LiuA). Sco2776: 3-methylcrotonyl-CoA carboxylase subunit β (LiuB). Sco2777: 3-methylcrotonyl-CoA carboxylase subunit α (LiuD). Sco2778: 3-hydroxy-3-methylglutaryl-CoA lyase (LiuE). Cpz5: 3-hydroxy-3-methylglutaryl-CoA synthase. Cpz20: acyl-CoA synthase. Cpz21: carboxylesterase. Cpz25: reductase

Based on the results obtained in this study, we are able to propose the first model of the origin of 3-methylglutaryl-CoA for caprazamycin biosynthesis (Scheme 1).

Our investigation identified two pathways involved in supplying this precursor. First, the degradation of branched-chain amino acids leucine and isovalerate as part of the hosts primary metabolism. Second, a pathway encoded on the caprazamycin gene cluster itself. Surprisingly, these two pathways are not fully independent from each other. The leucine/isovalerate degradation ends with generating acetyl-CoA and acetoacetate by action of the 3-hydroxy-3-methylglutaryl-CoA lyase Sco2779. The caprazamycin gene cluster encoded pathway starts with a similar reverse reaction catalyzed by the 3-hydroxy-3-methylglutaryl-CoA synthase Cpz5 utilizing acetoacetyl-CoA and acetyl-CoA continuing to work in the Liu-pathway’s opposite direction until it reaches 3-methylglutaconyl-CoA. We identified this compound as the central intermediate where both pathways fuse and continue on a joint route. This route starts with a reduction step in which 3-methylglutaconyl-CoA is converted to the desired 3-methylglutaryl-CoA. Moreover, our results strongly suggest that Cpz20, a putative acyl-CoA synthase, is also required on this route because a single deletion of *cpz20* ceased caprazamycin aglycon production the same way as a *cpz25* deletion did. Finally, 3-methylglutaryl-CoA is transferred onto the hydroxyacylcaprazols by Cpz21 [14].

## Discussion

### The branched-chain amino acids degradation pathways as versatile suppliers of precursors

**Fig. 5:**
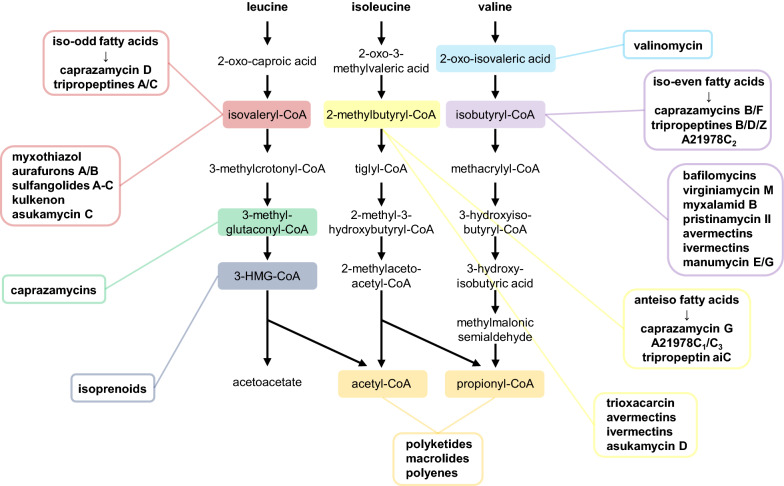
Degradation pathways of branched-chain amino acids leucine, isoleucine and valine and natural products utilizing intermediates of those pathways for their biosynthesis

The catabolism of branched-chain amino acids is a rich source of precursors for metabolites and natural products. It starts with leucine, isoleucine and valine undergoing transamination and subsequent oxidative decarboxylation by the branched-chain dehydrogenase complex resulting in isovaleryl-CoA, 2-methylbutyryl-CoA and isobutyryl-CoA (Fig. 5).

Important products of these intermediates are branched-chain fatty acids [38]. Caprazamycins rely on all three of these intermediates by incorporating iso-even, iso-odd and anteiso fatty acids [2]. Our work showed that the 3-methylglutaryl moiety of caprazamycins is derived from another intermediate of leucine degradation, 3-methylglutaconyl-CoA, revealing an additional dependency of caprazamycin biosynthesis on this primary metabolism pathway. Members of A21978C, a complex of lipopeptide antibiotics including the clinically relevant daptomycin produced by *S. roseosporus*, and the tripropeptines produced by *Lysobacter sp.* BMK333-48F3 incorporate branched-chain fatty acids as well [39–41]. Iso-odd branched-chain fatty acids are further important for cell membrane fluidity and comprise about 75% of all fatty acids in *Myxobacteria* [42]. *Stigmatella urantiaca* DW4/3–1 utilizes isovaleryl-CoA as an unusual starter unit for biosynthesis of myxothiazol and aurafuron A and B [43–45]. The same starter unit is used for sulfangolides A-C and the closely related kulkenon isolated from different strains of *Sorangium cellulosum* [46]. Usage of the unusual extender unit 2-carboxy-3-hydroxy-5-methylhexanoyl-CoA derived from isovaleryl-CoA was reported for leupyrrin biosynthesis in *S. cellulosum* So ce690 [47]. An alternative pathway to isovaleryl-CoA was described in *M. xanthus* which reverses the Liu-pathway resembling the caprazamycin gene cluster encoded pathway for 3-methylglutaryl-CoA biosynthesis [48]. This alternative pathway contains a 3-hydroxy-3-methylglutaryl-CoA synthase (MvaS) similar to Cpz5 generating 3-hydroxy-3-methylglutaryl-CoA from acetoacetyl-CoA and acetyl-CoA [49]. Surprisingly, the next step in this cascade is catalyzed by the LiuC homologue of *M. xanthus* itself, indicating that LiuC is able to switch direction of catalysis depending on the metabolic state of the cell [50]. Since no LiuC homologue exists in the *liu* cluster of *S. coelicolor* M1154, we identified candidate genes homologue to LiuC from *M. xanthus* elsewhere on the genome that could fill the gap of the caprazamycin gene cluster encoded pathway. However, single gene inactivation by transposon insertion of those candidate genes (*sco1838*, *sco4384*, *sco4930* and *sco6732*) in a Liu-pathway deficient background could not impair caprazamycin aglycon production in a mutant harboring the complete caprazamycin gene cluster (Additional file 1: Figs. S27–S30). This raises the question whether the correct gene was amongst our candidates for inactivation, if more than one gene is responsible for the LiuC catalyzed reaction or if the LiuC homologue in *S. coelicolor* is not able to catalyze the reaction in both directions as it is the case in *M. xanthus*. Small adaption of an BCAA intermediate prior to incorporation is an observation we made for 3-methylglutaconyl-CoA converted by Cpz25 during this work. Another example is found in the biosynthesis of cyclic peptide valinomycin in *Streptomyces sp.* M10. This biosynthesis requires d-hydroxyisovaleric acid, which is afforded via reduction of 2-oxo-isovaleric acid, the first intermediate of valine degradation, by action of a d-hydroxyisovalerate dehydrogenase. In the same strain, production of the antifungal-active bafilomycins competes for 2-oxo-isovaleric acid by utilizing its BCDH product isobutyryl-CoA [51]. Isobutyryl-CoA was further suggested as the starter unit for the biosynthesis of virginiamycin M in *S. virginiae*, myxalamid B in *Stigmatella aurantiaca* Sg a15 and *Myxococcus xanthus* DK1622 and pristinamycin II in *S. pristinaespiralis* [52–54]. Trioxacarcin, first isolated from S. *bottropensis* DO-45, rather uses 2-methylbutyryl-CoA derived from isoleucine building an unusual spiro-epoxide structure which is believed to bind DNA as part of its mode of action [55, 56]. Avermectins and their hydrogenated derivates ivermectins, antiparasitic compounds isolated from *S. avermitilis*, carrying either a 2-methylbutyryl or isobutyryl moiety, relying their production on the degradation of isoleucine or valine [22, 57, 58]. Members of the manumycin family are distinguished by the utilized PKS starter unit including all three: isovaleryl-CoA, 2-methylbutyryl-CoA and isobutyryl-CoA [59, 60].

The end products of BCAA catabolism, propionyl-CoA and acetyl-CoA, are versatile starter units for PKS derived natural products. Those include therapeutically important antimicrobial polyketides such as erythromycin and antifungal polyenes such as nystatin [61–63]. Although acetyl-CoA can be obtained from several sources including glycolysis, studies in *S. coelicolor* demonstrated that branched-chain amino acid degradation is an important pathway for acetyl-CoA supply for actinorhodin biosynthesis [24]. However, overexpression of the BCDH cluster aiming to elevate methylmalonyl-CoA levels increased pikromycin production only by 1.3-fold, whereas overexpression of methylmalonyl-CoA mutase achieved a 1.7-fold increase, pointing out a possible limitation of methylmalonyl-CoA supply by this pathway [64].

Despite the numerous natural products derived from either BCDH complex intermediates or the end products of BCAA degradation, surprisingly little is known about other BCAA degradation intermediates being utilized for secondary metabolite biosynthesis. The mevalonate pathway leading to the large group of isoprenoids relies on 3-hydroxy-3-methylglutaryl-CoA [18]. However, the mevalonate pathway is equipped with a 3-hydroxy-3-methylglutaryl-CoA synthase similar to Cpz5 generating 3-hydroxy-3-methylglutaryl-CoA and is therefore not dependent on BCAA degradation. In this work, we report for the first time that the leucine/isovalerate degradation pathway intermediate 3-methylglutaconyl-CoA is being utilized as a precursor for natural product biosynthesis.

### The caprazamycin gene cluster provides 3-methylglutaryl-CoA from acetyl-CoA and acetoacetyl-CoA

The 3-methylglutaryl in caprazamycin is a unique moiety only found in the closely related liposidomycins, muraminomicins, A-84830A and A-90289A [65, 66]. The gene cluster of liposidomycin was identified in *S. sp.* SN-1061 M. Since liposidomycins share the same core structure as caprazamycins and only differ in the fatty acid chain composition and absence of the rhamnosyl moiety, both clusters share highly similar genetic organization and homology [14, 15]. The same biosynthetic machinery leading to 3-methylglutaryl-CoA we discovered in this work could also be assembled by the liposidomycin gene cluster encoding for the 3-hydroxy-3-methylglutaryl-CoA synthase LpmA (81/89% identity/similarity to Cpz5), the acyl-CoA synthase LpmR (88/91% identity/similarity to Cpz20) and the acyl dehydrogenase LpmW (91/95% identity/similarity to Cpz25). The biosynthetic gene cluster of A-90289 in *S. sp.* SANK 60,405 encodes for LipC (82/88% identity/similarity to Cpz5), LipQ (88/92% identity/similarity to Cpz20) and LipV (90/95% identity/similarity to Cpz25) [67]. Although the muraminomicin gene cluster encodes for Mra13 (87/90% identity/similarity to Cpz20) and Mra8 (88/93% identity/similarity to Cpz25), it lacks a homologue of *cpz5*, raising the question if this biosynthesis relies exclusively on primary metabolism for 3-methylgutaryl-CoA precursor supply [68].

One step in the caprazamycin gene cluster encoded pathway is dehydration of 3-hydroxy-3-methylglutaryl-CoA generating 3-methylglutaconyl-CoA. As already described, we could not identify a gene from the heterologous host *S. coelicolor* M1154 responsible this reaction. The caprazamycin gene cluster itself encodes with the putative dehydratase *cpz2* for another candidate suitable for catalyzing this reaction [14]. However, a single deletion of *cpz2* did not interfere with production of caprazamycin aglycons in a Liu-pathway deficient background (Additional file 1: Fig. S31). Furthermore, analyzing the gene clusters of liposidomycin, A-90289A and muraminomicins, no homologues of *cpz2* could be identified [15, 67, 68]. It remains unclear if *cpz2* is participating in the generation of 3-methylglutaconyl-CoA along with other enzymes from the primary metabolism or if other enzymes are responsible for this dehydration step.

So far, no other natural products than the liposidomycin family of nucleoside antibiotics are known to contain a 3-methylglutaryl moiety. We could show that generating this moiety is fully dependent on genes located on the caprazamycin gene cluster. However, a degradation pathway for 4-methylbenzoyl-CoA utilized under anaerobic conditions was reported for *Magnetospirillum sp.* pMbN1. An intermediate of this degradation process is indeed 3-methylgluaryl-CoA which is converted to 3-methylglutaconyl-CoA followed by the same steps as shown for the Liu-pathway, revealing that 3-methylglutaryl-CoA can also be part of the cell’s metabolic pathway [69]. Finding new pathways involving 3-methylglutaryl-CoA and investigating the strains containing these could be a promising starting point for the discovery of novel natural products containing this unusual moiety.

## Material and methods

### Bacterial strains and culture conditions

*Escherichia coli* DH5α (Thermo Fisher Scientific) was used as general cloning host (Additional file 1: Table S1). *E. coli* BW25113/pIJ790 was used for Red/ET-mediated recombination and *E. coli* BT340 facilitated FLP-recombination [70–72]. *E. coli* ET12567 was used for triparental conjugation [73]. *E. coli* DH5α was generally cultivated in LB-medium or on LB-agar plates at 37° C. *E. coli* BW25113/pIJ790 and *E. coli* BT340 were cultivated as described before [70]. *Streptomyces coelicolor* M1154 was used for heterologous expression of the caprazamycin gene cluster [74]. *Streptomyces* strains were generally cultivated on mannitol soya flour (MS) agar plates supplemented with MgCl_2_ (1 mg/ml) or in tryptone soy broth (TSB) at 30° C [75]. Apramycin (50 µg/ml), carbenicillin (100 µg/ml), chloramphenicol (25 µg/ml), kanamycin (50 µg/ml), nalidixic acid (25 µg/ml) and tetracycline (5 µg/ml) were added if required.

### DNA isolation and manipulation

Isolation and manipulation of DNA was carried out according to standard procedures described for *E. coli* and *Streptomyces* [75, 76].

### Generation of *liu* deletion mutants

An apramycin resistance cassette was amplified from plasmid pIJ773 using primer pairs *liuA_F* and *liuA_R, liuB_F* and *liuB_R, liuD_F* and *liuD_R, liuE_F* and *liuE_R* or *sco2774_773_FW* and *sco2774_773_RV* (Additional file 1: Table S2). The purified cassettes were introduced into electro-competent *E. coli* ET12567/pIJ790/StC105 to replace the corresponding gene by Red/ET-mediated recombination, respectively. The resulting cosmids were verified by restriction digest and PCR using primer pairs *LiuA_verify_1154_neu_F* and *LiuA_verify_1154_neu_R*, *LiuB_verify_1154_neu2_F* and *LiuB_verify_1154_neu2_R*, *LiuD_verify_1154_neu_F* and *LiuD_verify_1154_neu_R*, *LiuE_verify_1154_neu_F* and *LiuE_verify_1154_neu_R* or *sco2774_verify_FW* and *sco2774_verify_RV*. FLP-mediated excision of the resistance cassette was achieved by introducing the cosmids into *E. coli* BT340. Obtained cosmids were verified by restriction digest, PCR and sequencing of the resulting PCR products using the same primer pairs as above. A tetracycline-*oriT*-cassette was amplified from plasmid pIJ787 using primer pairs *bla-oriT_cassette_787* and *bla-tet_cassette_787* [77]. The purified cassette was introduced into *E. coli* ET12567/pIJ790 containing the desired *liu* deletion cosmid followed by Red/ET-mediated recombination. The resulting cosmids were verified by restriction digest, PCR and sequencing using primer pairs *bla_tet_oriT_v4_F* and *bla_tet_oriT_v4_R*. Final cosmids were introduced into *E. coli* ET12567 followed by triparental conjugation into *S. coelicolor* M1154 using *E. coli* ET12567/pR9406. Dilution series of single exconjugants were cultivated on MS-agar plates supplemented with nalidixic acid (25 µg/ml) and MgCl_2_ (1 mg/ml) for several rounds until complete loss of the kanamycin resistance, indicating a successful double crossover event. Deletion of the desired gene was verified by PCR and sequencing using genomic DNA as template and primer pairs as above. Absence of wildtype gene was tested by PCR using primer pairs *LiuA_in-out_1154_F* and *LiuA_in-out_1154_R*, *LiuB_in-out_1154_F* and *LiuB_in-out_1154_R*, *LiuD_in-out_1154_neu_F* and *LiuD_in-out_1154_neu_R*, *LiuE_in-out_1154_neu_F* and *LiuE_in-out_1154_neu_R* or *sco2774_in-out_FW* and *sco2774_in-out_RV*.

### Generation of mutants with transposon insertions

Cosmids containing transposon insertions were obtained from Paul Dyson (Swansea University, UK) and introduced into *E. coli* ET12567 followed by triparental conjugation into *S. coelicolor M1154*Δ*sco2776-2779* using *E. coli* ET12567/pR9406 [78]. Dilution series of single exconjugants were cultivated on MS-agar plates containing apramycin (50 µg/ml) nalidixic acid (25 µg/ml) and MgCl_2_ (1 mg/ml) for several rounds until complete loss of the kanamycin resistance, indicating a successful double crossover event. Inactivation of the desired gene was verified by PCR and sequencing using genomic DNA as template and primer pairs *SCO1838_tra_ver_FW* and *SCO1838_tra_ver_RV*, *SCO4384_tra_ver_FW* and *SCO4384_tra_ver_RV*, *SCO4930_tra_ver_FW* and *SCO4930_tra_ver_RV* or *SCO6732_tra_ver_FW* and *SCO6732_tra_ver_RV*. Absence of wildtype gene was tested by PCR using primer pairs *SCO1838_tra_ver_FW* and *1838_gene_RV*, *SCO4384_tra_ver_FW* and *4384_gene_RV*, *SCO4930_tra_ver_FW* and *4930_gene_RV* or *SCO6732_tra_ver_FW* and *6732_gene_RV*.

### Generation of *cpz* deletion mutants

An apramycin resistance cassette was amplified from plasmid pIJ773 using primer pairs *cpz2_773_FW* and *cpz2_773_RV*, *cpz5_773_F* and *cpz5_773_R*, *cpz20_773_F* and *cpz20_773_R* or *cpz25_773_F* and *cpz25_773_R*, respectively. The purified cassettes were introduced into electro-competent *E. coli* ET12567/pIJ790/cpzLK09 to replace the correspondent gene by Red/ET-mediated recombination. The resulting cosmids were verified by restriction digest and PCR using primer pairs *cpz2_verify_FW* and *cpz2_verify_RV*, *cpz5_verifyKO_F* and *cpz5_verifyKO_R, cpz20_verifyKO_F* and *cpz20_verifyKO_R* or *cpz25_verifyKO_F* and *cpz25_verifyKO_R.* FLP-mediated excision of the resistance cassette was achieved by introducing the cosmids into *E. coli* BT340. Resulting cosmids were verified by restriction digest, PCR and sequencing using the same primer pairs as above. Final cosmids were introduced into *E. coli* ET12567 followed by triparental conjugation into *S. coelicolor* M1154 mutants using *E. coli* ET12567/pR9406. Dilution series of single exconjugants were cultivated on MS-agar plates supplemented with nalidixic acid (25 µg/ml), kanamycin (50 µg/ml) and MgCl_2_ (1 mg/ml) for several rounds. Successful introduction of the caprazamycin gene cluster was verified by PCR and sequencing using genomic DNA as template and primer pairs as above.

### Production and extraction of secondary metabolites

For production of caprazamycin aglycons or hydroxyacylcaprazols, 10–20 µl of *Streptomyces* spores were cultivated in 2 ml of TSB for 2 days at 30° C and 200 rpm. 100 µl of the preculture was transferred into 3 ml of P-medium (10 g/L soytone, 10 g/L soluble starch, 20 g/L d-maltose) supplemented with 150 µl trace elements solution (40 mg/L ZnCl_2_, 200 mg/L FeCl_3_ × 6 H_2_O, 10 mg/L CuCl_2_ × 2 H_2_O, 10 mg/L MnCl_2_ × 4 H_2_O, 10 mg/L Na_2_B_4_O_6_ × 10 H_2_O and 10 mg/L (NH_4_)_6_Mo_7_O_24_ × 4 H_2_O) in a 24-square deepwell plate and cultivated for another 5–7 days at 30° C and 200 rpm [79]. 3-methylcrotonyl-SNAc or 1-^13^C-3-methylcrotonyl-SNAc were added to a final concentration of 0.8 mM, if required. To extract secondary metabolites, 1 ml of culture supernatant was adjusted to pH of 4 with 1 M HCl, 1 ml of n-butanol was added, mixed vigorously followed by centrifugation (13,000 rpm, 4° C, 15 min). The n-butanol phase was separated, evaporated and the residue resuspended in 500 µl methanol.

### Synthesis of 3-methylcrotonyl-SNAc (1) and (1-^13^C)-3-methylcrotonyl-SNAc (5)

To a solution of 3,3-dimethylacrylic acid (0.50 g, 5.0 mmol) in CH_2_Cl_2_ (20 mL) was added *N*,*N*′-dicyclohexylcarbodiimide (1.14 g, 5.5 mmol; Additional file 1: Fig. S32). Then the reaction mixture was cooled to 0 °C, followed by the addition of 4-dimethylaminopyridine (0.12 g, 1.0 mmol) and *N*-acetylcysteamine (0.48 g, 4.0 mmol). After stirring at room temperature for 2 h, the reaction was quenched by adding aq. HCl (1 m, 20 mL) and then extracted with EtOAc (3 × 20 mL). The combined organic phases were dried with MgSO_4_ and concentrated under reduced pressure. The residue was purified by column chromatography on silica gel (EtOAc: cyclohexane = 3: 1) to give 3-methylcrotonyl-SNAc (**1**) as white solid.

**3-Methylcrotonyl-SNAc (1)** TLC (EtOAc: cyclohexane = 3: 1): *R*_f_ = 0.20; yield: 0.58 g (2.9 mmol, 72%); ^1^H-NMR (500 MHz, CDCl_3_): δ = 5.91 (m, 1H), 5.88 (br s, 1H), 3.35 (dt, ^3^*J*_H,H_ = 6.5, 5.7 Hz, 2H), 2.94 (dd ^3^*J*_H,H_ = 6.8, 5.8 Hz, 2H), 2.06 (d, ^4^*J*_H,H_ = 1.2 Hz, 3H), 1.86 (s, 3H), 1.79 (d, ^4^*J*_H,H_ = 1.3 Hz, 3H) ppm; ^13^C-NMR (126 MHz, CDCl_3_): δ = 189.6 (C), 170.5 (C), 155.1 (C), 123.1 (CH), 40.1 (CH_2_), 28.5 (CH_2_), 27.4 (CH_3_), 23.4 (CH_3_), 21.4 (CH_3_) ppm.

Ethyl (1-^13^C)bromoacetate (2.00 g, 11.9 mmol) and triethyl phosphite (1.98 g, 11.9 mmol) were added to a reaction flask. The reaction mixture was refluxed at 130 °C for 8 h to obtain triethyl (1-^13^C)phosphonoacetate (**2**) which was used in the following reaction without further purification. A solution of NaH (60% in mineral oil, 0.18 g, 4.4 mmol) in THF (12 mL) was cooled to 0 °C and **2** (1.00 g, 4.4 mmol) was then added dropwise. After stirring at 0 °C for 15 min, acetone (0.26 g, 4.4 mmol) was added. Stirring of the reaction mixture was continued at 0 °C for 30 min, followed by stirring at room temperature for 7 h. The reaction was quenched with sat. aq. NH_4_Cl solution (20 mL) and then extracted with Et_2_O (3 × 20 mL). The organic layers were combined, dried with MgSO_4_ and concentrated under reduced pressure. The residue was purified by column chromatography on silica gel (petrol ether: Et_2_O = 20: 1) to give ethyl (1-^13^C)-3,3-dimethylacrylate (**3**) as colorless oil.

**Ethyl (1-**^**13**^**C)-3,3-dimethylacrylate (3)** TLC (petrol ether: Et_2_O = 10: 1): *R*_f_ = 0.41; yield: 0.45 g (3.5 mmol, 79%); ^1^H-NMR (500 MHz, CDCl_3_): δ = 5.57 (m, 1H), 4.04 (qd, ^3^*J*_H,H_ = 7.1, ^3^*J*_C,H_ = 3.0 Hz, 2H), 2.06 (dd, ^4^*J*_H,H_ = 1.2 Hz, ^3^*J*_C,H_ = 1.2 Hz, 3H), 1.79 (d, ^4^*J*_H,H_ = 1.3 Hz, 3H), 1.17 (t, ^3^*J*_H,H_ = 7.1 Hz, 3H) ppm. ^13^C-NMR (126 MHz, CDCl_3_): δ = 166.8 (^13^C), 156.4 (d, ^2^*J*_C,C_ = 2.2 Hz, C), 116.2 (d, ^1^*J*_C,C_ = 75.7 Hz, CH), 59.5 (d, ^2^*J*_C,C_ = 2.3 Hz, CH_2_), 27.4 (d, ^3^*J*_C,C_ = 7.6 Hz, CH_3_), 20.2 (d, ^3^*J*_C,C_ = 1.5 Hz, CH_3_), 14.4 (d, ^3^*J*_C,C_ = 2.2 Hz, CH_3_) ppm.

To an aq. KOH solution (0.5 n, 5 mL) was added **3** (39 mg, 0.3 mmol). The reaction mixture was refluxed at 100 °C overnight until the oil layer disappeared. Then aq. HCl solution (1 n, 10 mL) was added and the reaction mixture was extracted with Et_2_O (3 × 10 mL). The organic layers were combined, washed with brine (10 mL), dried with MgSO_4_ and then concentrated under reduced pressure to give (1-^13^C)-3,3-dimethylacrylic acid (**4**) which was used in the following reaction without further purification. Compound **4** and *N*-acetylcysteamine (35 mg, 0.3 mmol) were added to CH_2_Cl_2_ (3 mL). The solution was then cooled to 0 °C, followed by the addition of 1-ethyl-3-(3-dimethylaminopropyl)carbodiimid (58 mg, 0.3 mmol) and 4-dimethylaminopyridine (7 mg, 0.06 mmol). After stirring at room temperature overnight, the reaction solution was diluted with EtOAc (20 mL), washed with sat. aq. NH_4_Cl solution, sat. aq. NaHCO_3_ solution and brine, dried with MgSO_4_, and then concentrated under reduced pressure. The residue was purified by column chromatography on silica gel (EtOAc) to give (1-^13^C)-3-methylcrotonyl-SNAc (**5**) as white solid.

**(1-**^**13**^**C)-3-Methylcrotonyl-SNAc (5)** Yield: 45 mg (0.22 mmol, 74%); ^1^H-NMR (500 MHz, CDCl_3_): δ = 5.91 (m, 1H), 5.83 (br s, 1H), 3.35 (dt, ^3^*J*_H,H_ = 6.6, 5.7 Hz, 2H), 2.95 (ddd, ^3^*J*_H,H_ = 7.1, 5.8 Hz, ^3^*J*_C,H_ = 4.7 Hz, 2H), 2.06 (dd, ^4^*J*_H,H_ = 1.2 Hz, ^3^*J*_C,H_ = 1.2 Hz, 3H), 1.86 (s, 3H), 1.79 (d, ^4^*J*_H,H_ = 1.3 Hz, 3H) ppm; ^13^C-NMR (126 MHz, CDCl_3_): δ = 189.6 (^13^C), 170.4 (C), 155.1 (C), 123.1 (d, ^1^*J*_C,C_ = 63.5 Hz, CH), 40.2 (CH_2_), 28.5 (CH_2_), 27.4 (d, ^3^*J*_C,C_ = 7.3 Hz, CH_3_), 23.4 (CH_3_), 21.4 (d, ^3^*J*_C,C_ = 1.5 Hz, CH_3_) ppm.

### HPLC–ESI–MS and MS^2^

Analysis of extracts was performed on an HPLC-LC/MSD Ultra Trap System XCT 6330 (Agilent Technologies) equipped with a Luna Omega Polar C18 (5 µm, 150 × 2.1 mm; Phenomenex) column. Elution was performed with 0.1% formic acid (solvent A) and acetonitrile containing 0.06% formic acid (solvent B) at a flow rate of 0.4 ml/min using the following gradient: 0 to 100% solvent B over 20 min followed by an isocratic step of 100% B for 3 min. UV spectra were recorded between 230 and 600 nm by diode array detector. Mass spectrometry was performed using positive electrospray ionization (electrospray voltage 3.5 kV, heated capillary temperature 350° C, acquired mass range from 100 to 2200 m*/z*).

### In-silico analysis of liu cluster distribution in *Actinobacteria*

A database containing all 31.598 assembled *Actinobacteria* genomes listed in the Genome Taxonomy Database (GTDB; last checked 4th August 2022) was created [80]. The cblaster search module (default settings) was used to analyze genomes from this database using query sequences ranging from *sco2775-sco2779* or *sco2774*-*sco2779* from the genome of *S. coelicolor* M1154 [81].

## Supplementary Information

**Additional file 1: Figure S1. **Degradation of leucine and isovalerate via the Liu-pathway as described for *P. aeruginosa *PAO1 [29]. **Figure S2. **Genetic organization of the caprazamycin biosynthetic gene cluster. Genes putatively involved in colour coding according to McErlean et al. [17]. **Figure S3. **Extracted ion chromatograms of *S. coelicolor *M1154 (three individual mutants). Masses are shown for caprazamycin aglycons E/F with *m/z *of 930.5, caprazamycin aglycons C/D/G with *m/z *of 944.5, caprazamycin aglycons A/B with *m/z *of 958.5 and the respective hydroxyacylcaprazols E/F with *m/z *of 802.5, hydroxyacylcaprazols C/D/G with *m/z of *816.5 and hydroxyacylcaprazols A/B with *m/z *of 830.5. **Figure S4. **Extracted ion chromatograms of *S. coelicolor *M1154/cpzLK09 (three individual mutants). Masses are shown for caprazamycin aglycons E/F with *m/z *of 930.5, caprazamycin aglycons C/D/G with *m/z *of 944.5, caprazamycin aglycons A/B with *m/z *of 958.5 and the respective hydroxyacylcaprazols E/F with *m/z *of 802.5, hydroxyacylcaprazols C/D/G with *m/z *of 816.5 and hydroxyacylcaprazols A/B with *m/z *of 830.5. **Figure S5. **Extracted ion chromatograms of *S. coelicolor *M1154/cpzDB04 (three individual mutants). Masses are shown for caprazamycin aglycons E/F with *m/z *of 930.5, caprazamycin aglycons C/D/G with *m/z *of 944.5, caprazamycin aglycons A/B with *m/z *of 958.5 and the respective hydroxyacylcaprazols E/F with *m/z *of 802.5, hydroxyacylcaprazols C/D/G with *m/z *of 816.5 and hydroxyacylcaprazols A/B with *m/z *of 830.5. **Figure S6. **Genetic organization of clusters encoding for Liu-pathway from *P. aeruginosa *PAO1, caprazamycin wildtype producer *S. sp. *MK730-62F2 and the heterologous caprazamycin producer *S. coelicolor *M1154. Additional genes in *Streptomyces *strains are shown transparent. Table shows genes from *P. aeruginosa *PAO1 and their proposed function along with homologue genes found in *S. sp. *MK730-62F2 and *S. coelicolor *M1154. Values in brackets indicate % identities/similarities (n.s. no significant similarities). **Figure S7. **Distribution of *liu *clusters in *Actinobacteria *and *Streptomyces*. **A**: Cblaster detected 4837 similar clusters containing at least three genes homolog to the query sequence *sco2775*-*sco2779 *from *S. coelicolor *M1154. We discovered that 1.431 out of 31.598 (4.5%) *Actinobacteria *assemblies and 1.344 out of 2.574 (52.2%) *Streptomyces *assemblies and listed in the GTDB contain a *liu *cluster with homologues of all five query genes. **B**: Cblaster detected 5009 similar clusters containing at least three genes homolog to the query sequence *sco2774*-*sco2779 *from *S. coelicolor *M1154. We discovered that 1.246 out of 31.598 (3.9%) *Actinobacteria *assemblies and 1.241 out of 2.574 (48.2%) *Streptomyces *assemblies and listed in the GTDB contain a *liu *cluster with homologues of all six query genes. A homologue of *sco2774 *could only be found in 1.376 out of 5009 (27,5%) of the *Actinobacteria *clusters detected by cblaster, whereas the majority of detected *Streptomyces *cluster contain this gene with 1.343 out of 1.652 sequences (81,3%). **Figure S8. **Extracted ion chromatograms of *S. coelicolor *M1154Δ*sco2776-sco2779*/cpzLK09 (three individual mutants). Masses are shown for caprazamycin aglycons E/F with *m/z *of 930.5, caprazamycin aglycons C/D/G with *m/z *of 944.5, caprazamycin aglycons A/B with *m/z *of 958.5 and the respective hydroxyacylcaprazols E/F with *m/z *of 802.5, hydroxyacylcaprazols C/D/G with *m/z of *816.5 and hydroxyacylcaprazols A/B with *m/z *of 830.5. **Figure S9. **Extracted ion chromatograms of *S. coelicolor *M1154Δ*sco2776-sco2779*/cpzDB04 (three individual mutants). Masses are shown for caprazamycin aglycons E/F with *m/z *of 930.5, caprazamycin aglycons C/D/G with *m/z *of 944.5, caprazamycin aglycons A/B with *m/z *of 958.5 and the respective hydroxyacylcaprazols E/F with *m/z *of 802.5, hydroxyacylcaprazols C/D/G with *m/z of *816.5 and hydroxyacylcaprazols A/B with *m/z *of 830.5. **Figure S10. **Total ion chromatograms of *S. coelicolor *M1154Δ*sco2776-sco2779*/cpzDB04 and *S. coelicolor *M1154Δ*sco2776-sco2779*/cpzLK09. MS^2^-fragmentation patterns of peak 1 (hydoxyacylcaprazol E, R_t_ 12.9 min) and peak 2 (caprazamycin aglycon E, R_t_ 13.8 min) are shown together with corresponding fragmentation schemes. **Figure S11. **Extracted ion chromatograms of *S. coelicolor* M1154Δ*sco2779*/cpzLK09 (three individual mutants). Masses are shown for caprazamycin aglycons E/F with *m/z *of 930.5, caprazamycin aglycons C/D/G with *m/z *of 944.5, caprazamycin aglycons A/B with *m/z *of 958.5 and the respective hydroxyacylcaprazols E/F with *m/z *of 802.5, hydroxyacylcaprazols C/D/G with *m/z of *816.5 and hydroxyacylcaprazols A/B with *m/z *of 830.5. **Figure S12. **Extracted ion chromatograms of *S. coelicolor* M1154Δ*sco2779*/cpzDB04 (three individual mutants). Masses are shown for caprazamycin aglycons E/F with *m/z *of 930.5, caprazamycin aglycons C/D/G with *m/z *of 944.5, caprazamycin aglycons A/B with *m/z *of 958.5 and the respective hydroxyacylcaprazols E/F with *m/z *of 802.5, hydroxyacylcaprazols C/D/G with *m/z of *816.5 and hydroxyacylcaprazols A/B with *m/z *of 830.5. **Figure S13. **Extracted ion chromatograms of *S. coelicolor* M1154Δ*sco2776*/cpzLK09 (three individual mutants). Masses are shown for caprazamycin aglycons E/F with *m/z *of 930.5, caprazamycin aglycons C/D/G with *m/z *of 944.5, caprazamycin aglycons A/B with *m/z *of 958.5 and the respective hydroxyacylcaprazols E/F with *m/z *of 802.5, hydroxyacylcaprazols C/D/G with *m/z of *816.5 and hydroxyacylcaprazols A/B with *m/z *of 830.5. **Figure S14. **Extracted ion chromatograms of *S. coelicolor* M1154Δ*sco2776*/cpzDB04 (three individual mutants). Masses are shown for caprazamycin aglycons E/F with *m/z *of 930.5, caprazamycin aglycons C/D/G with *m/z *of 944.5, caprazamycin aglycons A/B with *m/z *of 958.5 and the respective hydroxyacylcaprazols E/F with *m/z *of 802.5, hydroxyacylcaprazols C/D/G with *m/z of *816.5 and hydroxyacylcaprazols A/B with *m/z *

## Data Availability

The data generated and/or analyzed during this study is included in this article and Additional file 1. The constructed mutant strains, cosmids and plasmids are available at the Department of Pharmaceutical Biology of the University of Tübingen, Tübingen, Germany.
