# Supplementary material for: Origin of the 3-methylglutaryl moiety in caprazamycin biosynthesis
Source: Microb Cell Fact. 2022 Nov 5;21:232. doi: 10.1186/s12934-022-01955-6 (PMC9636800; doi:10.1186/s12934-022-01955-6)
Supplement: Supplementary file 1 — Additional file 1: Figure S1. Degradation of leucine and isovalerate via the Liu-pathway as described for P. aeruginosa PAO1 [29]. Figure S2. Genetic organization of the caprazamycin biosynthetic gene cluster. Genes putatively involved in colour coding according to McErlean et al. [17]. Figure S3. Extracted ion chromatograms of S. coelicolor M1154 (three individual mutants). Masses are shown for caprazamycin aglycons E/F with m/z of 930.5, caprazamycin aglycons C/D/G with m/z of 944.5, caprazamycin aglycons A/B with m/z of 958.5 and the respective hydroxyacylcaprazols E/F with m/z of 802.5, hydroxyacylcaprazols C/D/G with m/z of 816.5 and hydroxyacylcaprazols A/B with m/z of 830.5. Figure S4. Extracted ion chromatograms of S. coelicolor M1154/cpzLK09 (three individual mutants). Masses are shown for caprazamycin aglycons E/F with m/z of 930.5, caprazamycin aglycons C/D/G with m/z of 944.5, caprazamycin aglycons A/B with m/z of 958.5 and the respective hydroxyacylcaprazols E/F with m/z of 802.5, hydroxyacylcaprazols C/D/G with m/z of 816.5 and hydroxyacylcaprazols A/B with m/z of 830.5. Figure S5. Extracted ion chromatograms of S. coelicolor M1154/cpzDB04 (three individual mutants). Masses are shown for caprazamycin aglycons E/F with m/z of 930.5, caprazamycin aglycons C/D/G with m/z of 944.5, caprazamycin aglycons A/B with m/z of 958.5 and the respective hydroxyacylcaprazols E/F with m/z of 802.5, hydroxyacylcaprazols C/D/G with m/z of 816.5 and hydroxyacylcaprazols A/B with m/z of 830.5. Figure S6. Genetic organization of clusters encoding for Liu-pathway from P. aeruginosa PAO1, caprazamycin wildtype producer S. sp. MK730-62F2 and the heterologous caprazamycin producer S. coelicolor M1154. Additional genes in Streptomyces strains are shown transparent. Table shows genes from P. aeruginosa PAO1 and their proposed function along with homologue genes found in S. sp. MK730-62F2 and S. coelicolor M1154. Values in brackets indicate % identities/similarities (n.s. no sign [file 12934_2022_1955_MOESM1_ESM.pdf]

Additional file 1 for

Origin of the 3-methylglutaryl moiety in Caprazamycin biosynthesis

Daniel Bär[1], Benjamin Konetschny[1], Andreas Kulik[2], Houchao Xu[3], Davide Paccagnella[4], Patrick Beller[1], Nadine Ziemert[4,5], Jeroen S. Dickschat[3] and Bertolt Gust[1]

[1]Department of Pharmaceutical Biology, Eberhard-Karls University Tübingen, Auf der Morgenstelle 8, 72076 Tübingen, Germany

[2]Department of Microbial Bioactive Compounds, Interfaculty Institute of Microbiology and Infection Medicine, Eberhard-Karls University Tübingen, Auf der Morgenstelle 28, 72076 Tübingen, Germany

[3]Kekulé-Institute for Organic Chemistry and Biochemistry, University of Bonn, Gerhard-Domagk-Straße 1, 53121 Bonn, Germany

[4]Interfaculty Institute of Microbiology and Infection Medicine, Institute for Bioinformatics and Medical Informatics, Eberhard-Karls University Tübingen, Auf der Morgenstelle 28, 72076 Tübingen, Germany

[5]German Center for Infection Research (DZIF), Partner Site Tübingen, Tübingen, Germany

Corresponding author: bertolt.gust@uni-tuebingen.de

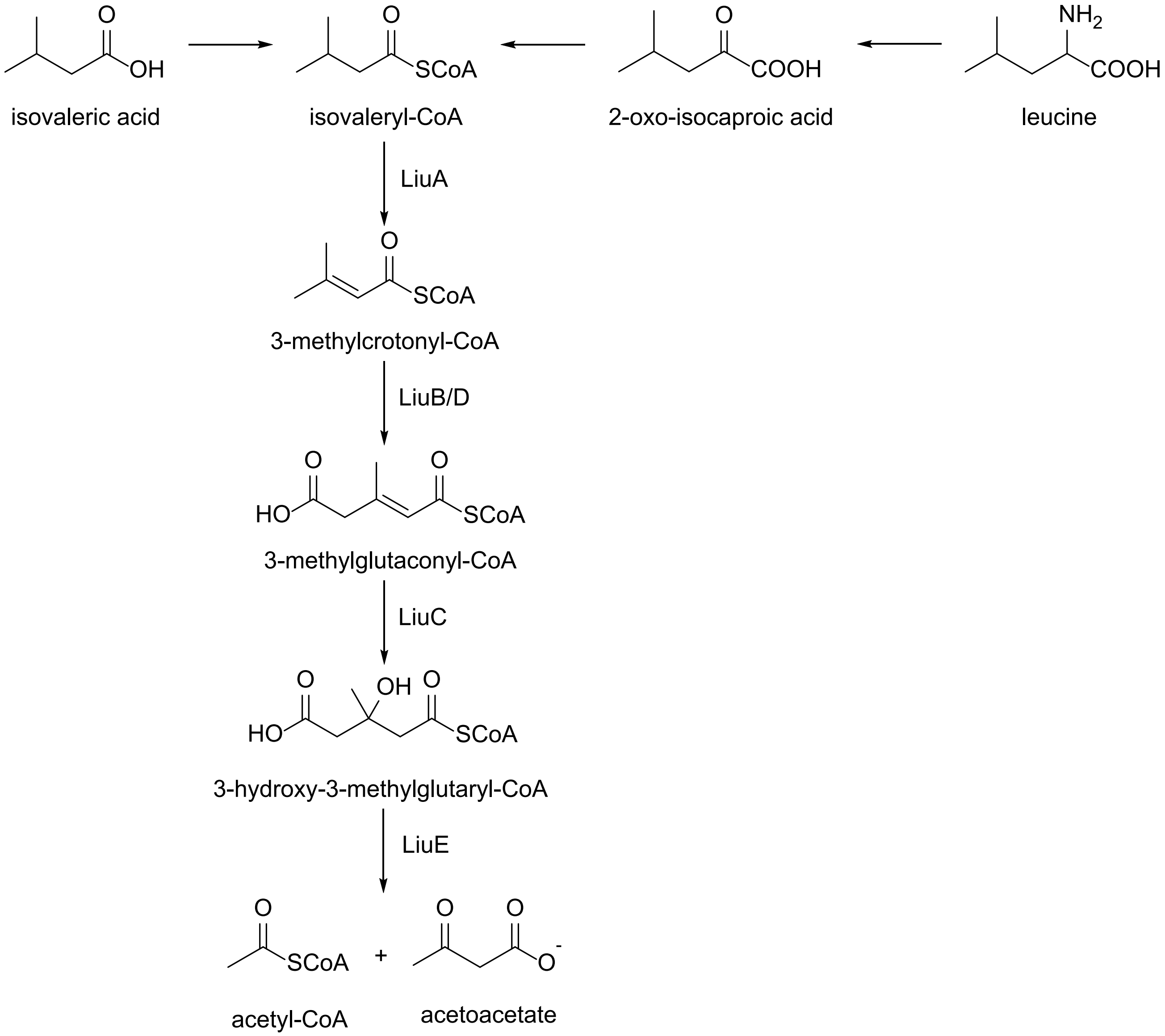

Figure S1: Degradation of leucine and isovalerate via the Liu-pathway as described for *P. aeruginosa* PAO1 (Chávez-Avilés *et al.* 2009).

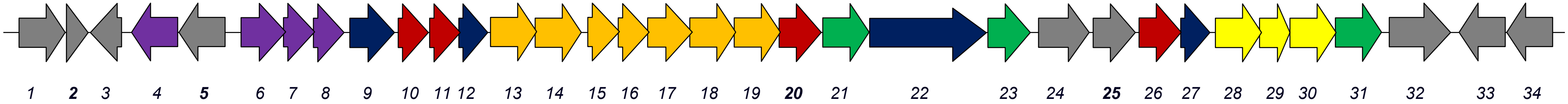

gene	***predicted function***
cpz2	acyl dehydratase
cpz5	3-hydroxy-3-methylglutaryl-CoA synthase
cpz20	acyl-CoA synthase
cpz25	dehydrogenase

nucleoside core
diazepanone/tailoring
sulfation
coupling
rhamnose/monosaccharide
resistance/regulation
unknown function

Figure S2: Genetic organization of the caprazamycin biosynthetic gene cluster. Genes putatively involved in 3-methylglutaryl-CoA supply written in bold. Function colour coding according to McErlean *et. al* 2021.

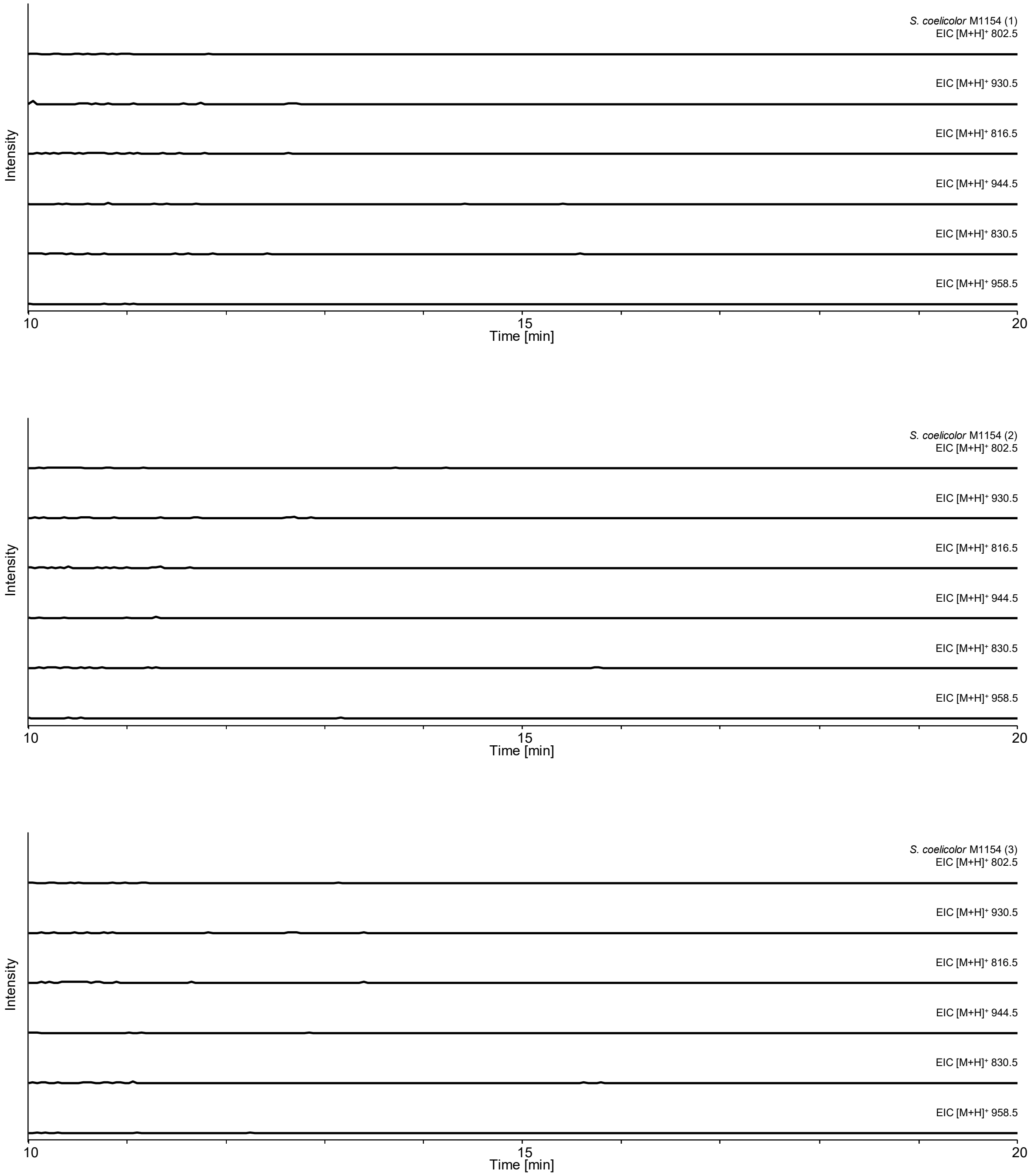

Figure S3: Extracted ion chromatograms of *S. coelicolor* M1154 (three individual mutants). Masses are shown for caprazamycin aglycons E/F with *m/z* of 930.5, caprazamycin aglycons C/D/G with *m/z* of 944.5, caprazamycin aglycons A/B with *m/z* of 958.5 and the respective hydroxyacylcaprazols E/F with *m/z* of 802.5, hydroxyacylcaprazols C/D/G with *m/z of* 816.5 and hydroxyacylcaprazols A/B with *m/z* of 830.5.

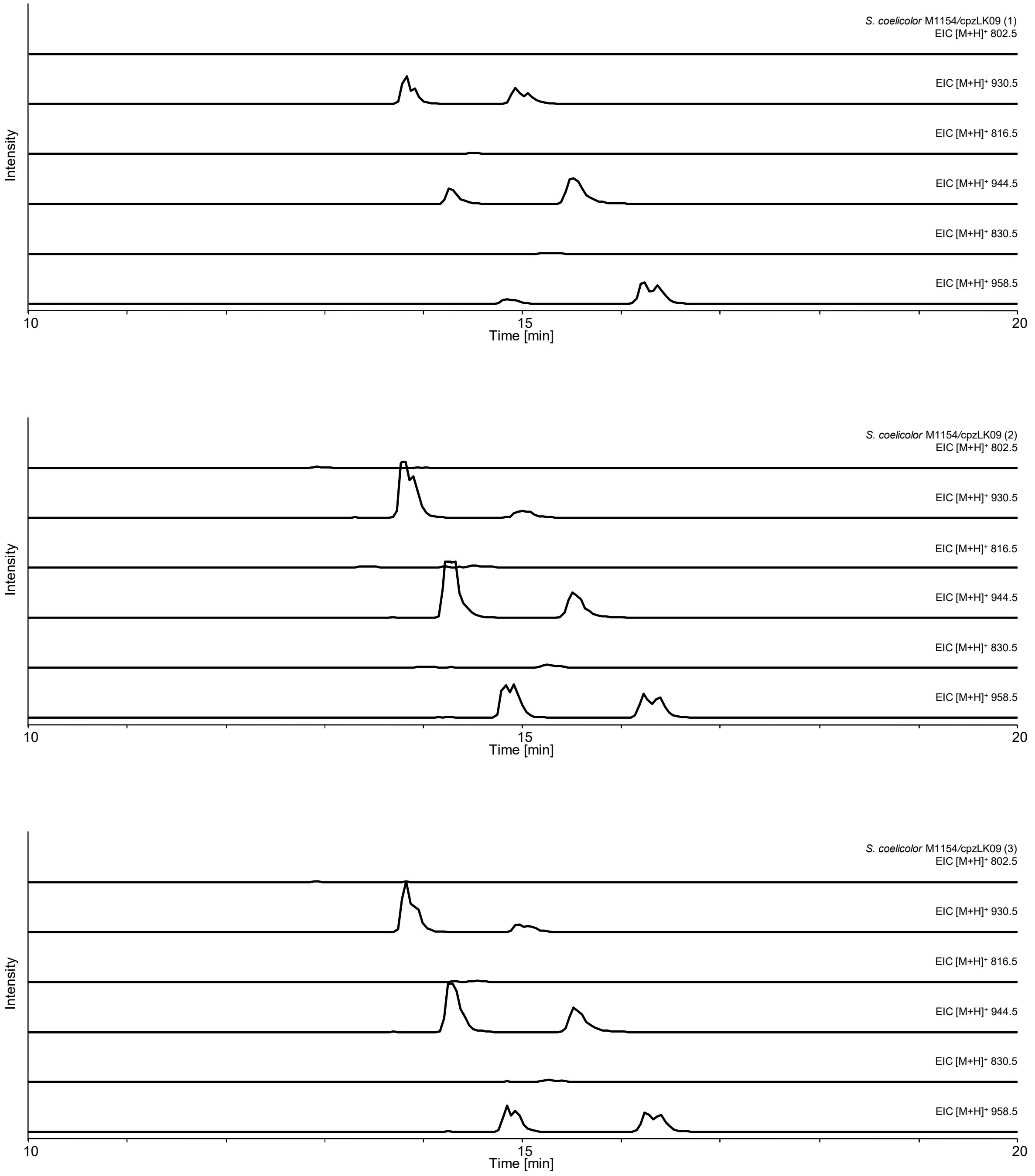

Figure S4: Extracted ion chromatograms of *S. coelicolor* M1154/cpzLK09 (three individual mutants). Masses are shown for caprazamycin aglycons E/F with *m/z* of 930.5, caprazamycin aglycons C/D/G with *m/z* of 944.5, caprazamycin aglycons A/B with *m/z* of 958.5 and the respective hydroxyacylcaprazols E/F with *m/z* of 802.5, hydroxyacylcaprazols C/D/G with *m/z* of 816.5 and hydroxyacylcaprazols A/B with *m/z* of 830.5.

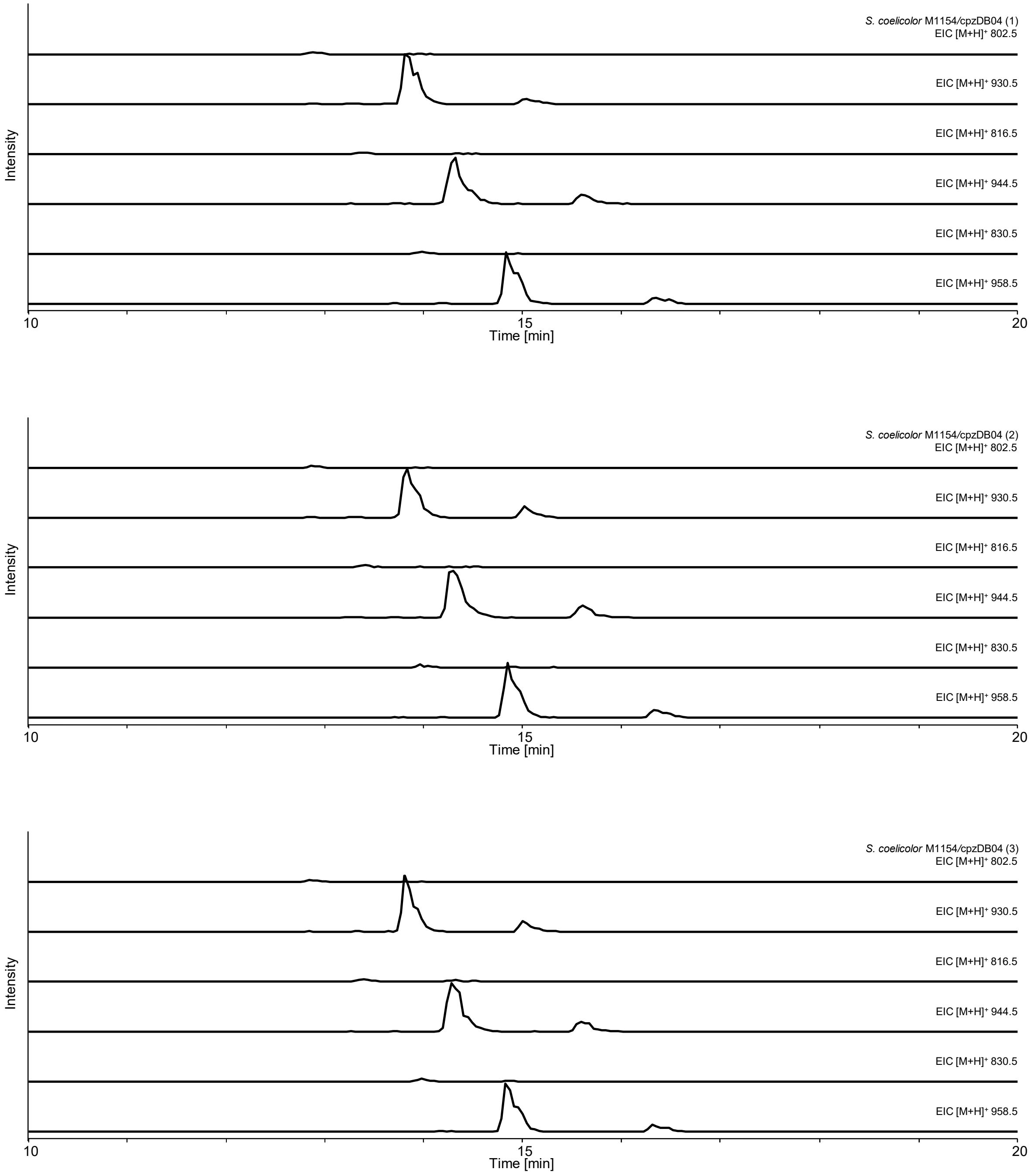

Figure S5: Extracted ion chromatograms of *S. coelicolor* M1154/cpzDB04 (three individual mutants). Masses are shown for caprazamycin aglycons E/F with *m/z* of 930.5, caprazamycin aglycons C/D/G with *m/z* of 944.5, caprazamycin aglycons A/B with *m/z* of 958.5 and the respective hydroxyacylcaprazols E/F with *m/z* of 802.5, hydroxyacylcaprazols C/D/G with *m/z* of 816.5 and hydroxyacylcaprazols A/B with *m/z* of 830.5.

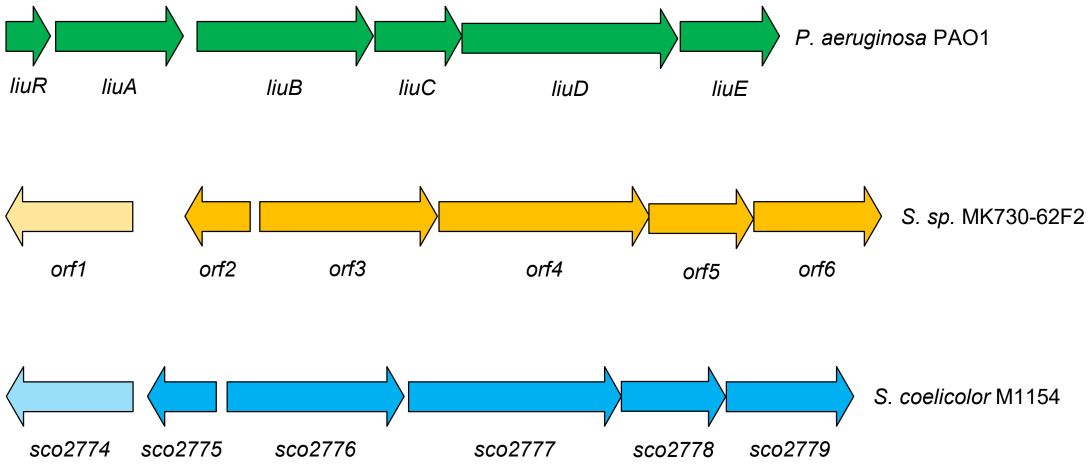

**proposed function**	***P. aeruginosa* PAO1**	***S. sp.* MK730-62F2**	***S. coelicolor* M1154**
transcriptional regulator	*liuR* / *PA2016*	*orf2* (n.s.)	*sco2775* (n.s.)
isovaleryl-CoA dehydrogenase	*liuA* / *PA2015*	*orf6* (37/53)	*sco2779* (37/53)
3-methylcrotonyl-CoA carboxylase subunit β	*liuB* / *PA2014*	*orf3* (67/77)	*sco2776* (68/78)
3-methylglutaconyl-CoA hydratase	*liuC* / *PA2013*	---	---
3-methylcrotonyl-CoA carboxylase subunit α	*liuD* / *PA2012*	*orf4* (49/64)	*sco2777* (50/63)
3-hydroxy-3-methylglutaryl-CoA lyase	*liuE* / *PA2011*	*orf5* (46/62)	*sco2778* (48/63)
acyl-CoA dehydrogenase	---	*orf1*	*sco2774*

Figure S6: Genetic organization of clusters encoding for Liu-pathway from *P. aeruginosa* PAO1, caprazamycin wildtype producer *S. sp.* MK730-62F2 and the heterologous caprazamycin producer *S. coelicolor* M1154. Additional genes in *Streptomyces* strains are shown transparent. Table shows genes from *P. aeruginosa* PAO1 and their proposed function along with homologue genes found in *S. sp.* MK730-62F2 and *S. coelicolor* M1154. Values in brackets indicate % identities/similarities (n.s. no significant similarities).

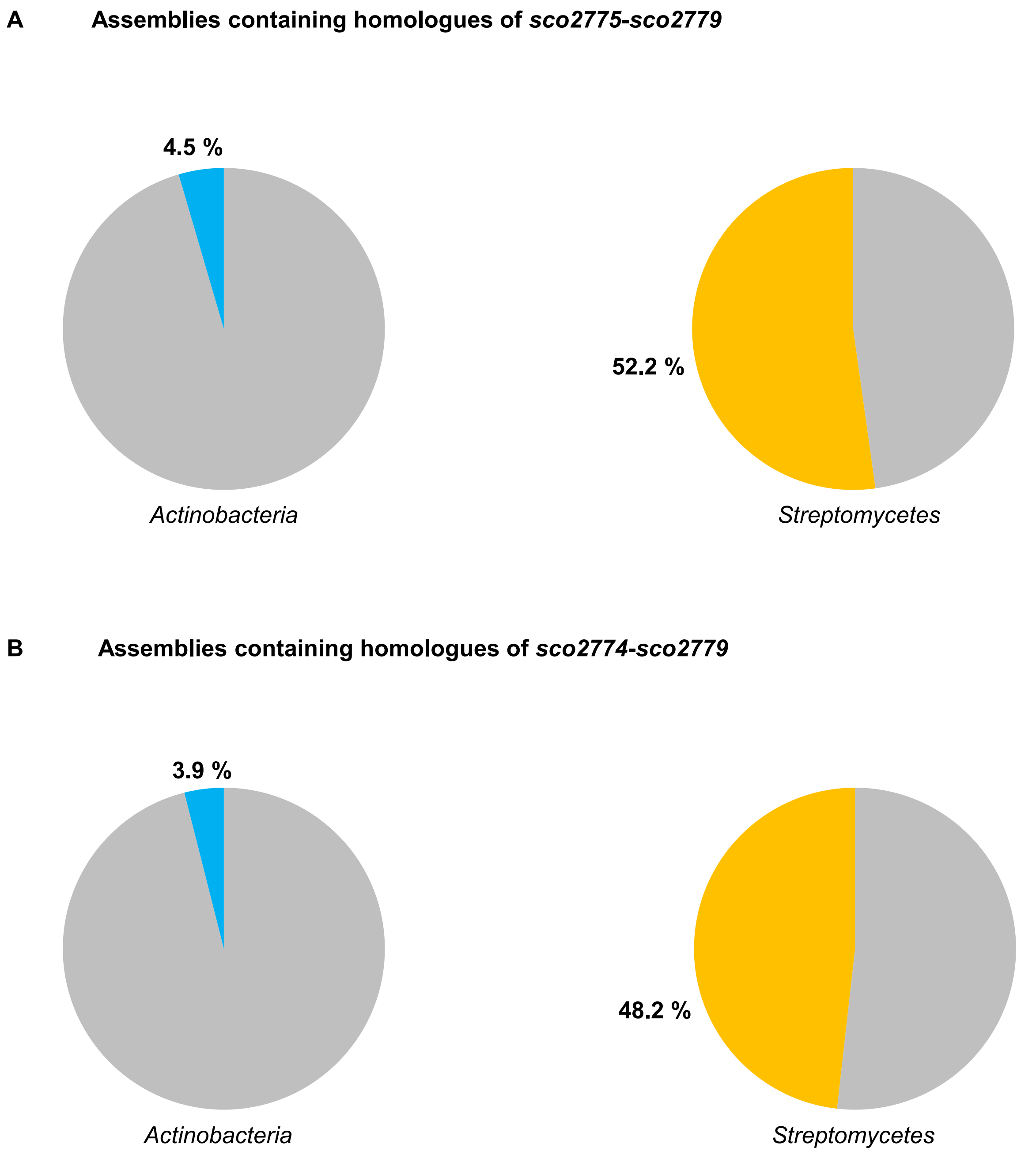

Figure S7: Distribution of *liu* clusters in *Actinobacteria* and *Streptomyces*. **A**: Cblaster detected 4837 similar clusters containing at least three genes homolog to the query sequence *sco2775*-*sco2779* from *S. coelicolor* M1154. We discovered that 1.431 out of 31.598 (4,5%) *Actinobacteria* assemblies and 1.344 out of 2.574 (52,2%) *Streptomyces* assemblies and listed in the GTDB contain a *liu* cluster with homologues of all five query genes. **B**: Cblaster detected 5009 similar clusters containing at least three genes homolog to the query sequence *sco2774*-*sco2779* from *S. coelicolor* M1154. We discovered that 1.246 out of 31.598 (3,9%) *Actinobacteria* assemblies and 1.241 out of 2.574 (48,2%) *Streptomyces* assemblies and listed in the GTDB contain a *liu* cluster with homologues of all six query genes. A homologue of *sco2774* could only be found in 1.376 out of 5009 (27,5%) of the *Actinobacteria* clusters detected by cblaster, whereas the majority of detected *Streptomyces* cluster contain this gene with 1.343 out of 1.652 sequences (81,3%).

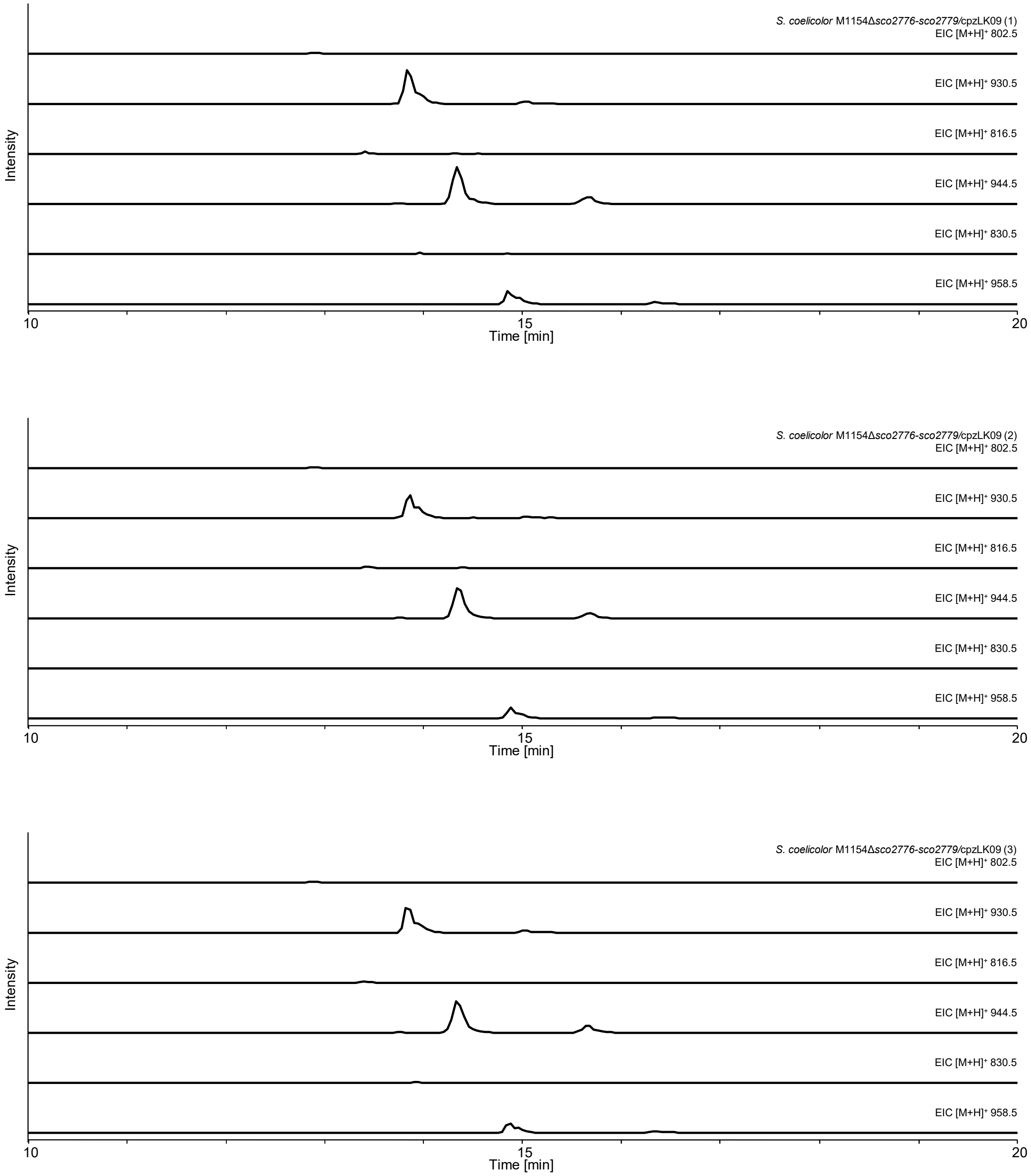

Figure S8: Extracted ion chromatograms of *S. coelicolor* M1154Δ*sco2776-sco2779*/cpzLK09 (three individual mutants). Masses are shown for caprazamycin aglycons E/F with *m/z* of 930.5, caprazamycin aglycons C/D/G with *m/z* of 944.5, caprazamycin aglycons A/B with *m/z* of 958.5 and the respective hydroxyacylcaprazols E/F with *m/z* of 802.5, hydroxyacylcaprazols C/D/G with *m/z of* 816.5 and hydroxyacylcaprazols A/B with *m/z* of 830.5.

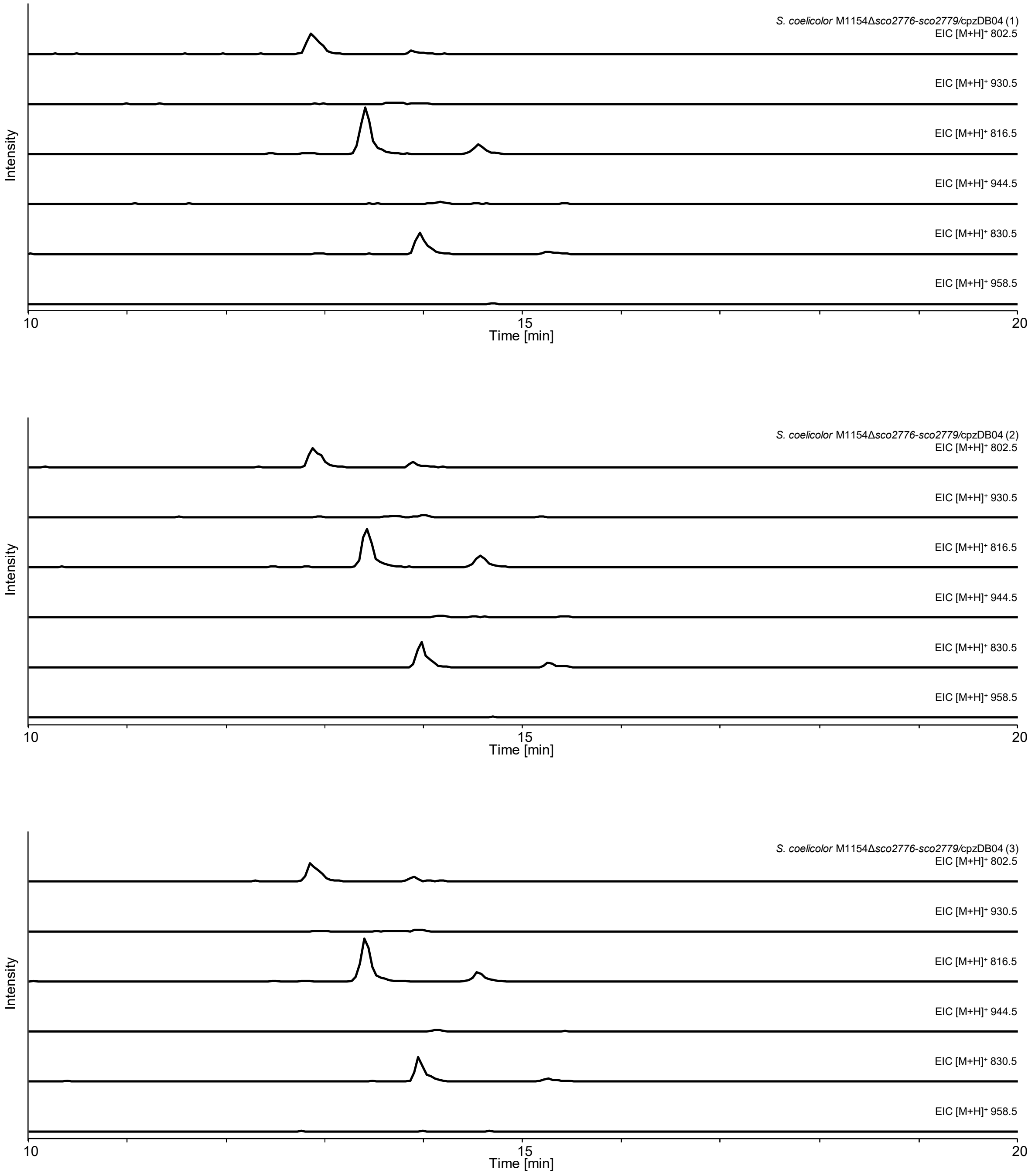

Figure S9: Extracted ion chromatograms of *S. coelicolor* M1154Δ*sco2776-sco2779*/cpzDB04 (three individual mutants). Masses are shown for caprazamycin aglycons E/F with *m/z* of 930.5, caprazamycin aglycons C/D/G with *m/z* of 944.5, caprazamycin aglycons A/B with *m/z* of 958.5 and the respective hydroxyacylcaprazols E/F with *m/z* of 802.5, hydroxyacylcaprazols C/D/G with *m/z of* 816.5 and hydroxyacylcaprazols A/B with *m/z* of 830.5.

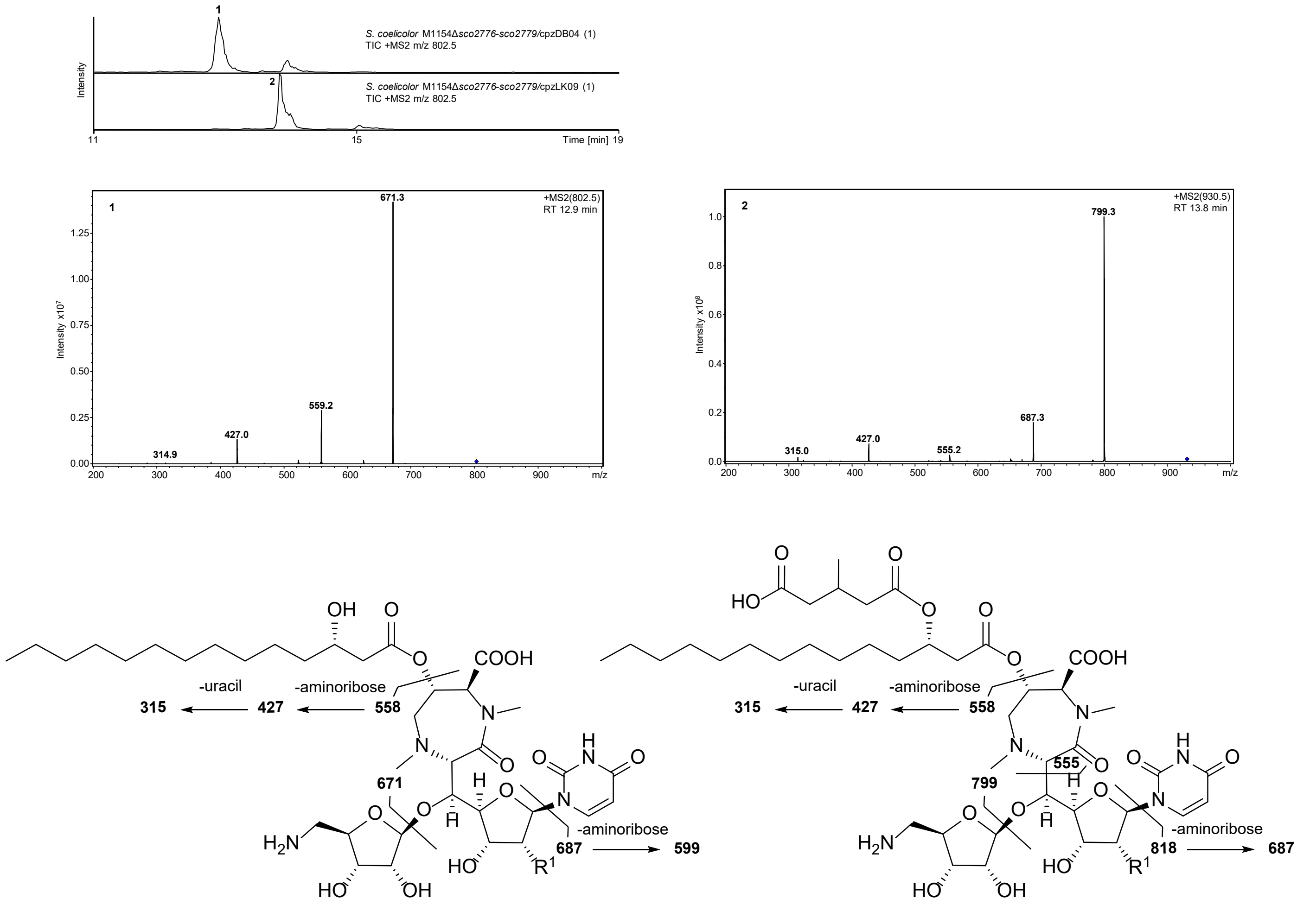

Hydroxyacylcaprazol E

Caprazamycin aglycon E

Figure S10: Total ion chromatograms of *S. coelicolor* M1154Δ*sco2776-sco2779*/cpzDB04 and *S. coelicolor* M1154Δ*sco2776-sco2779*/cpzLK09. MS^2-fragmentation patterns of peak 1 (hydoxyacylcaprazol E, R_t 12.9 min) and peak 2 (caprazamycin aglycon E, R_t 13.8 min) are shown together with corresponding fragmentation schemes.

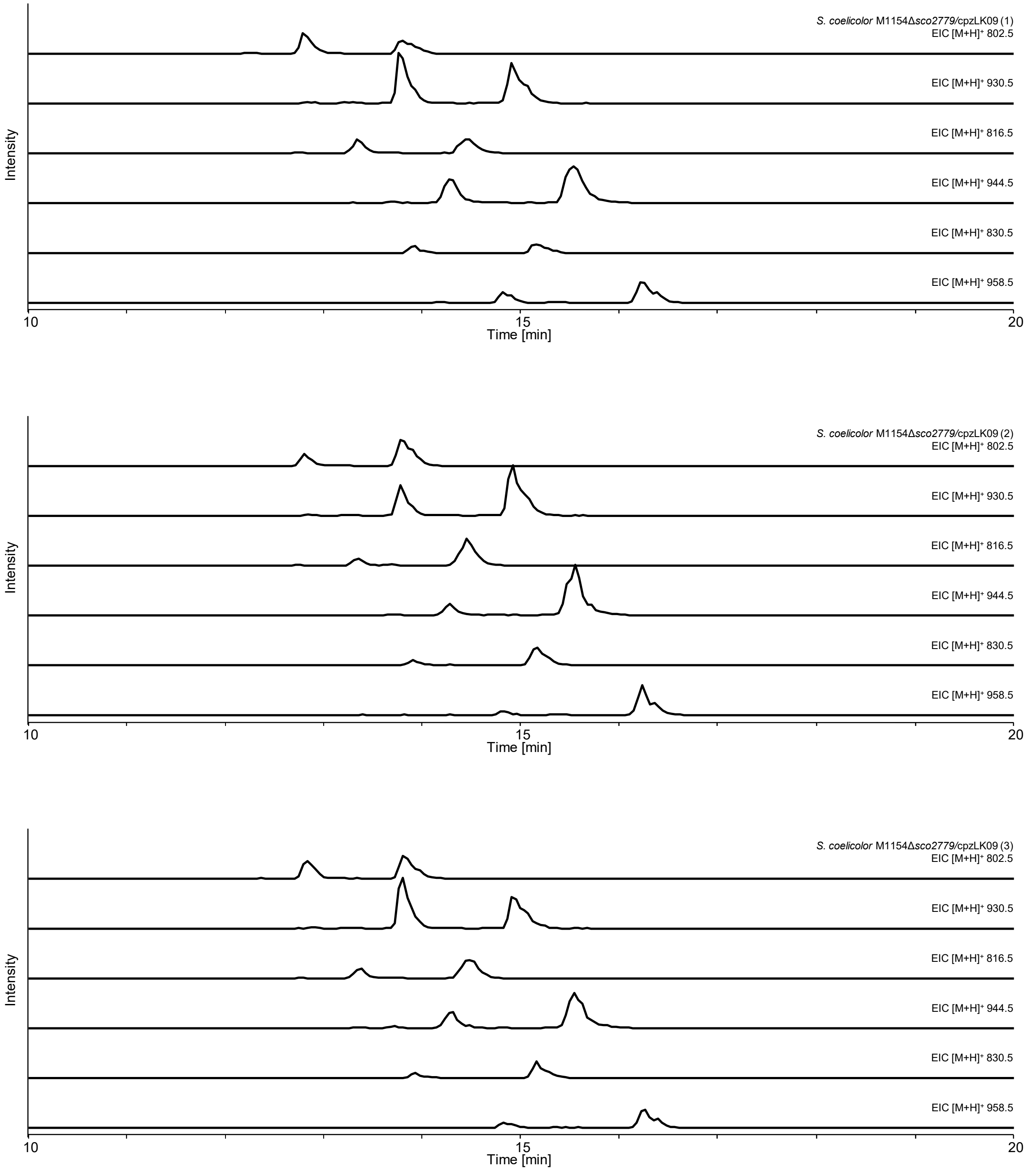

Figure S11: Extracted ion chromatograms of *S. coelicolor* M1154Δ*sco2779*/cpzLK09 (three individual mutants). Masses are shown for caprazamycin aglycons E/F with *m/z* of 930.5, caprazamycin aglycons C/D/G with *m/z* of 944.5, caprazamycin aglycons A/B with *m/z* of 958.5 and the respective hydroxyacylcaprazols E/F with *m/z* of 802.5, hydroxyacylcaprazols C/D/G with *m/z of* 816.5 and hydroxyacylcaprazols A/B with *m/z* of 830.5.

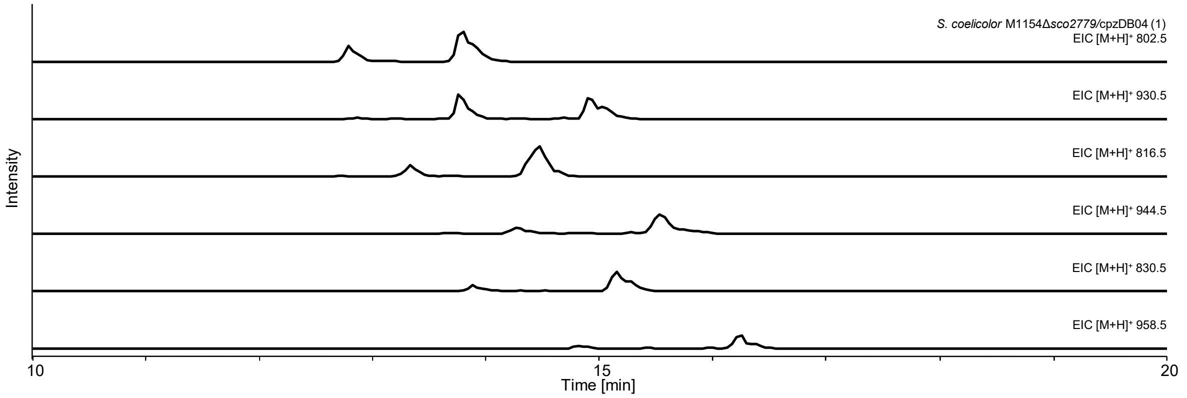

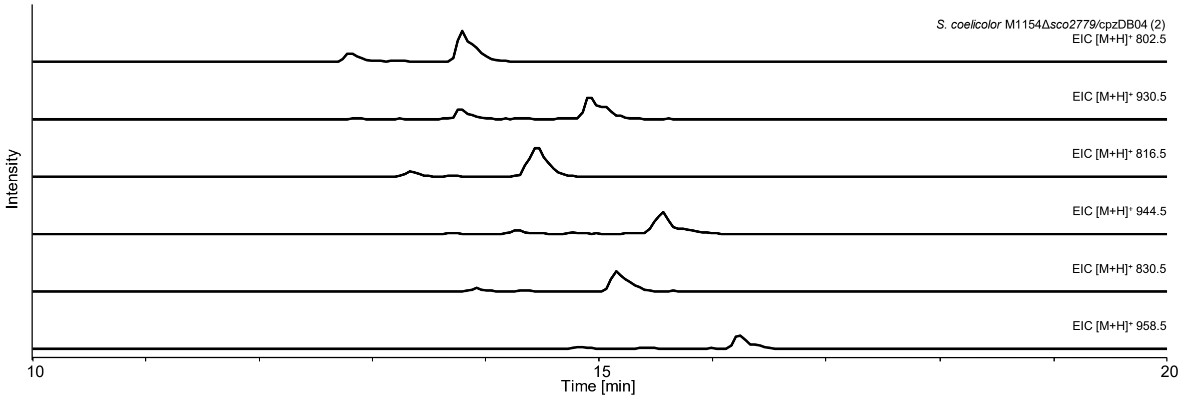

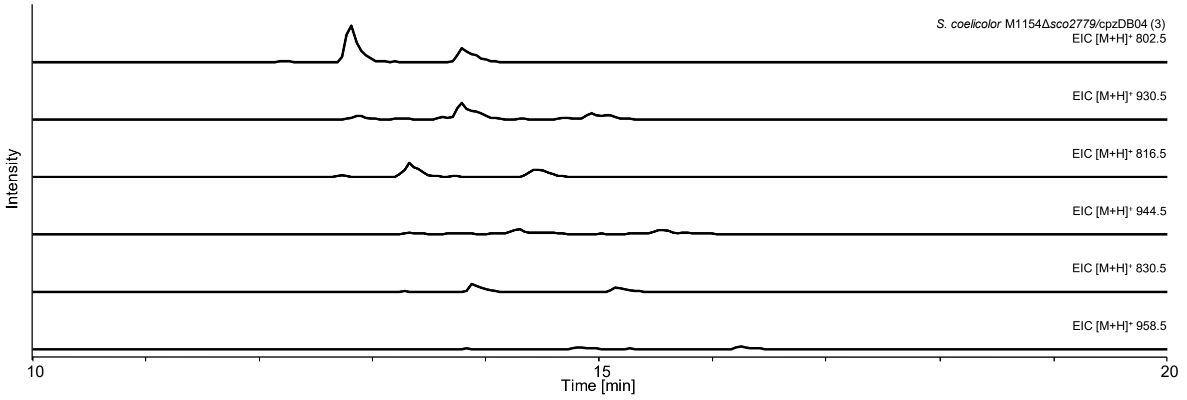

Figure S12: Extracted ion chromatograms of *S. coelicolor* M1154Δ*sco2779*/cpzDB04 (three individual mutants). Masses are shown for caprazamycin aglycons E/F with *m/z* of 930.5, caprazamycin aglycons C/D/G with *m/z* of 944.5, caprazamycin aglycons A/B with *m/z* of 958.5 and the respective hydroxyacylcaprazols E/F with *m/z* of 802.5, hydroxyacylcaprazols C/D/G with *m/z of* 816.5 and hydroxyacylcaprazols A/B with *m/z* of 830.5.

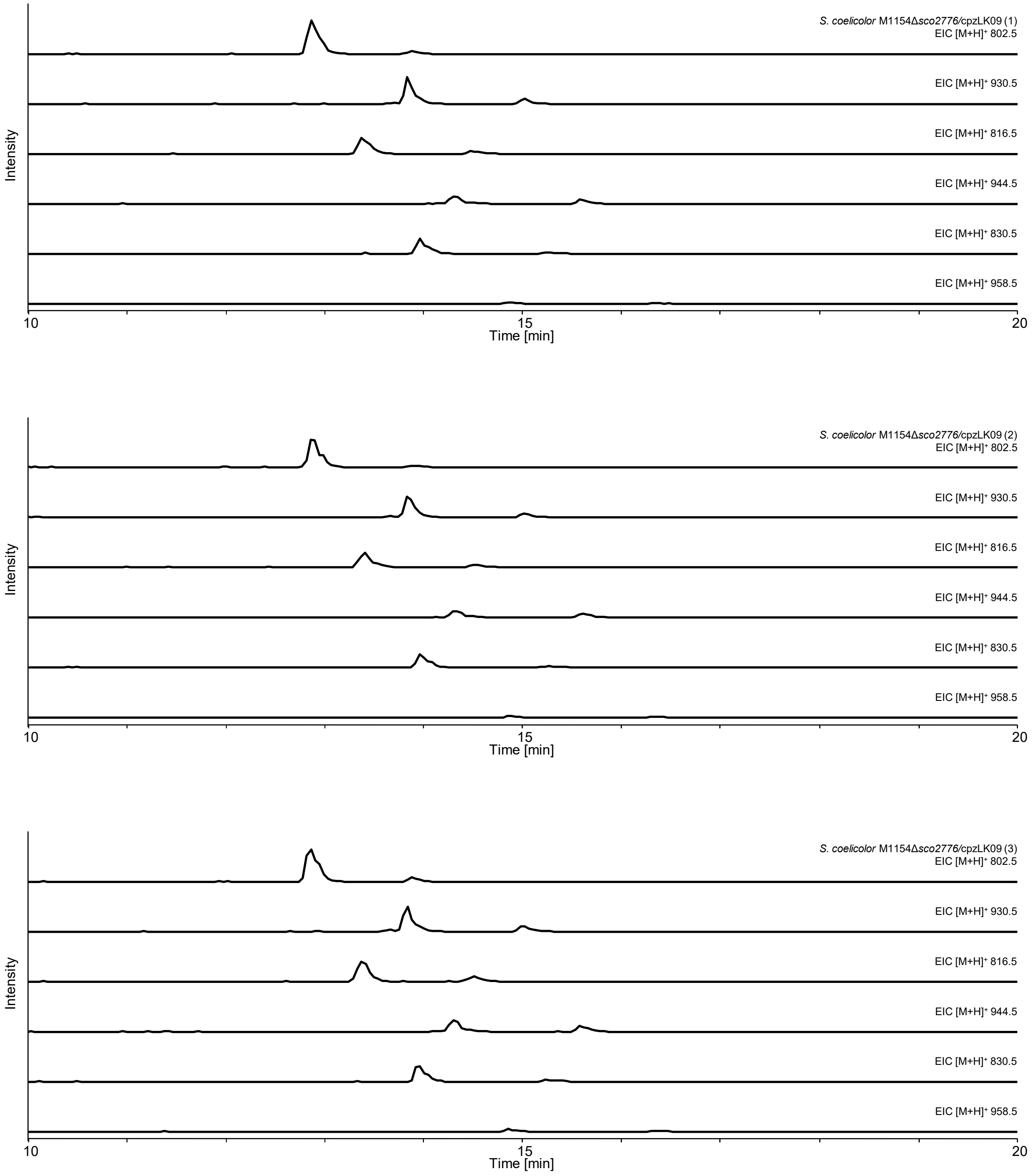

Figure S13: Extracted ion chromatograms of *S. coelicolor* M1154Δ*sco2776*/cpzLK09 (three individual mutants). Masses are shown for caprazamycin aglycons E/F with *m/z* of 930.5, caprazamycin aglycons C/D/G with *m/z* of 944.5, caprazamycin aglycons A/B with *m/z* of 958.5 and the respective hydroxyacylcaprazols E/F with *m/z* of 802.5, hydroxyacylcaprazols C/D/G with *m/z of* 816.5 and hydroxyacylcaprazols A/B with *m/z* of 830.5.

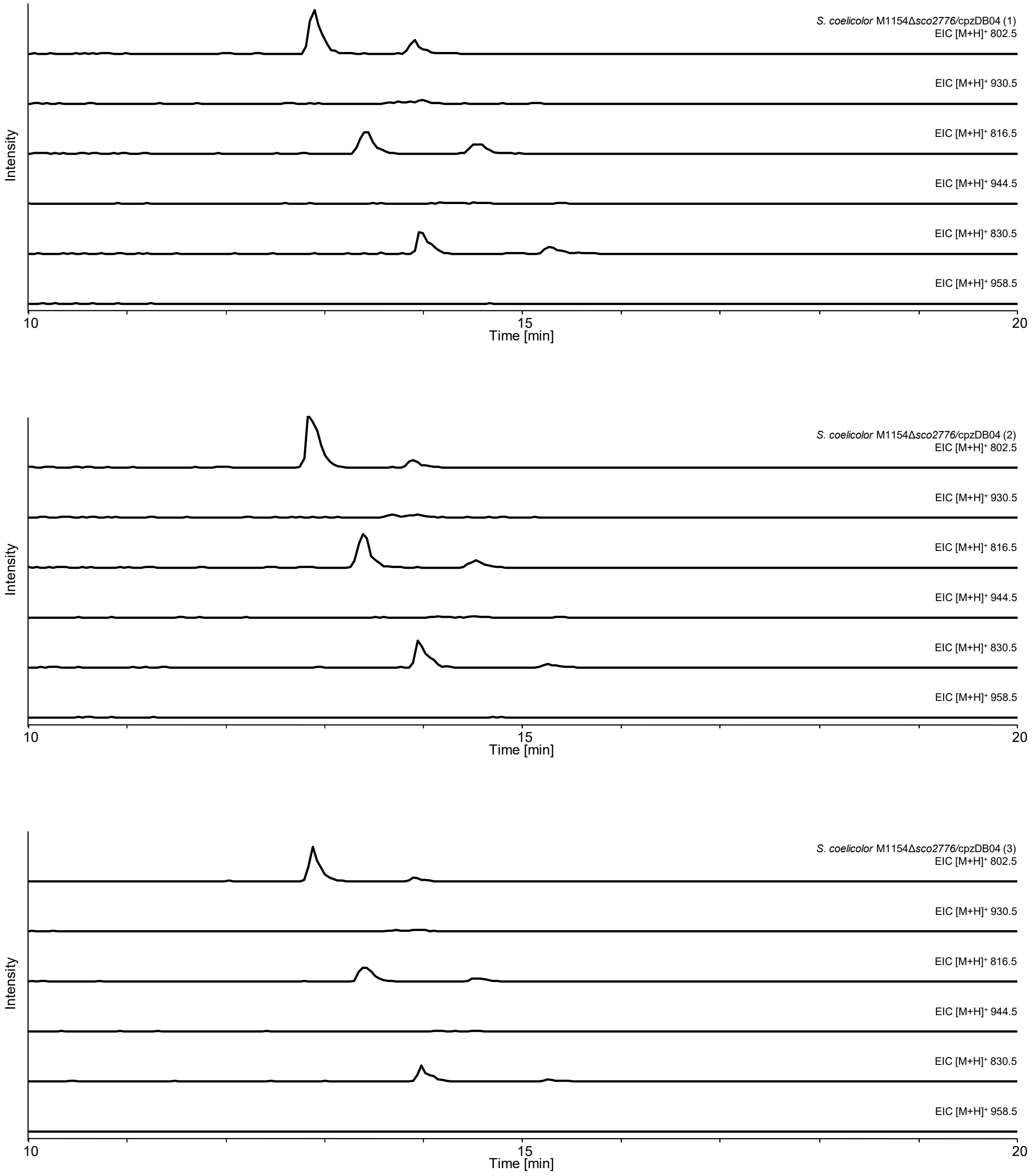

Figure S14: Extracted ion chromatograms of *S. coelicolor* M1154Δ*sco2776*/cpzDB04 (three individual mutants). Masses are shown for caprazamycin aglycons E/F with *m/z* of 930.5, caprazamycin aglycons C/D/G with *m/z* of 944.5, caprazamycin aglycons A/B with *m/z* of 958.5 and the respective hydroxyacylcaprazols E/F with *m/z* of 802.5, hydroxyacylcaprazols C/D/G with *m/z of* 816.5 and hydroxyacylcaprazols A/B with *m/z* of 830.5.

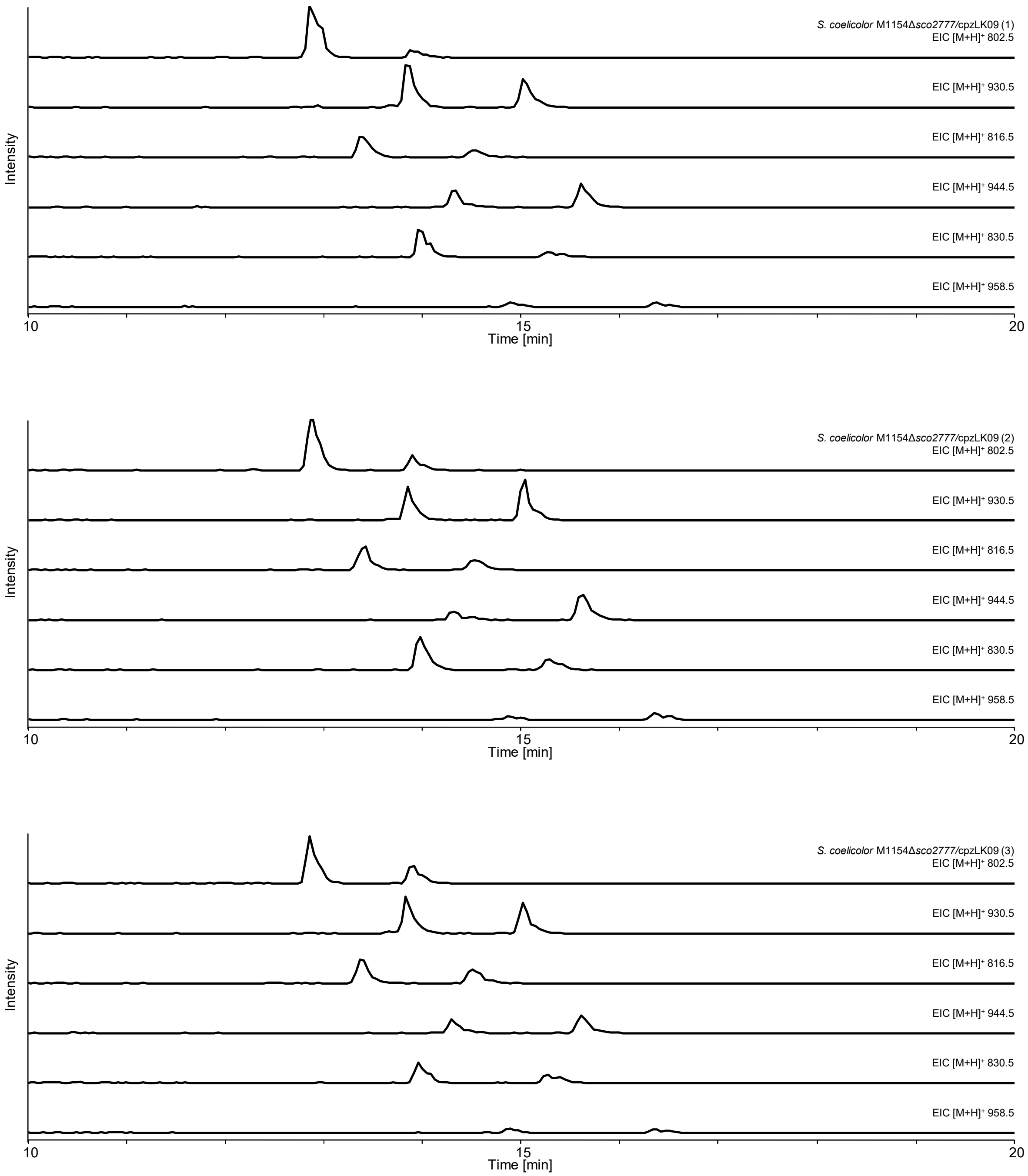

Figure S15: Extracted ion chromatograms of *S. coelicolor* M1154Δ*sco2777*/cpzLK09 (three individual mutants). Masses are shown for caprazamycin aglycons E/F with *m/z* of 930.5, caprazamycin aglycons C/D/G with *m/z* of 944.5, caprazamycin aglycons A/B with *m/z* of 958.5 and the respective hydroxyacylcaprazols E/F with *m/z* of 802.5, hydroxyacylcaprazols C/D/G with *m/z of* 816.5 and hydroxyacylcaprazols A/B with *m/z* of 830.5.

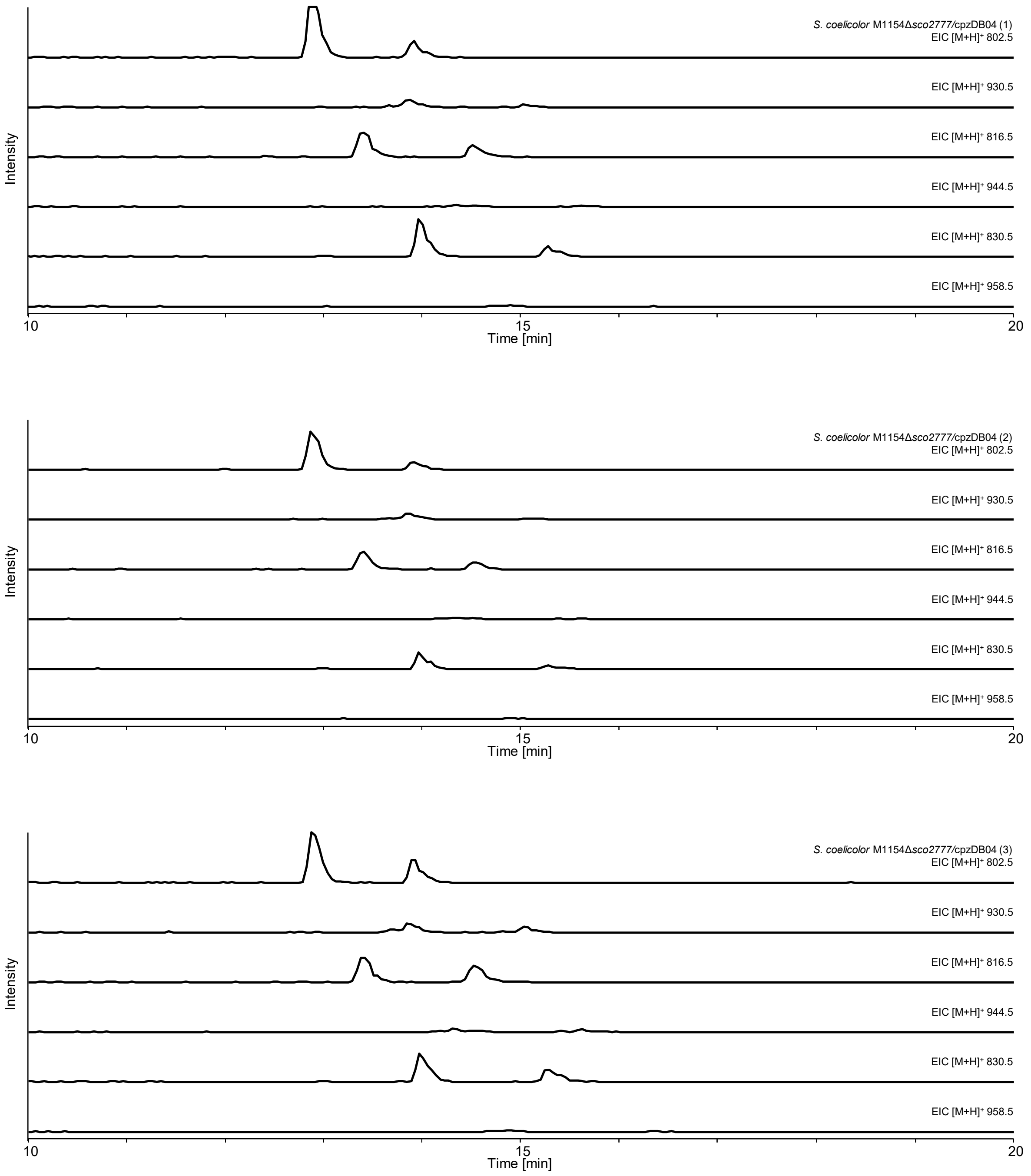

Figure S16: Extracted ion chromatograms of *S. coelicolor* M1154Δ*sco2777*/cpzDB04 (three individual mutants). Masses are shown for caprazamycin aglycons E/F with *m/z* of 930.5, caprazamycin aglycons C/D/G with *m/z* of 944.5, caprazamycin aglycons A/B with *m/z* of 958.5 and the respective hydroxyacylcaprazols E/F with *m/z* of 802.5, hydroxyacylcaprazols C/D/G with *m/z of* 816.5 and hydroxyacylcaprazols A/B with *m/z* of 830.5.

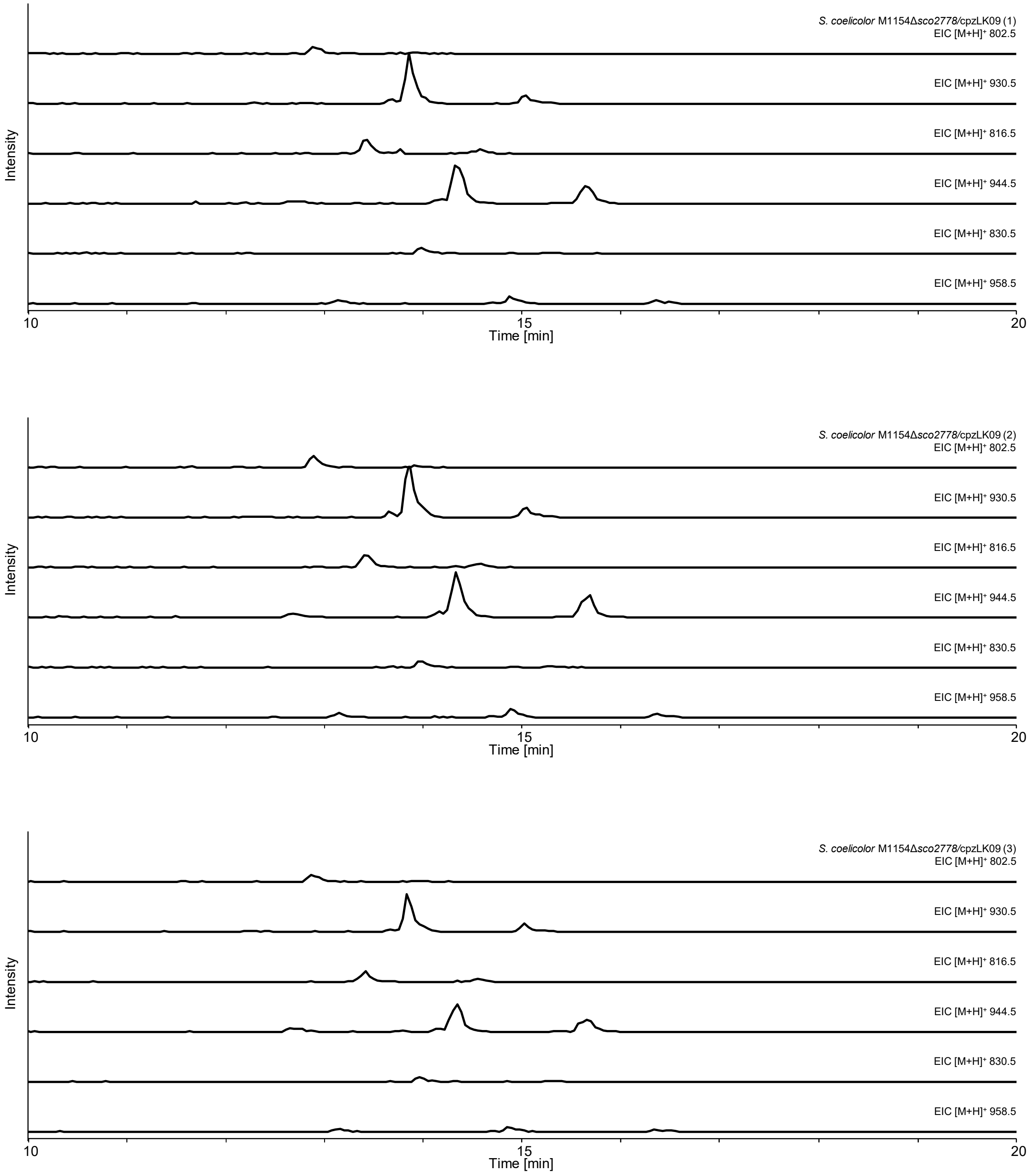

Figure S17: Extracted ion chromatograms of *S. coelicolor* M1154Δ*sco2778*/cpzLK09 (three individual mutants). Masses are shown for caprazamycin aglycons E/F with *m/z* of 930.5, caprazamycin aglycons C/D/G with *m/z* of 944.5, caprazamycin aglycons A/B with *m/z* of 958.5 and the respective hydroxyacylcaprazols E/F with *m/z* of 802.5, hydroxyacylcaprazols C/D/G with *m/z of* 816.5 and hydroxyacylcaprazols A/B with *m/z* of 830.5.

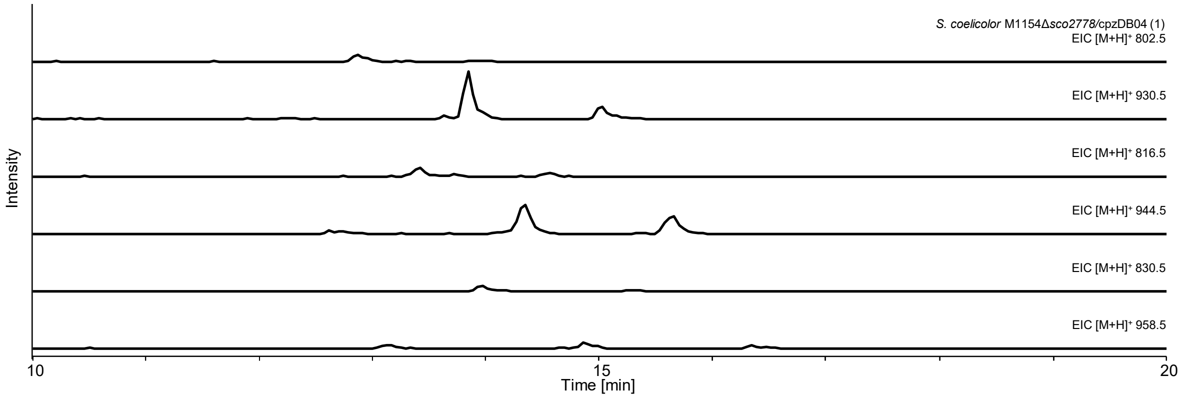

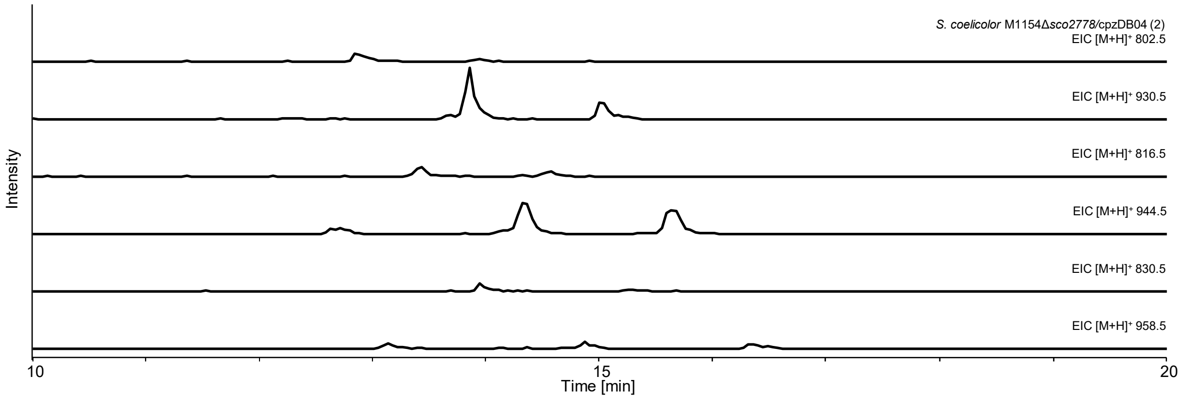

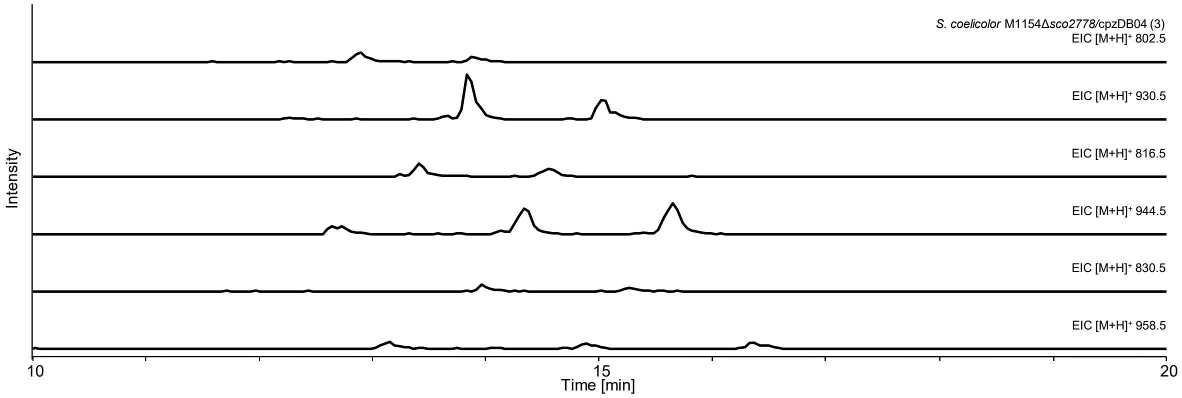

Figure S18: Extracted ion chromatograms of *S. coelicolor* M1154Δ*sco2778*/cpzDB04 (three individual mutants). Masses are shown for caprazamycin aglycons E/F with *m/z* of 930.5, caprazamycin aglycons C/D/G with *m/z* of 944.5, caprazamycin aglycons A/B with *m/z* of 958.5 and the respective hydroxyacylcaprazols E/F with *m/z* of 802.5, hydroxyacylcaprazols C/D/G with *m/z of* 816.5 and hydroxyacylcaprazols A/B with *m/z* of 830.5.

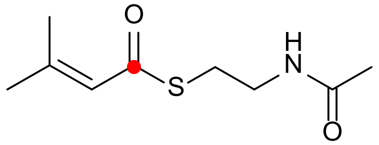

1-^{13}C-3-methylcrotonyl-SNAc

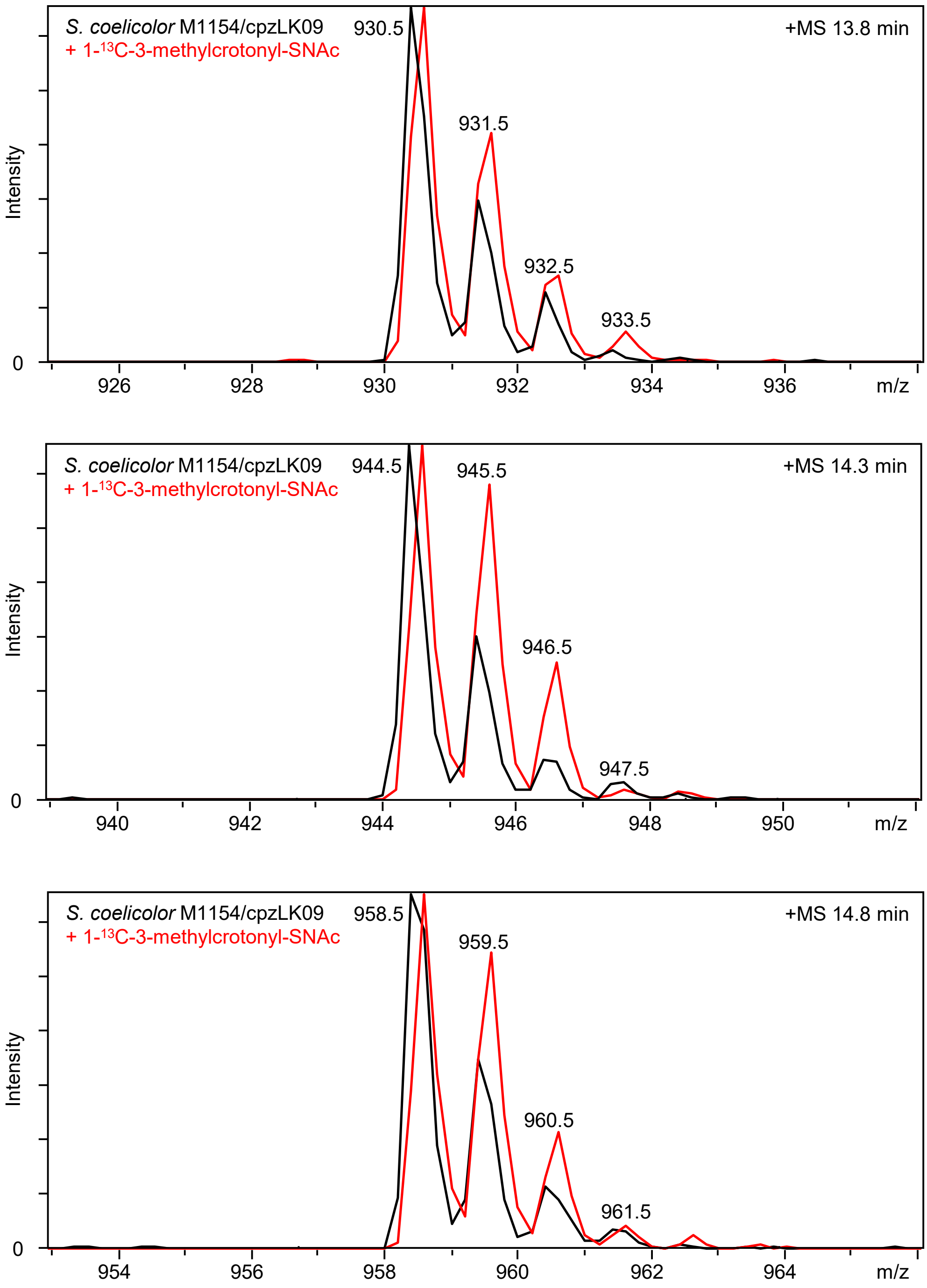

Figure S19: Chemical structure of 1-^{13}C-3-methylcrotonyl-SNAc. Mass spectra of an extract of *S. coelicolor* M1154/cpzLK09 without (black) and with 1-^{13}C-3-methylcrotonyl-SNAc added to the cultivation medium (red). Mass ranges are shown for isotopes of caprazamycin aglycons E/F with *m/z of* 930.5, caprazamycin aglycons C/D/G with *m/z of* 944.5 and caprazamycin aglycons A/B with *m/z of* 958.5.

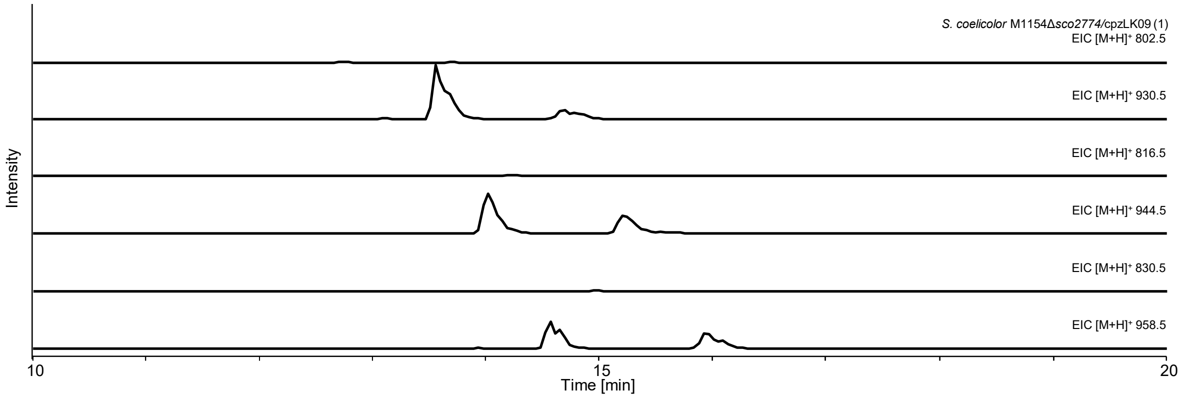

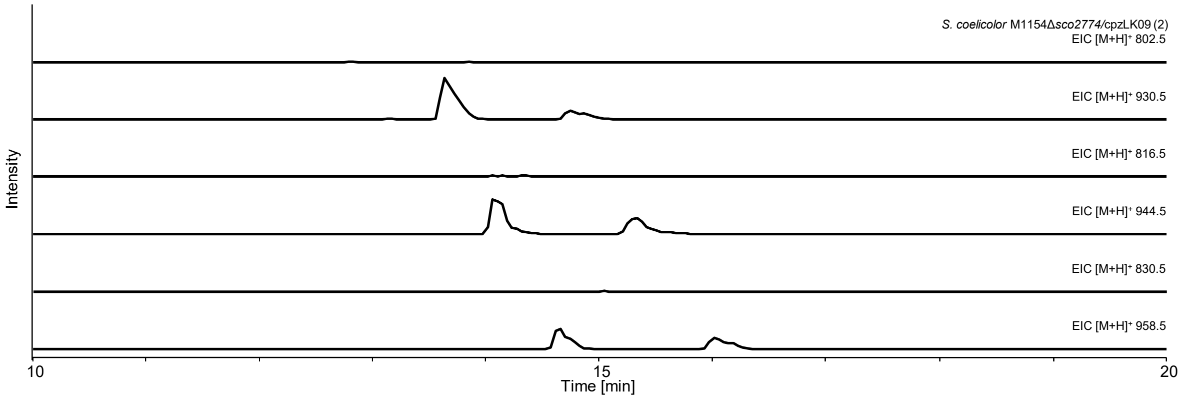

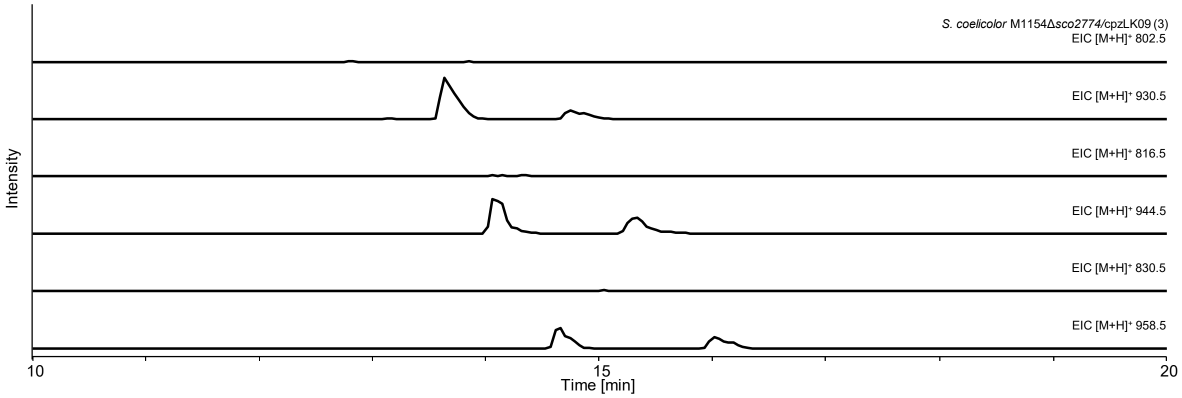

Figure S20: Extracted ion chromatograms of *S. coelicolor* M1154Δ*sco2774*/cpzLK09 (three individual mutants). Masses are shown for caprazamycin aglycons E/F with *m/z* of 930.5, caprazamycin aglycons C/D/G with *m/z* of 944.5, caprazamycin aglycons A/B with *m/z* of 958.5 and the respective hydroxyacylcaprazols E/F with *m/z* of 802.5, hydroxyacylcaprazols C/D/G with *m/z of* 816.5 and hydroxyacylcaprazols A/B with *m/z* of 830.5.

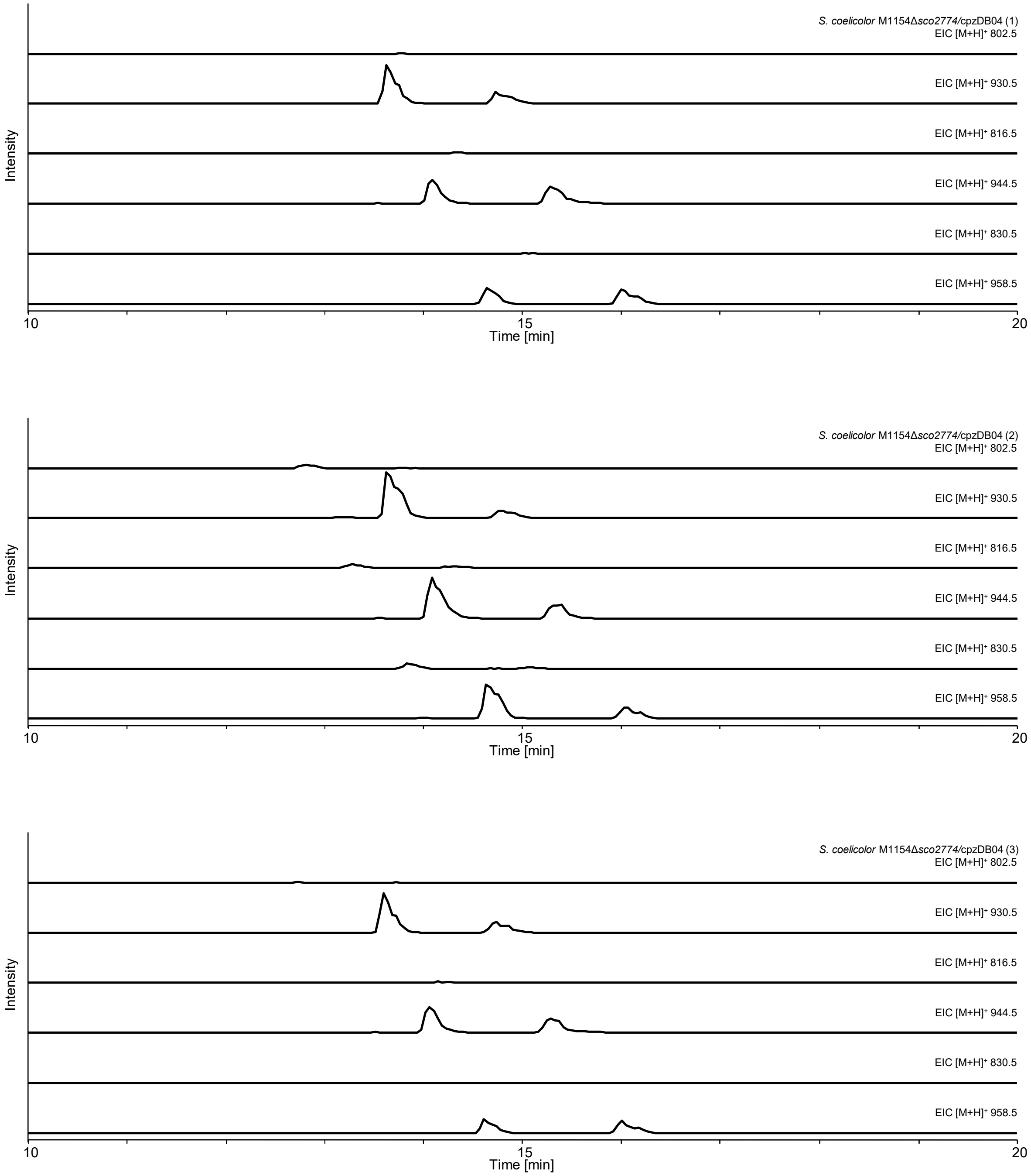

Figure S21: Extracted ion chromatograms of *S. coelicolor* M1154Δ*sco2774*/cpzDB04 (three individual mutants). Masses are shown for caprazamycin aglycons E/F with *m/z* of 930.5, caprazamycin aglycons C/D/G with *m/z* of 944.5, caprazamycin aglycons A/B with *m/z* of 958.5 and the respective hydroxyacylcaprazols E/F with *m/z* of 802.5, hydroxyacylcaprazols C/D/G with *m/z of* 816.5 and hydroxyacylcaprazols A/B with *m/z* of 830.5.

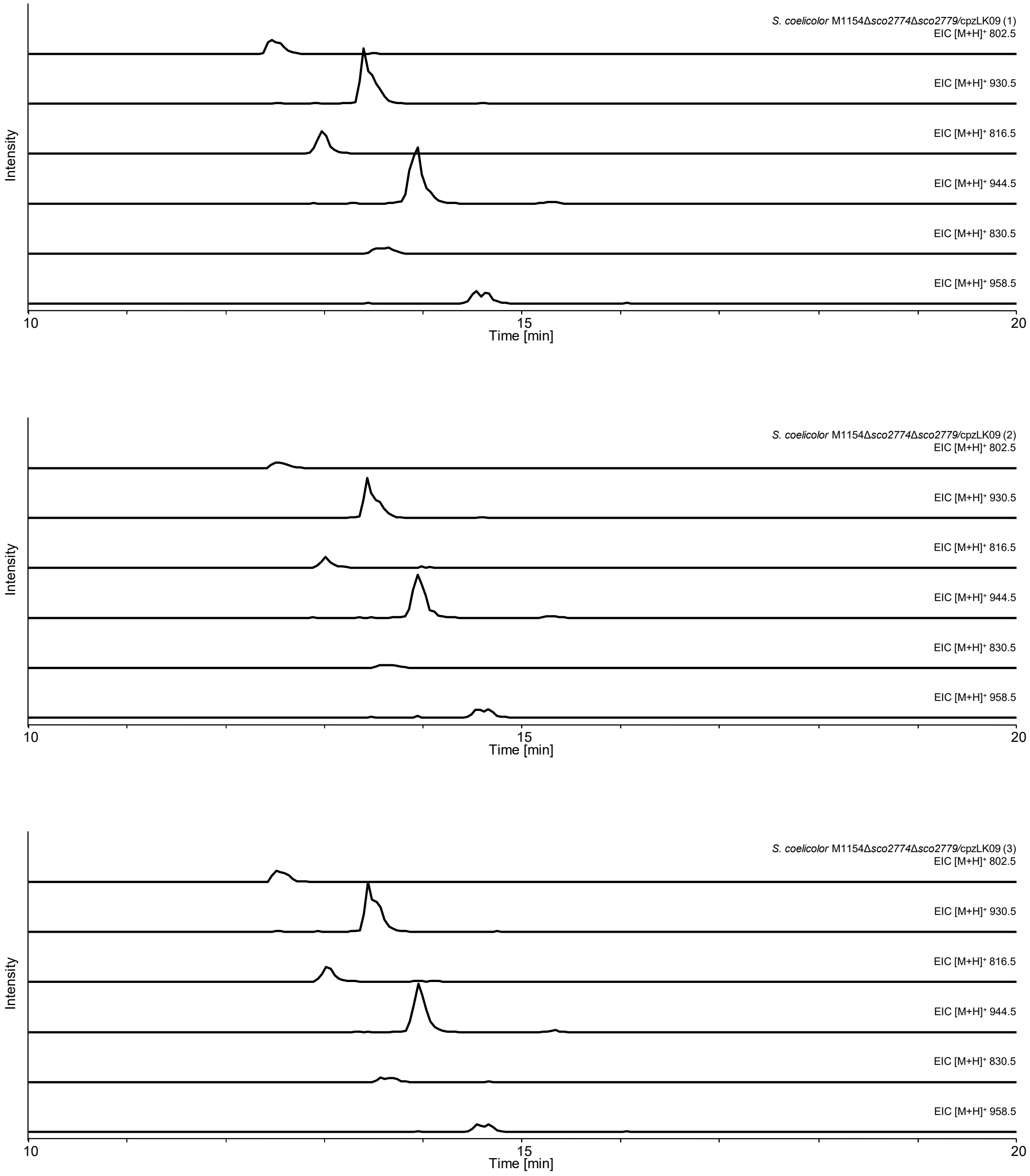

Figure S22: Extracted ion chromatograms of *S. coelicolor* M1154Δ*sco2774*Δ*sco2779*/cpzLK09 (three individual mutants). Masses are shown for caprazamycin aglycons E/F with *m/z* of 930.5, caprazamycin aglycons C/D/G with *m/z* of 944.5, caprazamycin aglycons A/B with *m/z* of 958.5 and the respective hydroxyacylcaprazols E/F with *m/z* of 802.5, hydroxyacylcaprazols C/D/G with *m/z of* 816.5 and hydroxyacylcaprazols A/B with *m/z* of 830.5.

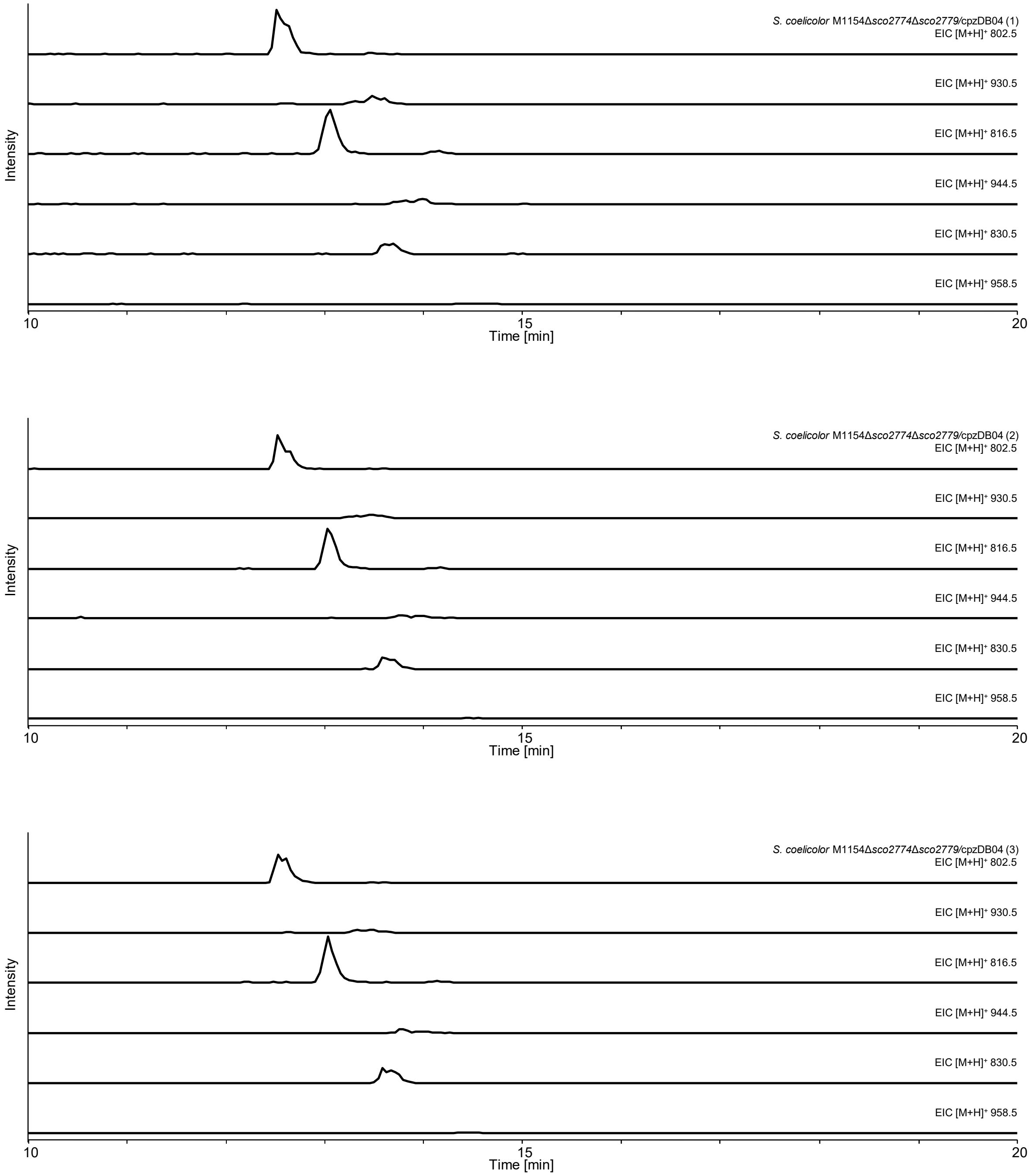

Figure S23: Extracted ion chromatograms of *S. coelicolor* M1154Δ*sco2774*Δ*sco2779*/cpzDB04 (three individual mutants). Masses are shown for caprazamycin aglycons E/F with *m/z* of 930.5, caprazamycin aglycons C/D/G with *m/z* of 944.5, caprazamycin aglycons A/B with *m/z* of 958.5 and the respective hydroxyacylcaprazols E/F with *m/z* of 802.5, hydroxyacylcaprazols C/D/G with *m/z of* 816.5 and hydroxyacylcaprazols A/B with *m/z* of 830.5.

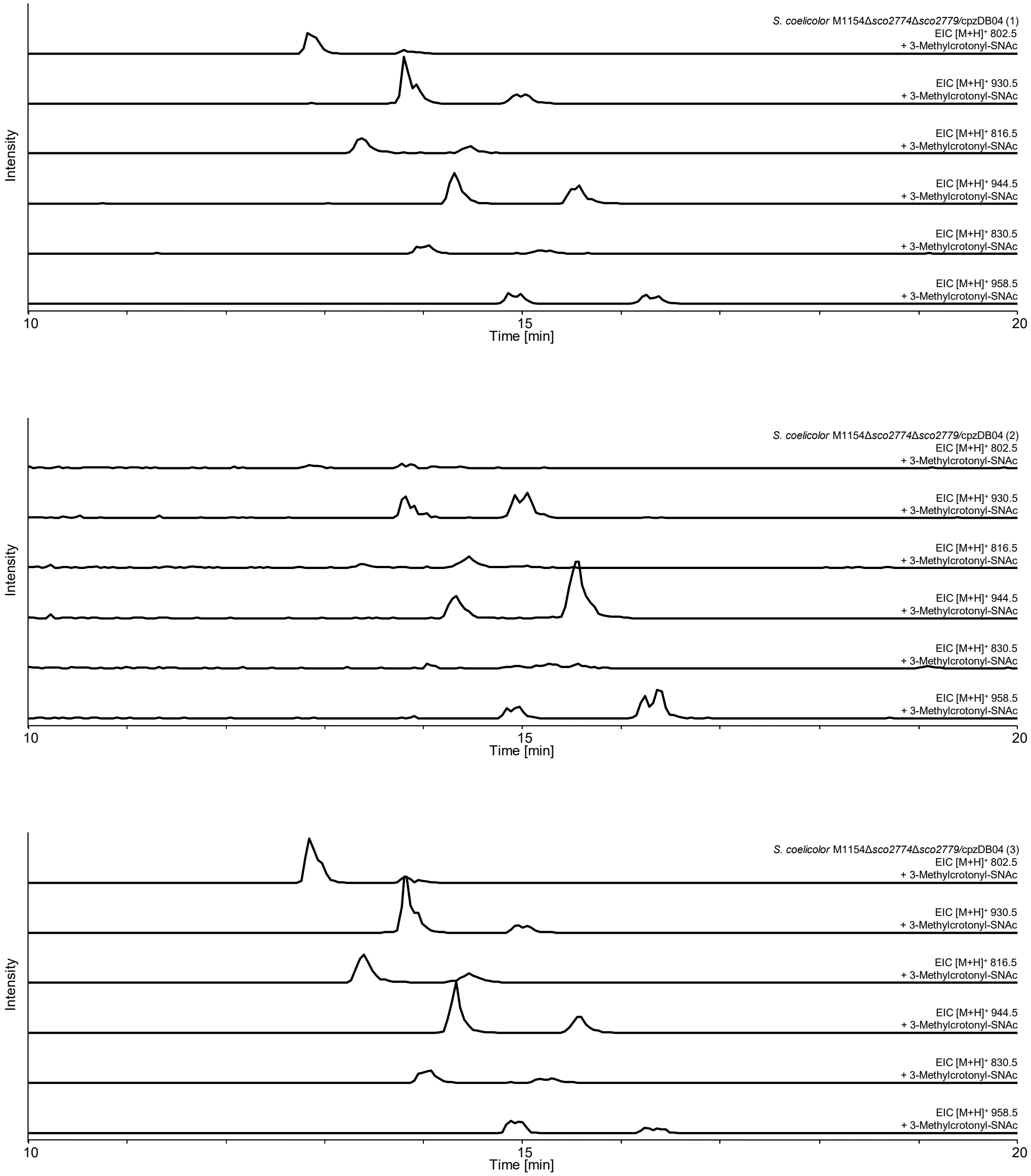

Figure S24: Extracted ion chromatograms of *S. coelicolor* M1154Δ*sco2774* Δ*sco2779*/cpzDB04 (three individual mutants). Cultures were supplied with 3-methylglutaconyl-CoA. Masses are shown for caprazamycin aglycons E/F with *m/z* of 930.5, caprazamycin aglycons C/D/G with *m/z* of 944.5, caprazamycin aglycons A/B with *m/z* of 958.5 and the respective hydroxyacylcaprazols E/F with *m/z* of 802.5, hydroxyacylcaprazols C/D/G with *m/z of* 816.5 and hydroxyacylcaprazols A/B with *m/z* of 830.5.

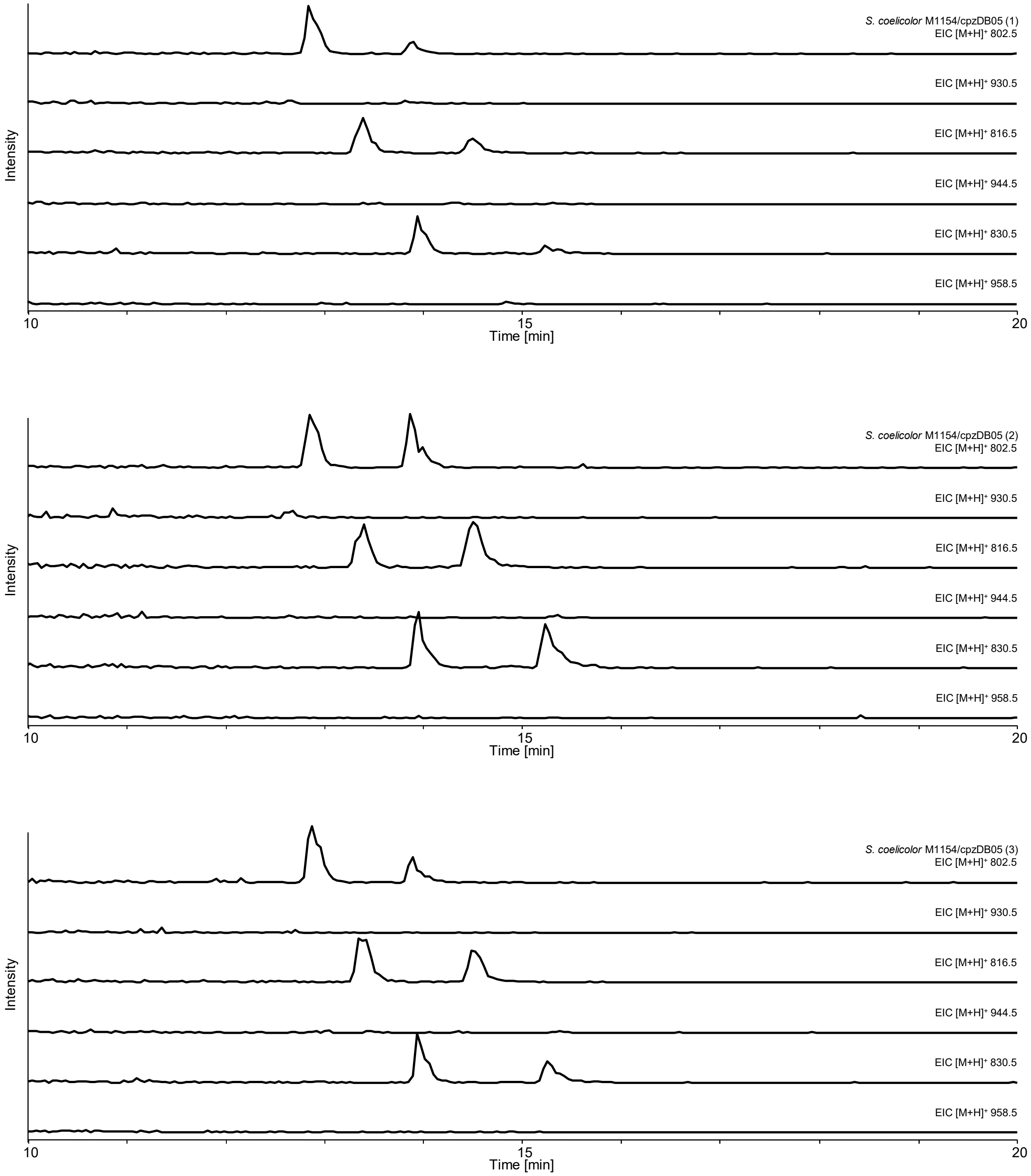

Figure S25: Extracted ion chromatograms of *S. coelicolor* M1154/cpzDB05 (three individual mutants). Masses are shown for caprazamycin aglycons E/F with *m/z* of 930.5, caprazamycin aglycons C/D/G with *m/z* of 944.5, caprazamycin aglycons A/B with *m/z* of 958.5 and the respective hydroxyacylcaprazols E/F with *m/z* of 802.5, hydroxyacylcaprazols C/D/G with *m/z of* 816.5 and hydroxyacylcaprazols A/B with *m/z* of 830.5.

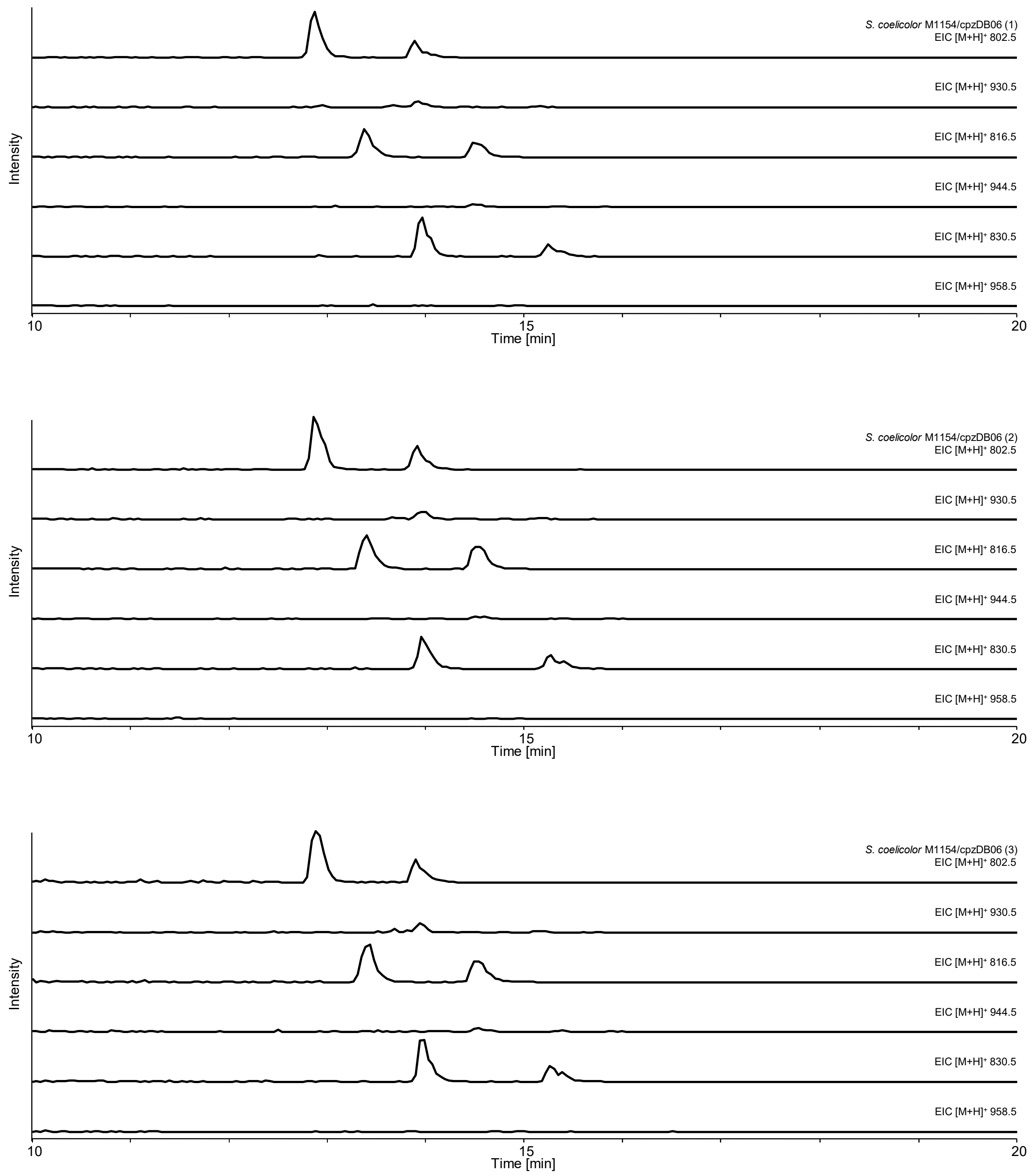

Figure S26: Extracted ion chromatograms of *S. coelicolor* M1154/cpzDB06 (three individual mutants). Masses are shown for caprazamycin aglycons E/F with *m/z* of 930.5, caprazamycin aglycons C/D/G with *m/z* of 944.5, caprazamycin aglycons A/B with *m/z* of 958.5 and the respective hydroxyacylcaprazols E/F with *m/z* of 802.5, hydroxyacylcaprazols C/D/G with *m/z of* 816.5 and hydroxyacylcaprazols A/B with *m/z* of 830.5.

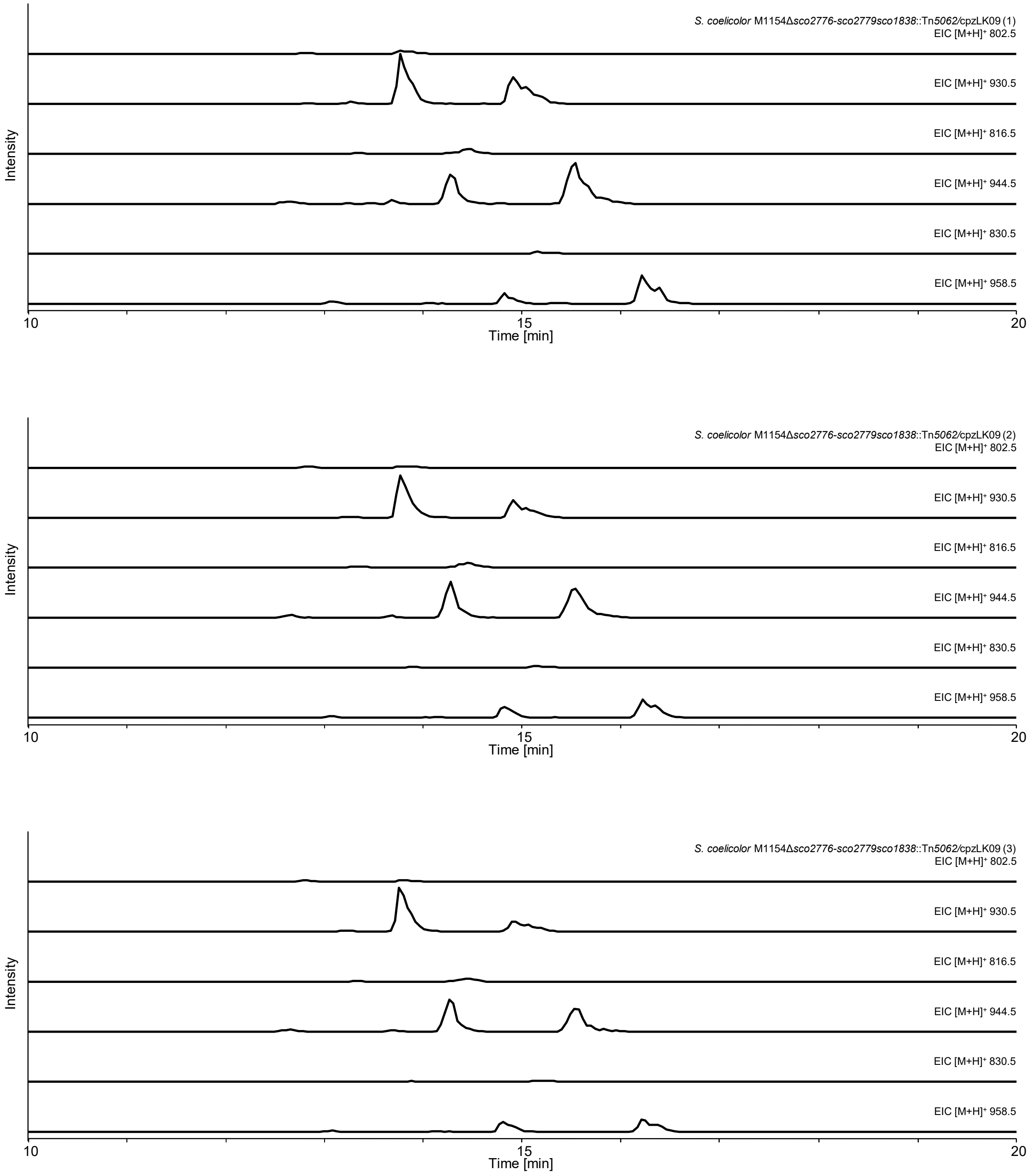

Figure S27: Extracted ion chromatograms of *S. coelicolor* M1154Δ*sco2776-sco2779sco1838*::Tn*5062*/cpzLK09 (three individual mutants). Masses are shown for caprazamycin aglycons E/F with *m/z* of 930.5, caprazamycin aglycons C/D/G with *m/z* of 944.5, caprazamycin aglycons A/B with *m/z* of 958.5 and the respective hydroxyacylcaprazols E/F with *m/z* of 802.5, hydroxyacylcaprazols C/D/G with *m/z of* 816.5 and hydroxyacylcaprazols A/B with *m/z* of 830.5.

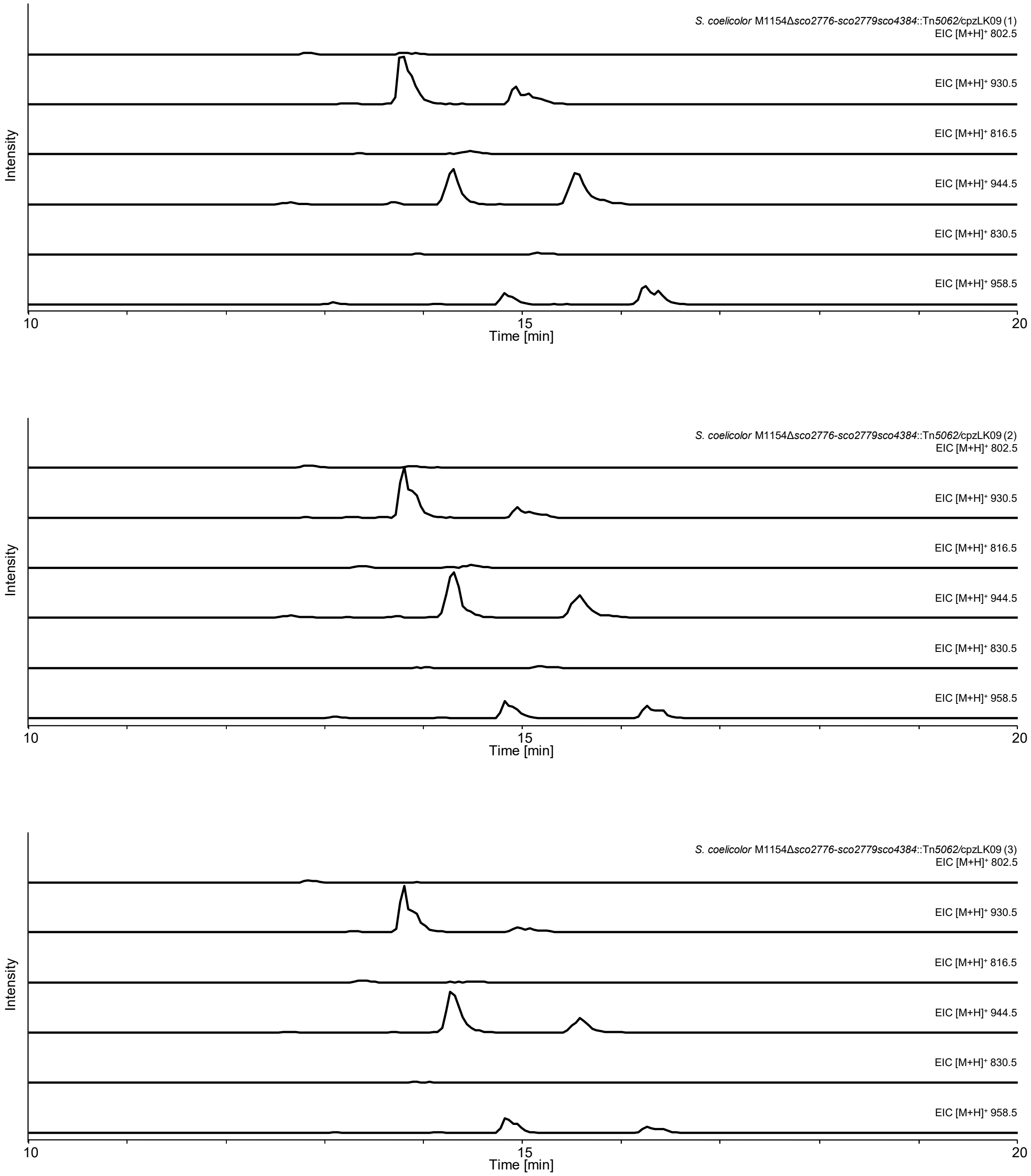

Figure S28: Extracted ion chromatograms of *S. coelicolor* M1154Δ*sco2776-sco2779sco4384*::Tn*5062*/cpzLK09 (three individual mutants). Masses are shown for caprazamycin aglycons E/F with *m/z* of 930.5, caprazamycin aglycons C/D/G with *m/z* of 944.5, caprazamycin aglycons A/B with *m/z* of 958.5 and the respective hydroxyacylcaprazols E/F with *m/z* of 802.5, hydroxyacylcaprazols C/D/G with *m/z of* 816.5 and hydroxyacylcaprazols A/B with *m/z* of 830.5.

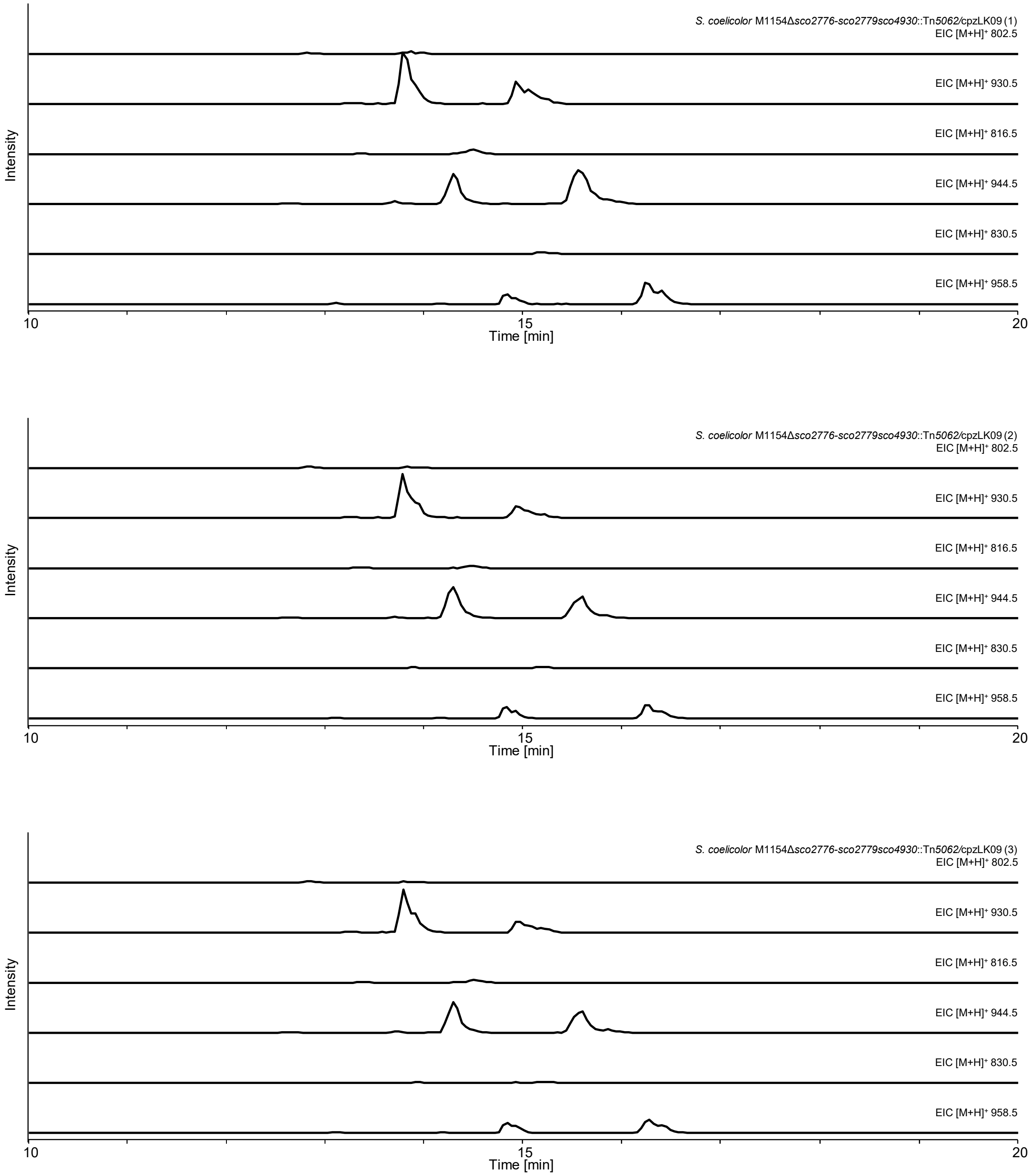

Figure S29: Extracted ion chromatograms of *S. coelicolor* M1154Δ*sco2776-sco2779sco4930*::Tn*5062*/cpzLK09 (three individual mutants). Masses are shown for caprazamycin aglycons E/F with *m/z* of 930.5, caprazamycin aglycons C/D/G with *m/z* of 944.5, caprazamycin aglycons A/B with *m/z* of 958.5 and the respective hydroxyacylcaprazols E/F with *m/z* of 802.5, hydroxyacylcaprazols C/D/G with *m/z of* 816.5 and hydroxyacylcaprazols A/B with *m/z* of 830.5.

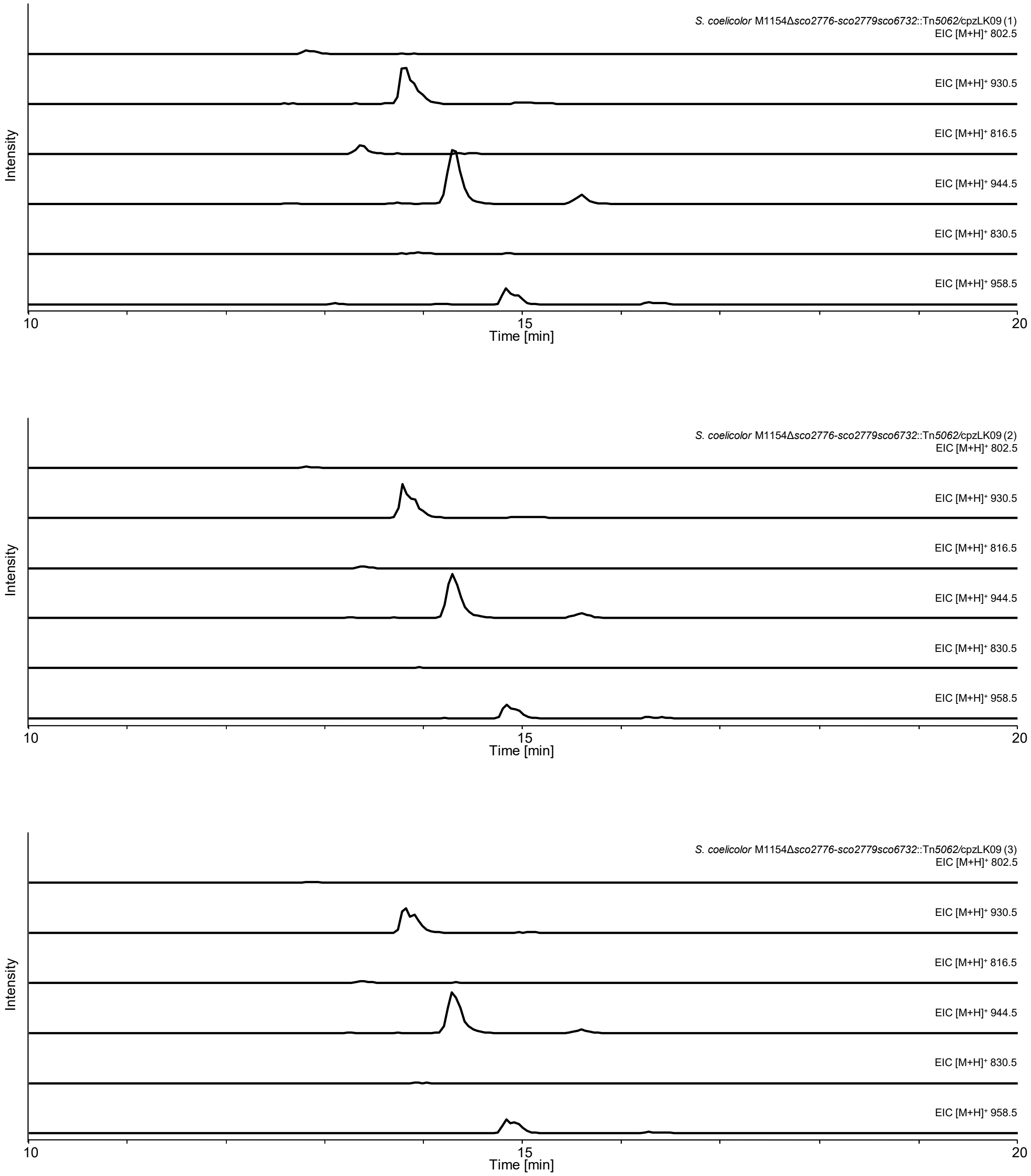

Figure S30: Extracted ion chromatograms of *S. coelicolor* M1154Δ*sco2776-sco2779sco6732*::Tn*5062*/cpzLK09 (three individual mutants). Masses are shown for caprazamycin aglycons E/F with *m/z* of 930.5, caprazamycin aglycons C/D/G with *m/z* of 944.5, caprazamycin aglycons A/B with *m/z* of 958.5 and the respective hydroxyacylcaprazols E/F with *m/z* of 802.5, hydroxyacylcaprazols C/D/G with *m/z of* 816.5 and hydroxyacylcaprazols A/B with *m/z* of 830.5.

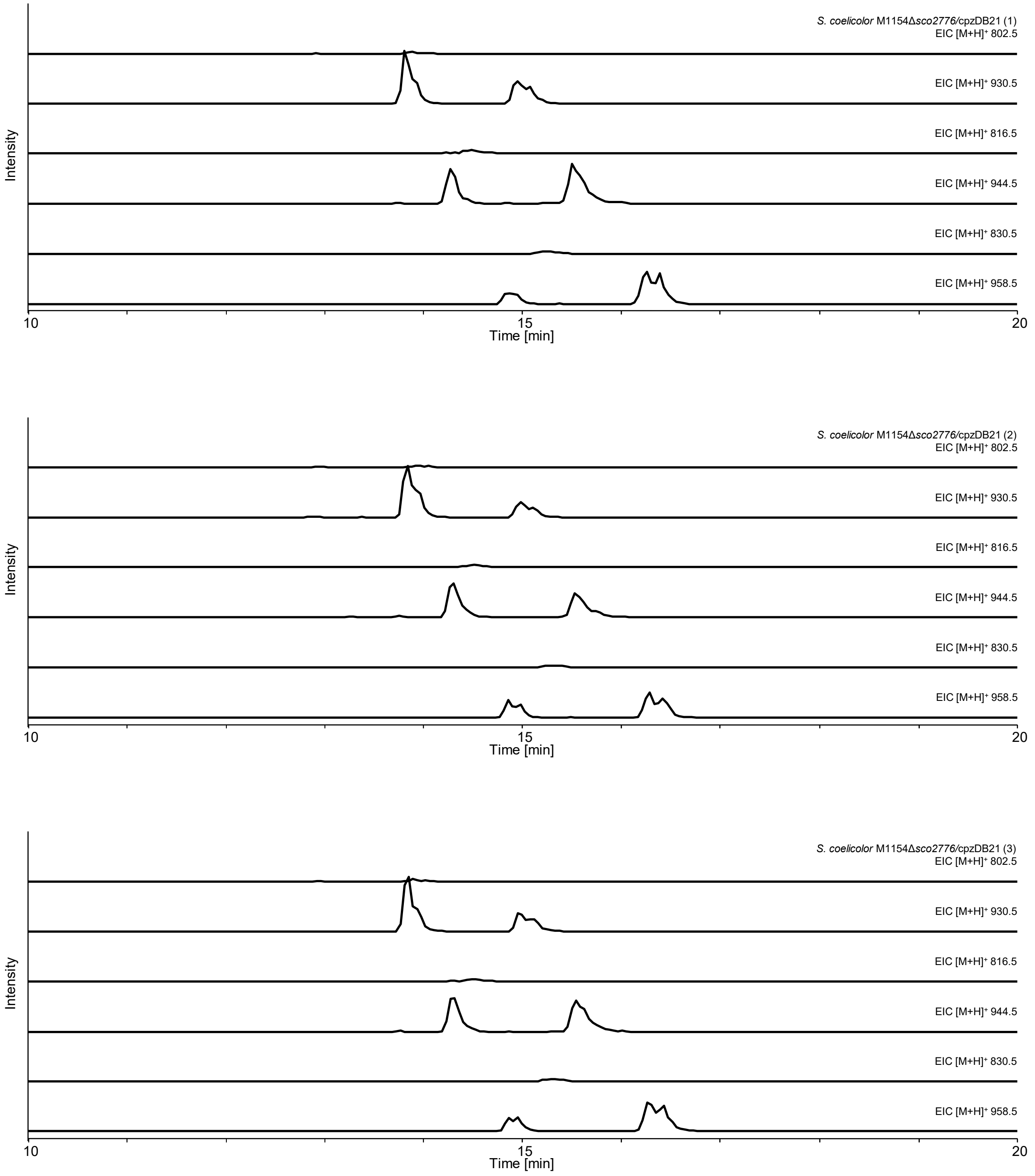

Figure S31: Extracted ion chromatograms of *S. coelicolor* M1154Δ*sco2777*/cpzDB21 (three individual mutants). Masses are shown for caprazamycin aglycons E/F with *m/z* of 930.5, caprazamycin aglycons C/D/G with *m/z* of 944.5, caprazamycin aglycons A/B with *m/z* of 958.5 and the respective hydroxyacylcaprazols E/F with *m/z* of 802.5, hydroxyacylcaprazols C/D/G with *m/z of* 816.5 and hydroxyacylcaprazols A/B with *m/z* of 830.5.

A Synthesis of 3-methylcrotonyl-SNAc (1)

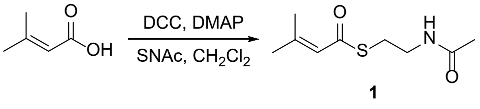

B Synthesis of (1-^{13}C)-3-methylcrotonyl-SNAc (5)

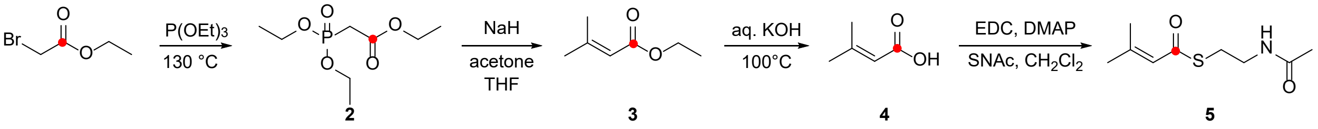

Figure S32: Syntheses of SNAc-thioesters for feeding experiments. **A**: 3-methylcrotonyl-SNAc (1). **B**: (1-^{13}C)-3-methylcrotonyl-SNAc (5).

Table S1: Plasmids, cosmids and strains

**Plasmids, cosmids, strains**	**Description and relevant characteristics**	**Reference**
pIJ773	*aac(3)IV*, *oriT*; Amp^R, Apr^R	Gust *et al.*, 2003
pIJ790	λ-RED (*gam*, *bet*, *exo*), *cat*, *araC*, *rep101*^{ts}; Cml^R	Gust *et al.*, 2003
pIJ787	*oriT*, *tet*, *attP*, *int*; Tet^R	Gust *et al.*, 2004
pR9406	derived from pUB307, *tra*, *oriT*; Amp^R, Cml^R	David Figurski
StC105	Cosmid from *S. coelicolor* genomic library carrying the Liu-pathway genes *sco2774-sco2779*; Amp^R, Kan^R	Redenbach *et al.*, 1996
StC105DB09	StC105 with a deletion of *sco2779* and *bla* replaced by *tet*, *oriT*; Kan^R, Tet^R	this study
StC105DB10	StC105 with a deletion of *sco2776* and *bla* replaced by *tet*, *oriT*; Kan^R, Tet^R	this study
StC105DB11	StC105 with a deletion of *sco2777* and *bla* replaced by *tet*, *oriT*; Kan^R, Tet^R	this study
StC105DB12	StC105 with a deletion of *sco2778* and *bla* replaced by *tet*, *oriT*; Kan^R, Tet^R	this study
StC105DB14	StC105 with a deletion of *sco2776-sco2779* and *bla* replaced by *tet*, *oriT*; Kan^R, Tet^R	this study
StC105DB21	StC105 with a deletion of *sco2774*, *sco2779* and *bla* replaced by *tet*, *oriT*; Kan^R, Tet^R	this study
StC105DB22	StC105 with a deletion of *sco2774* and *bla* replaced by *tet*, *oriT*; Kan^R, Tet^R	this study
SCI8.1.H03	Cosmid from *S. coelicolor* genomic library carrying *sco1838* disrupted by Tn*5062*; Amp^R, Apr^R, Kan^R	Bishop *et al.*, 2004
2D52.2.F12	Cosmid from *S. coelicolor* genomic library carrying *sco4384* disrupted by Tn*5062*; Amp^R, Apr^R, Kan^R	Bishop *et al.*, 2004
2SCK31.2.D06	Cosmid from *S. coelicolor* genomic library carrying *sco4930* disrupted by Tn*5062*; Amp^R, Apr^R, Kan^R	Bishop *et al.*, 2004
5F2A.2.H07	Cosmid from *S. coelicolor* genomic library carrying *sco6732* disrupted by Tn*5062*; Amp^R, Apr^R, Kan^R	Bishop *et al.*, 2004
cpzLK09	Cosmid containing the caprazamycin biosynthetic gene cluster from *Streptomyces sp.* MK730-62F2, *oriT*, *tet*, *attP*, *int*; Tet^R, Kan^R	Kaysser *et al.*, 2009
cpzDB04	cpzLK09 with a deletion of *cpz5*; Tet^R, Kan^R	this study
cpzDB05	cpzLK09 with a deletion of *cpz20*; Tet^R, Kan^R	this study
cpzDB06	cpzLK09 with a deletion of *cpz25*; Tet^R, Kan^R	this study
cpzDB21	cpzLK09 with a deletion of *cpz2*; Tet^R, Kan^R	this study
E. coli DH5α	F^-, Φ80*lac*ZΔM15, Δ(*lac*ZYA-*arg*F)U169, *rec*A1, *end*A1, *hsd*R17(r_k^-, m_k^+), *pho*A, *sup*E44, *thi*-1, *gyr*A96, *rel*A1, λ^-	Thermo Fisher Scientific
E. coli BW25113	*lacI*^{q}, *rrnB*$_{T14}$, Δ*lacZ*$_{WJ16}$, *hsdR514*, Δ*araBAD*$_{AH33}$, Δ*rhaBAD*$_{LD78}$	Datsenko & Wanner, 2000
E. coli ET12567	*dam-13::*Tn*9, dcm-6, hsdM*$^-$; Cml^R, Tet^R	MacNeil *et al.*, 1992
E. coli BT340	*E. coli* DH5α harbouring pCP20 (*FLP*$^+$, λ cI857$^+$, λ p_R Rep^{ts}); Amp^R, Cml^R	Cherepanov & Wackernagel, 1995
Streptomyces coelicolor M1154	Δ*act*, Δ*red*, Δ*cpk*, Δ*cda*, *rpoB* (C1298T), *rpsL* (A262G)	Gomez-Escribano *et al.*, 2011
Streptomyces coelicolor M1154/cpzLK09 (1)-(3)	*S. coelicolor* M1154 harbouring the caprazamycin gene cluster, three independent mutants; Tet^R, Kan^R	this study
Streptomyces coelicolor M1154/cpzDB04 (1)-(3)	*S. coelicolor* M1154 harbouring a *cpz5* deficient caprazamycin gene cluster, three independent mutants; Tet^R, Kan^R	this study
Streptomyces coelicolor M1154/cpzDB05 (1)-(3)	*S. coelicolor* M1154 harbouring a *cpz20* deficient caprazamycin gene cluster, three independent mutants; Tet^R, Kan^R	this study
Streptomyces coelicolor M1154/cpzDB06 (1)-(3)	*S. coelicolor* M1154 harbouring a *cpz25* deficient caprazamycin gene cluster, three independent mutants; Tet^R, Kan^R	this study
Streptomyces coelicolor M1154Δ*sco2774*	*S. coelicolor* M1154 with a deletion of *sco2774*	this study
Streptomyces coelicolor M1154Δ*sco2776*	*S. coelicolor* M1154 with a deletion of *sco2776*	this study
Streptomyces coelicolor M1154Δ*sco2777*	*S. coelicolor* M1154 with a deletion of *sco2777*	this study
Streptomyces coelicolor M1154Δ*sco2778*	*S. coelicolor* M1154 with a deletion of *sco2778*	this study
Streptomyces coelicolor M1154Δ*sco2779*	*S. coelicolor* M1154 with a deletion of *sco2779*	this study

Streptomyces coelicolor M1154Δ*sco2774*Δ*sco2779*	*S. coelicolor* M1154 with a deletion of *sco2774* and *sco2779*	this study
Streptomyces coelicolor M1154Δ*sco2776-sco2779*	*S. coelicolor* M1154 with a deletion of *sco2776-sco2779*	this study
Streptomyces coelicolor M1154Δ*sco2776-sco2779*/*sco1838*::Tn*5062*	*S. coelicolor* M1154 with a deletion of *sco2776-sco2779* and an inactivation of *sco1838* by transposon insertion	this study
Streptomyces coelicolor M1154Δ*sco2776-sco2779*/*sco4384*::Tn*5062*	*S. coelicolor* M1154 with a deletion of *sco2776-sco2779* and an inactivation of *sco4384* by transposon insertion	this study
Streptomyces coelicolor M1154Δ*sco2776-sco2779*/*sco4930*::Tn*5062*	*S. coelicolor* M1154 with a deletion of *sco2776-sco2779* and an inactivation of *sco4930* by transposon insertion	this study
Streptomyces coelicolor M1154Δ*sco2776-sco2779*/*sco6732*::Tn*5062*	*S. coelicolor* M1154 with a deletion of *sco2776-sco2779* and an inactivation of *sco6732* by transposon insertion	this study
Streptomyces coelicolor M1154Δ*sco2774*/cpzLK09 (1)-(3)	*S. coelicolor* M1154 with a deletion of *sco2774* harbouring the caprazamycin gene cluster, three independent mutants; Tet^R, Kan^R	this study
Streptomyces coelicolor M1154Δ*sco2774*/cpzDB04 (1)-(3)	*S. coelicolor* M1154 with a deletion of *sco2774* harbouring a *cpz5* deficient caprazamycin gene cluster, three independent mutants; Tet^R, Kan^R	this study
Streptomyces coelicolor M1154Δ*sco2776*/cpzLK09 (1)-(3)	*S. coelicolor* M1154 with a deletion of *sco2776* harbouring the caprazamycin gene cluster, three independent mutants; Tet^R, Kan^R	this study
Streptomyces coelicolor M1154Δ*sco2776*/cpzDB04 (1)-(3)	*S. coelicolor* M1154 with a deletion of *sco2776* harbouring a *cpz5* deficient caprazamycin gene cluster, three independent mutants; Tet^R, Kan^R	this study
Streptomyces coelicolor M1154Δ*sco2777*/cpzLK09 (1)-(3)	*S. coelicolor* M1154 with a deletion of *sco2777* harbouring the caprazamycin gene cluster, three independent mutants; Tet^R, Kan^R	this study
Streptomyces coelicolor M1154Δ*sco2777*/cpzDB04 (1)-(3)	*S. coelicolor* M1154 with a deletion of *sco2777* harbouring a *cpz5* deficient caprazamycin gene cluster, three independent mutants; Tet^R, Kan^R	this study
Streptomyces coelicolor M1154Δ*sco2778*/cpzLK09 (1)-(3)	*S. coelicolor* M1154 with a deletion of *sco2778* harbouring the caprazamycin gene cluster, three independent mutants; Tet^R, Kan^R	this study
Streptomyces coelicolor M1154Δ*sco2778*/cpzDB04 (1)-(3)	*S. coelicolor* M1154 with a deletion of *sco2778* harbouring a *cpz5* deficient caprazamycin gene cluster, three independent mutants; Tet^R, Kan^R	this study
Streptomyces coelicolor M1154Δ*sco2779*/cpzLK09 (1)-(3)	*S. coelicolor* M1154 with a deletion of *sco2779* harbouring the caprazamycin gene cluster, three independent mutants; Tet^R, Kan^R	this study
Streptomyces coelicolor M1154Δ*sco2779*/cpzDB04 (1)-(3)	*S. coelicolor* M1154 with a deletion of *sco2779* harbouring a *cpz5* deficient caprazamycin gene cluster, three independent mutants; Tet^R, Kan^R	this study
Streptomyces coelicolor M1154Δ*sco2774*/Δ*sco2779*/cpzLK09 (1)-(3)	*S. coelicolor* M1154 with a deletion of *sco2774* and *sco2779* harbouring the caprazamycin gene cluster, three independent mutants; Tet^R, Kan^R	this study
Streptomyces coelicolor M1154Δ*sco2774*/Δ*sco2779*/cpzDB04 (1)-(3)	*S. coelicolor* M1154 with a deletion of *sco2774* and *sco2779* harbouring a *cpz5* deficient caprazamycin gene cluster, three independent mutants; Tet^R, Kan^R	this study
Streptomyces coelicolor M1154Δ*sco2776-sco2779*/cpzLK09 (1)-(3)	*S. coelicolor* M1154 with a deletion of *sco2776-sco2779* harbouring the caprazamycin gene cluster, three independent mutants; Tet^R, Kan^R	this study
Streptomyces coelicolor M1154Δ*sco2776-sco2779*/cpzDB04 (1)-(3)	*S. coelicolor* M1154 with a deletion of *sco2776-sco2779* harbouring a *cpz5* deficient caprazamycin gene cluster, three independent mutants; Tet^R, Kan^R	this study
Streptomyces coelicolor M1154Δ*sco2777*/cpzDB21 (1)-(3)	*S. coelicolor* M1154 with a deletion of *sco2777* harbouring a *cpz2* deficient caprazamycin gene cluster, three independent mutants; Tet^R, Kan^R	this study
Streptomyces coelicolor M1154Δ*sco2776-sco2779*/*sco1838*::Tn*5062*/cpzLK09 (1)-(3)	*S. coelicolor* M1154 with a deletion of *sco2776-sco2779* and an inactivation of *sco1838* by transposon insertion harbouring the caprazamycin gene cluster, three independent mutants; Apr^R, Tet^R, Kan^R	this study
Streptomyces coelicolor M1154Δ*sco2776-sco2779*/*sco4384*::Tn*5062*/cpzLK09 (1)-(3)	*S. coelicolor* M1154 with a deletion of *sco2776-sco2779* and an inactivation of *sco4384* by transposon insertion harbouring the caprazamycin gene cluster, three independent mutants; Apr^R, Tet^R, Kan^R	this study
Streptomyces coelicolor M1154Δ*sco2776-sco2779*/*sco4930*::Tn*5062*/cpzLK09 (1)-(3)	*S. coelicolor* M1154 with a deletion of *sco2776-sco2779* and an inactivation of *sco4930* by transposon insertion harbouring the caprazamycin gene cluster, three independent mutants; Apr^R, Tet^R, Kan^R	this study
Streptomyces coelicolor M1154Δ*sco2776-sco2779*/*sco6732*::Tn*5062*/cpzLK09 (1)-(3)	*S. coelicolor* M1154 with a deletion of *sco2776-sco2779* and an inactivation of *sco6732* by transposon insertion harbouring the caprazamycin gene cluster, three independent mutants; Apr^R, Tet^R, Kan^R	this study

Table S2: Primer sequences

Name	Sequence 5‘ → 3‘
liuA_F	CGTACGAGCCCTCTCCCACCAGGAGTCGTGAACACCATGATTCCGGGGATCCGTCGACC
liuA_R	GGCCGGTGCGGCGCCCGCCCCCGCCGCCTCGGAGCCTCATGTAGGCTGGAGCTGCTTC
liuB_F	ATTAACTGGTCGACCACTGGGTGAGGGGACCGCAGGATGATTCCGGGGATCCGTCGACC
liuB_R	GCGGCGTGTCCTTCGTGTTGTCCATGGGTCCCCTCCTCATGTAGGCTGGAGCTGCTTC
liuD_F	AGGGGACCCATGGACAACACGAAGGACACGCCGCGCATGATTCCGGGGATCCGTCGACC
liuD_R	CGGCCATCGGGAGGCCCGGTTCCGGGGCGTTCATGCCGTTGTAGGCTGGAGCTGCTTC
liuE_F	ACCCACCGACGACGCGACGGAGGAGACGGCATGAACGCCATTCCGGGGATCCGTCGACC
liuE_R	CTTCCAGTTCGGGGGAGAGCTTGTGGTCCATGGTGTTCATGTAGGCTGGAGCTGCTTC
sco2774_773_FW	CAAGCGCTTAGATATTCGTACCCTGGAGGTGGCGCGGTGATTCCGGGGATCCGTCGACC
sco2774_773_RV	AGGAGGGACTGGAGTGCTTCGCTCATGACCTGTGGTCTATGTAGGCTGGAGCTGCTTC
LiuA_verify_1154_neu_F	GAAGACCTCGTGTGGATGCT
LiuA_verify_1154_neu_R	GAGGCGTACTTCTCGACGTT
LiuB_verify_1154_neu2_F	GGCGCCTATCTCGTCGAC
LiuB_verify_1154_neu2_R	CGACGGACAGATAGCTCTCC
LiuD_verify_1154_neu_F	GAGGAGGAGGAGTCCTTCAA
LiuD_verify_1154_neu_R	CACTTGGGGTGGACGAAG
LiuE_verify_1154_neu_F	GATGAAGATGGAGCACGTCA
LiuE_verify_1154_neu_R	ATGACCCATTCGTTCGTAGC
sco2774_verify_FW	AGGAAGTACGCGTGCAGG
sco2774_verify_RV	CTTCGGCCTCCTGAACTCC
bla-oriT_cassette_787	CCTGATAAATGCTTCAATAATATTGAAAAAGGAAGAGTCCGGCCAGCCTCGCAGAGCAG
bla-tet_cassette_787	ATCTAAAGTATATATGAGTAAACTTGGTCTGACAGTTATCAGGTCGAGGTGGCCCGGC
bla_tet_oriT_v4_F	TGAGTAAACTTGGTCTGACAGTT
bla_tet_oriT_v4_R	CCGCTCATGAGACAATAACCC
LiuA_in-out_1154_F	CACTTCCACGACACCTACG
LiuA_in-out_1154_R	GATCTCGTACGGGAACTCG
LiuB_in-out_1154_F	TTCTAGACAGGCGGGTTAGC
LiuB_in-out_1154_R	TCATCGGGTAGTACGTGCC
LiuD_in-out_1154_neu_F	CTTCCTGTGGATGTGGCCC
LiuD_in-out_1154_neu_R	TCGACGGACAGATAGCTCTCC
LiuE_in-out_1154_neu_F	ACAAGCGTCTGGATGGCC
LiuE_in-out_1154_neu_R	ATTCGTTCGTAGCCTCGTCC
sco2774_in-out_FW	CTCCTCGTACGTCTGGAAGG
sco2774_in-out_RV	TCGGCGAGGAGAACAAGG
SCO1838_tra_ver_FW	TCCTGGTACTCGGCGATGG

SCO1838_tra_ver_RV	CCTTGAAGAAGATGGTGCGC
SCO4384_tra_ver_FW	CCTCCGGCAAGATCCTCC
SCO4384_tra_ver_RV	GAACTTCAGGGTCAGCTTGC
SCO4930_tra_ver_FW	TGTCACTCGAACGGGTAAGC
SCO4930_tra_ver_RV	CTTGGCAAGGTCATGATGGG
SCO6732_tra_ver_FW	AGGACATGCTCGCCAAGG
SCO6732_tra_ver_RV	GAACTTCAGGGTCAGCTTGC
1383_gene_RV	CTGGAGGGGTGATCATGAGG
4384_gene_RV	TCACACCACCCATTCCGG
4930_gene_RV	GCCGTGAACACCAGCTCG
6732_gene_RV	CCTTCTCCTGACTCGTACGG
cpz2_773_FW	CGTCCGTACCCGCTACGGCAAGAGCTAAGGATCCCCATGATTCCGGGGATCCGTCGACC
cpz2_773_RV	CTCGGAACGCCTTCTGGACCTGGTGCCGGCGCGACCTCATGTAGGCTGGAGCTGCTTC
cpz5_773_F	CCGGGACAACGAGACAGCAAAGGTCGGTGTTTGGGACATGATTCCGGGGATCCGTCGACC
cpz5_773_R	GCGGACCAACACAGTCATCTCCCTCGGACGTGGACGGTCATGTAGGCTGGAGCTGCTTC
cpz20_773_F	GTCCGCACCGACGAAACGGGGACCGTTCATGTCTGACGGGATTCCGGGGATCCGTCGACC
cpz20_773_R	ACAGGGACCTTCTGCCCGTCTCGGCCGTTCGCCATCAGTATGTAGGCTGGAGCTGCTTC
cpz25_773_F	GTGGGAACGCCATCCGGCAGCCCGAGGACGACGACTCATGATTCCGGGGATCCGTCGACC
cpz25_773_R	ATGGTCTGCATGTGCGCTCCGCGCGGGTCCTGGGCCGTCATGTAGGCTGGAGCTGCTTC
cpz2_verify_FW	AGGACATGATCATCTCGGGC
cpz2_verify_RV	GCCAGATGATCGAGGAGACC
cpz5_verifyKO_F	ACGAGCAGGGCCATCAAGC
cpz5_verifyKO_R	ACCTCGCCGCGATCATCC
cpz20_verifyKO_F	CGAGGTGCTCCACGACG
cpz20_verifyKO_R	GGAGCTGCCAGGACATGC
cpz25_verifyKO_F	GTGGTGTCCGGATTCTCG
cpz25_verifyKO_R	TTCTCGGCCAGTGTTTCC

References

1. Chavez-Aviles M, Diaz-Perez AL, Reyes-de la Cruz H, Campos-Garcia J. The Pseudomonas aeruginosa liuE gene encodes the 3-hydroxy-3-methylglutaryl coenzyme A lyase, involved in leucine and acyclic terpene catabolism. FEMS Microbiol Lett. 2009;296(1):117-23.

2. McErlean M, Liu X, Cui Z, Gust B, Van Lanen SG. Identification and characterization of enzymes involved in the biosynthesis of pyrimidine nucleoside antibiotics. Nat Prod Rep. 2021;38(7):1362-407.

3. Gust B, Challis GL, Fowler K, Kieser T, Chater KF. PCR-targeted Streptomyces gene replacement identifies a protein domain needed for biosynthesis of the sesquiterpene soil odor geosmin. Proc Natl Acad Sci U S A. 2003;100(4):1541-6.

4. Gust B, Chandra G, Jakimowicz D, Yuqing T, Bruton CJ, Chater KF. Lambda red-mediated genetic manipulation of antibiotic-producing Streptomyces. Adv Appl Microbiol. 2004;54:107-28.

5. Redenbach M, Kieser HM, Denapaite D, Eichner A, Cullum J, Kinashi H, et al. A set of ordered cosmids and a detailed genetic and physical map for the 8 Mb Streptomyces coelicolor A3(2) chromosome. Mol Microbiol. 1996;21(1):77-96.

6. Bishop A, Fielding S, Dyson P, Herron P. Systematic insertional mutagenesis of a streptomycete genome: a link between osmoadaptation and antibiotic production. Genome Res. 2004;14(5):893-900.

7. Kaysser L, Lutsch L, Siebenberg S, Wemakor E, Kammerer B, Gust B. Identification and manipulation of the caprazamycin gene cluster lead to new simplified liponucleoside antibiotics and give insights into the biosynthetic pathway. J Biol Chem. 2009;284(22):14987-96.

8. Datsenko KA, Wanner BL. One-step inactivation of chromosomal genes in Escherichia coli K-12 using PCR products. Proc Natl Acad Sci U S A. 2000;97(12):6640-5.

9. MacNeil DJ, Gewain KM, Ruby CL, Dezeny G, Gibbons PH, MacNeil T. Analysis of Streptomyces avermitilis genes required for avermectin biosynthesis utilizing a novel integration vector. Gene. 1992;111(1):61-8.

10. Cherepanov PP, Wackernagel W. Gene disruption in Escherichia coli: TcR and KmR cassettes with the option of Flp-catalyzed excision of the antibiotic-resistance determinant. Gene. 1995;158(1):9-14.

11. Gomez-Escribano JP, Bibb MJ. Engineering Streptomyces coelicolor for heterologous expression of secondary metabolite gene clusters. Microb Biotechnol. 2011;4(2):207-15.
